# Chemical Library Screening and Structure-Function Relationship Studies Identify Bisacodyl as a Potent and Selective Cytotoxic Agent Towards Quiescent Human Glioblastoma Tumor Stem-Like Cells

**DOI:** 10.1371/journal.pone.0134793

**Published:** 2015-08-13

**Authors:** Maria Zeniou, Marie Fève, Samir Mameri, Jihu Dong, Christophe Salomé, Wanyin Chen, Elias A. El-Habr, Fanny Bousson, Mohamadou Sy, Julie Obszynski, Alexandre Boh, Pascal Villa, Suzana Assad Kahn, Bruno Didier, Dominique Bagnard, Marie-Pierre Junier, Hervé Chneiweiss, Jacques Haiech, Marcel Hibert, Marie-Claude Kilhoffer

**Affiliations:** 1 Laboratoire d’Innovation Thérapeutique, Université de Strasbourg / CNRS UMR7200, Laboratoire d’Excellence Medalis, Faculté de Pharmacie, 74 route du Rhin, 67401 Illkirch, France; 2 Neuroscience Paris Seine-IBPS, CNRS UMR 8246/ Inserm U1130/ UPMC UMCR18, 7 quai Saint Bernard, 75005 Paris, France; 3 Plateforme de Chimie Biologie Intégrative (PCBIS), Université de Strasbourg / CNRS UMS 3286, Laboratoire d’Excellence Medalis, ESBS Pôle API-Bld Sébastien Brant, 67401 Illkirch, France; 4 U682, Inserm, Université de Strasbourg, 3, Avenue Molière, 67200 Strasbourg, France; Spanish National Cancer Centre (CNIO), SPAIN

## Abstract

Cancer stem-like cells reside in hypoxic and slightly acidic tumor niches. Such microenvironments favor more aggressive undifferentiated phenotypes and a slow growing "quiescent state" which preserves them from chemotherapeutic agents that essentially target proliferating cells. Our objective was to identify compounds active on glioblastoma stem-like cells, including under conditions that mimick those found *in vivo* within this most severe and incurable form of brain malignancy. We screened the Prestwick Library to identify cytotoxic compounds towards glioblastoma stem-like cells, either in a proliferating state or in more slow-growing "quiescent" phenotype resulting from non-renewal of the culture medium *in vitro*. Compound effects were assessed by ATP-level determination using a cell-based assay. Twenty active molecules belonging to different pharmacological classes have thus been identified. Among those, the stimulant laxative drug bisacodyl was the sole to inhibit in a potent and specific manner the survival of quiescent glioblastoma stem-like cells. Subsequent structure-function relationship studies led to identification of 4,4'-dihydroxydiphenyl-2-pyridyl-methane (DDPM), the deacetylated form of bisacodyl, as the pharmacophore. To our knowledge, bisacodyl is currently the only known compound targeting glioblastoma cancer stem-like cells in their quiescent, more resistant state. Due to its known non-toxicity in humans, bisacodyl appears as a new potential anti-tumor agent that may, in association with classical chemotherapeutic compounds, participate in tumor eradication.

## Introduction

Because of their location, invasiveness and resistance to standard therapies, treating malignant brain tumors is challenging. This is especially true for glioblastoma (GBM), the most common and high grade form of glioma [1, 2]. Current glioblastoma treatments combine surgery, radiotherapy and chemotherapy with temozolomide, a DNA-alkylating agent [3]. Despite multiple therapeutic approaches, median survival of glioblastoma patients rarely exceeds 2 years [4].

Glioblastomas are histopathologically heterogeneous with cells characterized by various degrees of proliferative ability, differentiation and/or invasiveness. In the past years, the cancer stem cell model was proposed to explain tumor heterogeneity [5–7]. A subpopulation of malignant cancer stem-like cells, with tumor-propagating, self-renewal and differentiation capacities, was first isolated and characterized from hematopoietic malignancies [8] and subsequently from solid tumors of the brain and other organs [6]. In addition, numerous studies support the participation of cancer stem-like cells in tumor recurrences after treatment. Glioblastoma stem-like cells (GSCs) are more resistant to radiation-induced apoptosis and survive chemotherapy through increased expression of drug transporters. Finally, impaired functioning of apoptotic pathways has been described in these cells [9].

Thus, to be effective, cancer treatments should also target cancer stem-like cells, either by killing them or by forcing them to acquire a differentiated state more sensitive to conventional treatments [5, 10]. In this context, different strategies have been used to target such cells [11–13]. So far, however, most published data aim at finding molecules targeting proliferating tumor stem-like cells despite increasing evidence arguing in favor of the existence of relatively quiescent cancer stem-like cells within the tumor bulk *in vivo* [14]. Slowly proliferating cells with stem cell properties and tumor-initiation ability were identified in several solid tumors including ovarian, liver, breast cancer and melanoma [15–18]. In addition, a slow-cycling stem cell subpopulation from pancreatic adenocarcinoma has been shown to be endowed with increased tumorigenic and invasive potential as compared with faster-cycling cells from the same tumors [19]. More importantly, the quiescent state may contribute to the resistance of cancer stem-like cells to current chemotherapeutic agents. It was shown that leukemic stem cells survive in the dormant G0 phase of the cell cycle after chemotherapy and that relapses and metastases of breast cancer often occur after long intervals, suggesting an involvement of cells in a deep dormant phase [20–22]. Moreover, several studies have reported the resistance to conventional treatments of relatively quiescent cells from ovarian, breast and pancreatic tumors [14]. Thus, there is a great need to find new drugs that target both proliferating and quiescent tumor stem-like cells.

With the aim of tracking chemical compounds with the aforementioned properties, we screened the Prestwick Library, using patient derived human GSCs. The activity of the compounds was evaluated on both proliferating cells and on cells grown under conditions favoring their quiescence. Most hit compounds were active under both conditions and showed cytotoxicity towards control cell types including human primary astrocytes (HA cells) and non-cancer human fetal neural stem cells (f-NSCs). Interestingly, one drug, bisacodyl, showed high specificity towards quiescent GSCs. Subsequent structure-function relationship studies identified 4,4'-dihydroxydiphenyl-2-pyridyl-methane (DDPM), the metabolite of bisacodyl as the minimal pharmacophore carrying activity. Because of its specific activity profile, bisacodyl appears as a potential chemotherapeutic agent, able to target the particularly resistant quiescent cancer stem-like cells present within human tumors, that may be used as adjuvant in a multi-therapy approach.

## Materials and Methods

### Materials

Bisacodyl (4,4'-diacetoxydiphenyl-2-pyridyl-methane; CAS number: 603-50-9) and DDPM (4,4'-dihydroxydiphenyl-2-pyridyl-methane; CAS number: 603-41-8) also called BHPM (bis-(p-hydroxyphenyl)-pyridyl-2-methane) were purchased from Sigma-Aldrich.

### Ethics statement

The biomedical research was conducted according to the declaration of Helsinki, to the French laws and was approved by the institutional review board of Sainte Anne Hospital, Paris, France. Patients have given written informed consent. Isolation and characterization of neural stem cells from human fetal brain at embryonic day 50–55 (Carnegie stage 19–22) were performed under ethical approval from the University Paris-Descartes internal review board using tissue donated with written informed consent after elective termination of pregnancy.

### Cell culture

Glioblastoma (WHO grade IV glioma) stem-like cells (TG1, TG16 and OB1 GSCs) were derived from tumor samples of 3 patients (Sainte Anne Hospital, Paris, France), as previously described [23], and expanded as neurosphere cultures. In proliferating cultures, neurospheres were mechanically dissociated in single-cell suspensions twice a week. Quiescent cells were obtained by non-renewal of the medium for 9–16 days following cell seeding. Experimental procedures used to phenotypically and functionally characterize proliferating and quiescent GSCs are described in the phenotypic and functional characterization section of Materials and Methods.

Primary human astrocytes (HA cells) were expanded in AM Medium (from ScienCell Research Laboratories, Carlsbad California) according to the manufacturer's instructions.

Human embryonic kidney 293 cells (HEK 293 cells) were expanded in minimum essential medium with 2 mM L-glutamine, 100 IU/mL-100 μg/mL penicillin-streptomycin and 10% FBS.

Human fetal neural stem cells (f-NSCs) were isolated and cultured as previously described [24].

Human brain tumor cells U-87 MG (American Type Culture Collection, ATCC) were expanded in ATCC complete growth medium according to the manufacturer's instructions.

Master and working cell banks were established for all cell types. Cells were used at defined ranges of cell passages. Additional information concerning cell source, handling and resource sharing information is provided in S1 Table.

### Phenotypic and functional characterization of GSCs

#### Measurements of cell cycle, cell proliferative activity and cell viability

DNA synthesis activity of TG1 and OB1 GSCs at different conditions (1–16 days without medium renewal, 9 days without medium renewal followed by medium change at day 9 and day 13) was assessed with the Click-iT EdU (5-ethynyl-2'-deoxyuridine) Flow Cytometry Assay Kit from Invitrogen. Cell viability was measured using 7-AAD included in this kit.

For Ki-67 expression studies and cell cycle analysis using propidium iodide staining, cells were permeabilized and fixed in 70% ethanol at -20°C for 2h. They were then incubated with FITC-conjugated Ki-67 mouse anti-human antibody (Life technologies, MHKI6701) or FITC-conjugated mouse IgG1 isotype control (Life technologies, MG101) at room temperature for 30 min. After a treatment with 10 μg/mL RNase A and 20 μg/mL of propidium iodide for 30 min at room temperature, cells were analyzed on a FACSCalibur flow cytometer (BD Biosciences).

#### Expression of apoptosis and cell cycle related genes

Total RNA was isolated from 5–10 x 10^6^ TG1 or OB1 GSCs using the TRI Reagent (Euromedex, France) according to the manufacturer's instructions. RNeasy mini kit columns (QIAGEN) were used for further purification of the RNA samples. Cells were used in the following conditions: proliferating, quiescent (9 days without medium renewal) and proliferating after quiescence corresponding to quiescent cells (9 days without medium renewal) reintroduced into a proliferating medium for 1–4 days. NanoDrop ND-1000 (Labtech) was used for absorption spectra analysis and RNA purity assessment. Absorption ratios A260/A280 and A260/A230 were comprised between 1.8 and 2.1. RNA concentration was determined using the Qubit fluorometer and the Quant-it RNA Assay Kit from Invitrogen. RNA integrity was further evaluated with an Agilent 2100 Bioanalyzer and the RNA 6000 LabChip kit. Only RNA with a RNA Integrity Number (RIN) higher than 9 was processed (2100 expert software, Agilent Technologies). 1 μg of total RNA was reverse transcribed to single-stranded cDNA using the High Capacity cDNA Reverse Transcription kit (Applied Biosystems, Life Technologies). Real-time PCR analysis was performed with individual TaqMan gene expression assays in an ABI Prism 7000HT apparatus (Applied Biosystems, Life Technologies) using standard experimental conditions designed by the manufacturer. Individual assay IDs are as follows: p53: Hs 01034249-m1; BAX: Hs00180269-m1; p21: Hs 00355782-m1. Results were normalized to the 18S rRNA expression levels determined in all conditions. Results are shown as mean ± SD of two independent experiments.

#### Senescence evaluation

Senescence-associated β-galactosidase (SA-β-gal) activity was examined with Cellular Senescence Assay Kit (Merck Millipore, KAA002) according to the manufacturer’s protocol. Briefly, cells were fixed with paraformaldehyde-based Fixing Solution for 10 min at room temperature and then incubated with SA-β-gal Detection Solution at 37°C overnight before microscopy examination. U-87 MG cells treated with 100 μM TMZ for 5 days were used as a positive control.

#### Expression of stemness, pluripotency and differentiation markers

TaqMan QPCR Assays (Applied Biosystems, Life Technologies) were used for determination of stemness and differentiation marker mRNA expression levels as indicated in S1 Methods. Validation of gene expression levels for some genes (IFITM1: Hs00705137-s1, GBX2: Hs00230965-m1, NANOG: Hs04399610-g1) with individual gene expression assays (Applied Biosystems, Life Technologies) was performed as described in the expression of apoptosis and cell cycle related genes section of Materials and Methods. The percentage of proliferating and quiescent cells expressing the Nanog protein (antibody from R&D) was determined by FACS analysis (S1 Methods).

#### Expression of surface markers, clonal, *in vitro* differentiation and *in vivo* engraftment properties

Surface marker expression, clonal properties of both proliferating and quiescent cells, as well as their *in vitro* differentiation ability and their *in vivo* engraftment properties, were assessed according to S2, S3, S4 and S5 Methods, respectively.

### Primary and secondary chemical screens

The Prestwick Library (commercialized by Prestwick Chemical) used in this screen is composed of 1120 off-patent drugs (mostly FDA-approved) and some natural substances. Proliferating or quiescent TG1 GSCs were seeded (30 000 and 40 000 viable cells/well, respectively) into 96-well opaque bottom plates (Greiner) with the Biomek FX robot (Beckman Coulter). Each compound from the Prestwick Library was then added (final concentration: 50 μM; 1% DMSO). Each molecule was tested once. Negative control wells (12/96 per assay plate) contained cells treated with 1% DMSO (final concentration) and positive control wells (4/96 per assay plate) contained cells treated with the fungal toxin ophiobolin A (Sigma Aldrich) at 50 μM with 1% DMSO. Ophiobolin A is cytotoxic both to proliferating and quiescent GSCs. Relative ATP levels were measured 24 hours later using the CellTiter-Glo reagent (Promega) according to the manufacturer's instructions. Luminescence in each well was measured with the Victor3 plate reader (PerkinElmer).

Relative ATP levels in each well were determined by calculating the percentage of luminescent signal in the well with respect to the average signal measured in negative control wells. Compounds were considered as hits if relative ATP levels in the respective wells were less than 5% and/or if the corresponding luminescent signal was lower than the mean signal of negative control wells minus 5 times the standard deviation from this value.

Primary screen hits were further tested in duplicate, at two concentrations (50 and 5 μM) on proliferating and quiescent TG1 GSCs. Cell plating and treatment with compounds were as for the primary screen. Hit selection/confirmation criteria for quiescent cells were as described above. Due to a higher variability on proliferating cells’ assay plates, compounds were considered as hits if the corresponding luminescent signal was lower than the mean signal of negative control wells minus only 3 times the standard deviation from this value.

Screen reliability was evaluated by the Z' factor [25] for each assay plate. The median Z' factors were 0.615 and 0.68 for the primary and secondary screens, respectively. Results were taken into account only if Z'>0.5.

### Dose-response curves and EC_50_ determination

Hits were validated by performing dose-response curves on the viability of proliferating and quiescent TG1 cells under conditions similar (cell density, time of treatment) to those used for the primary and secondary screens (n = 3).

The EC_50_ value was determined for each compound by fitting the data points according to the following equation:

$$y=\frac{S_{max}+S_{min}*{\left(x*\frac{1}{EC_{50}}\right)}^{n}}{1+{\left(x*\frac{1}{EC_{50}}\right)}^{n}}$$

where **y** represents the expected response, **x** is the chemical compound concentration, **S**_**max**_ and **S**_**min**_ are the maximum and minimum responses recorded, respectively and n is the Hill coefficient. Curve fitting was performed using Microsoft Excel Solver component.

Similar dose response curves were performed using GSCs isolated from GBM of two other patients (TG16 and OB1 GSCs) grown under proliferating and quiescent conditions. Dose response curves were also performed using f-NSCs (100 000 cells/well), HEK 293 and HA cells (50 000 cells/well).

### Cell viability measurements

Proliferating and quiescent TG1 cells and HA cells were treated with bisacodyl (10 μM, 1% DMSO for TG1 cells; 50 μM, 1% DMSO for HA cells) for 24 hours. Cell mortality was evaluated by trypan blue staining (0.1% (v/v)). Control cells were incubated for 24 hours in their medium in the absence or presence of 1% DMSO.

### Evaluation of bisacodyl stability in cells' medium

Bisacodyl was dissolved in freshly prepared TG1 culture medium or quiescent TG1 conditioned culture medium at a final concentration of 10 μM in 1% DMSO. The solution was kept at room temperature. At given times (2 min, 2h, 4h, 6h and 24h), aliquots of 150 μL were taken and mixed with 150 μL of acetonitrile in order to precipitate proteins. After vortexing, the mixture was centrifuged at 15 000g for 10 min. Supernatants were analyzed by HPLC using a kinetex 2.6μ C18 100A (50x4.6 mm) column. Areas under the compound elution peaks were used for quantification. Reference solutions of bisacodyl or derivatives were used for calibration. Bisacodyl, its monoester derivative and the bi-phenolic form (DDPM) were eluted at 1.78 min, 1.56 min and 1.35 min, respectively.

### Synthesis of bisacodyl and bisacodyl derivatives

Commercially available bisacodyl and DDPM could also be synthesized according to our reported procedures (S6 Methods). General methods for bisacodyl and bisacodyl derivative synthesis and reaction schemes are given as supporting information (S6 Methods). HPLC-MS chromatograms of all the compounds with the exception of commercial ones are presented in S7 Methods.

### Dose-response curves and EC50 calculations for SAR studies

All chemical compounds synthesized were dissolved in DMSO to obtain 10 mM stock solutions.

Proliferating or quiescent TG1 cells were seeded in 50 μL of their respective media (30 000 and 40 000 viable cells/well, respectively) into 96-well opaque bottom plates (Greiner, Courtaboeuf, France). Compound treatment was performed by adding 50 μL of compound solutions (in the presence of 2% DMSO in culture medium). Negative control wells contained cells treated with 1% DMSO and positive control wells contained cells treated with the antihistaminic drug Terfenadine (50 μM) (BIO-TREND) which was identified during the screening process and shown to be cytotoxic to all the cell types used in this study. ATP levels were measured 24h later and EC_50_ values were determined as described above. All compounds were tested in triplicate in each experiment. Independent experiments were performed at least twice for almost all of the compounds.

## Results

### Quiescence of glioblastoma stem-like cells *in vitro*

**Fig 1:**
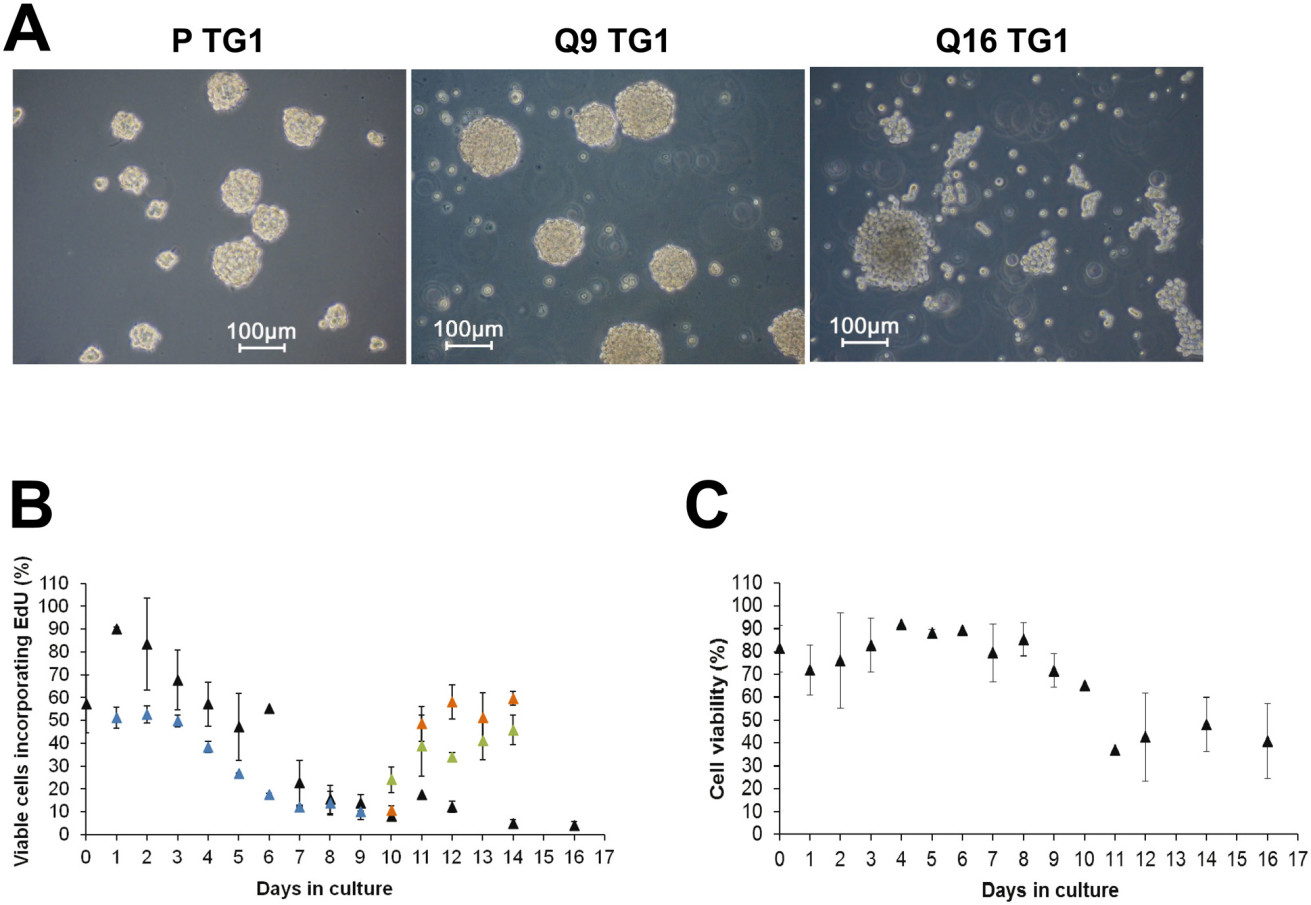
EdU incorporation and cell viability measurements on proliferating and quiescent TG1 and OB1 GSCs maintained *in vitro*. (A) In the absence of serum (NS34 culture medium), proliferating TG1 GSCs (P TG1) grow as neurospheres (left panel). Non-proliferating quiescent TG1 GSCs are generated *in vitro* by leaving cells without medium change for 9–16 days (middle panel: Q9 quiescent TG1 cells; right panel: Q16 quiescent TG1 cells). Scale bars, 100 μm. (B) EdU incorporation into TG1 (black triangles) and OB1 GSCs’ DNA (blue triangles) grown without medium renewal for 1–16 days or into cells grown without medium renewal for 9 days and then subjected to freshly prepared culture medium at days 9 and 13 (TG1 cells: orange triangles; OB1 cells: green triangles). Results are from at least two independent experiments. (C) Survival of TG1 cells grown in culture for 1–16 days without medium renewal. Survival was assessed using 7-AAD. Mean ± SD (n = 3).

**Fig 2:**
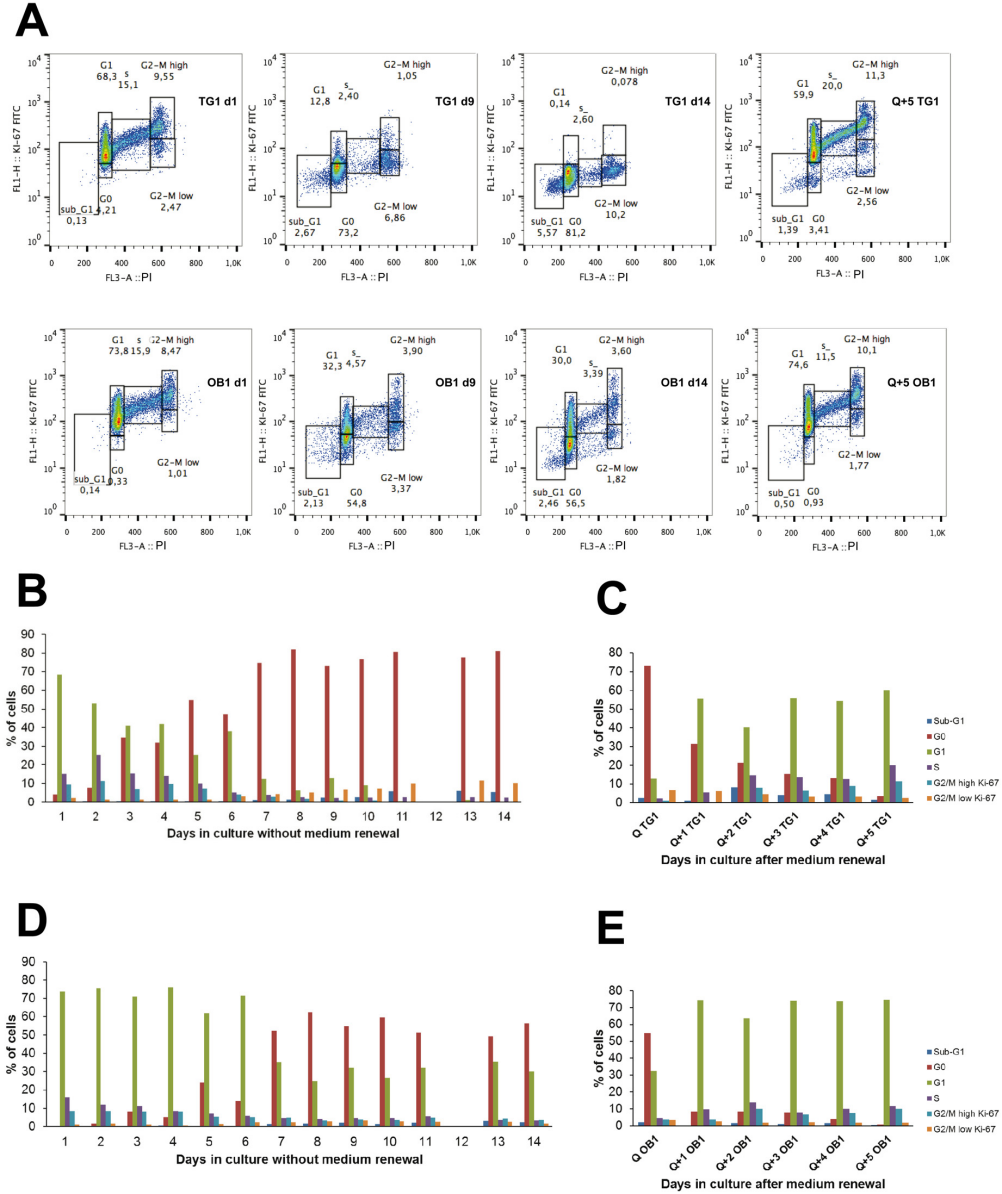
Cell cycle analysis and Ki-67 expression in TG1 and OB1 GSCs under different *in vitro* culture conditions. (A) TG1 (upper panel) and OB1 (lower panel) GSCs maintained in culture for 1, 9 and 14 days without medium renewal (d1, d9, d14, respectively) or cells obtained after 9 days without medium renewal and subjected to freshly prepared culture medium for 5 days (Q+5) were permeabilized and stained with anti-Ki-67 antibodies conjugated to FITC and propidium iodide (PI). (B-E) Histograms represent the percentage of TG1 (B, C) and OB1 (D, E) cells present in the G1, S, G2/M phases of the cell cycle as well as the percentage of quiescent cells in G0 and of cells showing DNA fragmentation (sub-G1). Cells in the G2/M phase were further distinguished as a function of the Ki-67 proliferation marker expression levels (G2/M high Ki-67 and G2/M low Ki-67). Analysis was performed on cells maintained for 1–14 days without medium renewal (B, D for TG1 and OB1 cells, respectively) or on cells left without medium renewal for 9 days (Q) and re-introduced into freshly prepared culture medium for 1 (Q+1), 2 (Q+2), 3 (Q+3), 4 (Q+4) or 5 (Q+5) days (C, E for TG1 and OB1 cells, respectively).

**Fig 3:**
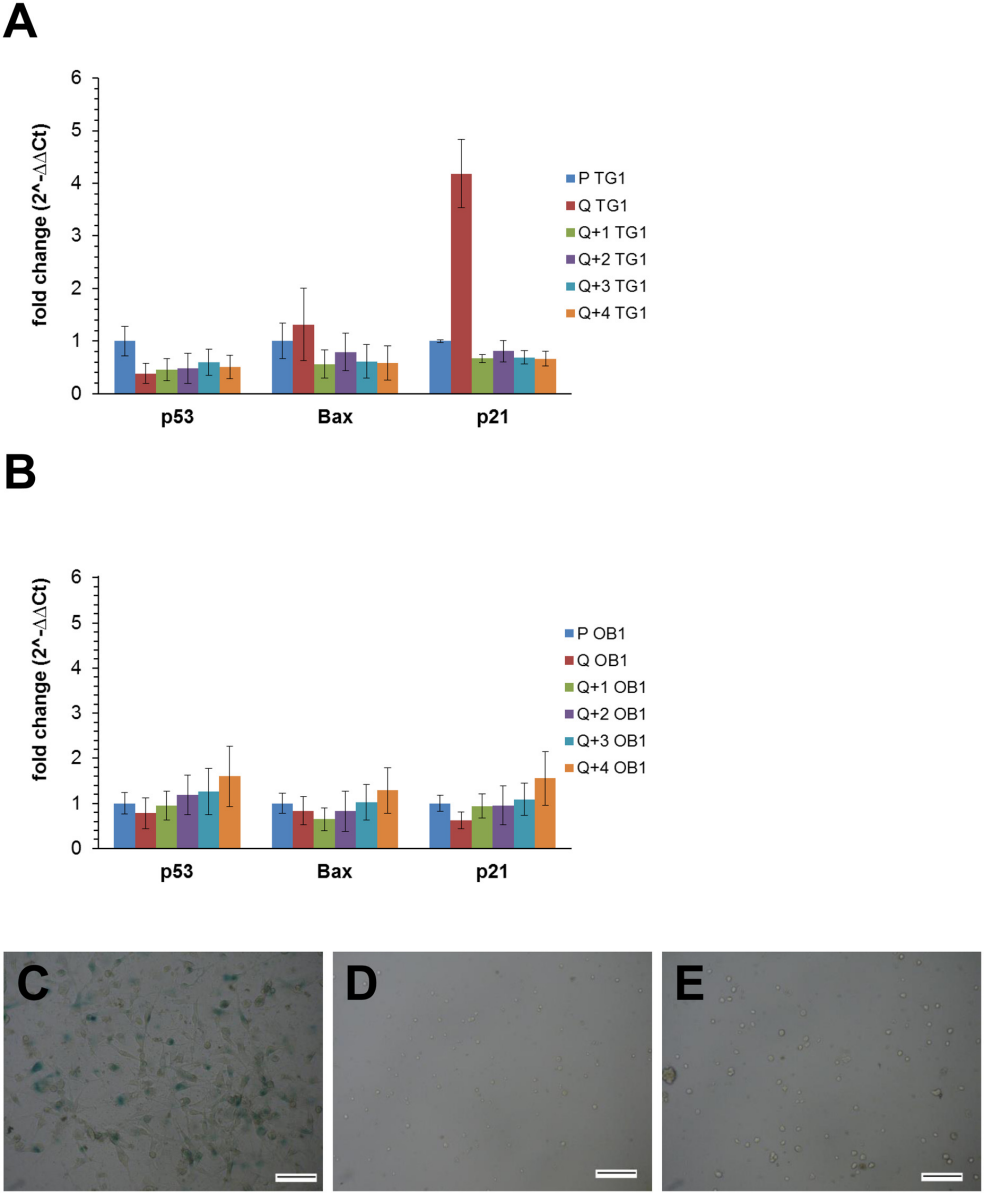
Expression of apoptosis and cell cycle related genes and absence of senescence related activity in TG1 and OB1 cells under different *in vitro* culture conditions. (A-B) Histograms representing mRNA expression levels of p53, Bax and p21 genes in proliferating (P) and quiescent (Q) TG1 (A) and OB1 (B) GSCs obtained after 9 days without medium renewal or quiescent cells after 9 days without medium renewal reintroduced in proliferating culture medium for 1–4 days (Q+1-Q+ 4). Results were expressed as fold change (2^∧-ΔΔCt^) taking proliferating TG1 (A) or OB1 (B) cells as calibrator. 18S rRNA was used as housekeeping gene. Data were from two independent experiments, each performed in duplicate. (C-E) Senescence-related β-galactosidase activity measurements in U-87 MG cells treated with TMZ (100 μM) for 5 days (C; positive control) and in quiescent TG1 and OB1 GSCs obtained after 9 days without medium renewal (D, E, respectively). Scale bars: 100 μm.

Proliferating TG1 GSCs (Fig 1A, left panel) were previously selected and expanded in culture through the neurosphere assay. These cells were extensively characterized and showed long-term self-renewal, clonal properties and ability to initiate tumor formation *in vivo* thus fulfilling the criteria of tumor stem-like cells [23]. To achieve quiescence of TG1 GSCs *in vitro*, proliferating cells were seeded (day 0) and left without medium renewal. EdU incorporation and 7-AAD staining were used to assess cell proliferation and viability. GSCs maintained in culture up to 16 days without medium renewal were morphologically similar to their proliferating counterparts. They form neurospheres (Fig 1A) which at day 9 were similar to those formed under proliferating conditions and slightly looser at day 16. At day 0, just after cell passaging, 50–60% of the cells incorporated EdU (Fig 1B). The percentage of cells going through the S phase increased significantly at days 1 and 2 and then returned to initial levels by day 4. A marked decrease was observed between days 4 and 8. The low level of DNA synthesis activity measured at day 8 remained almost similar between days 8 and 16. TG1 GSC viability measured at the same time points was not significantly decreased until day 9, whereas the number of cells incorporating EdU was already markedly affected at this time point (Fig 1B and 1C). At later time points, the number of viable cells decreased and then remained stable between days 11 and 16 (Fig 1C). Similar EdU incorporation profiles were obtained for OB1 and TG16 GSCs although the percentage of OB1 cells incorporating EdU was generally lower than the one observed for TG1 cells (Fig 1B for OB1 cells). To evaluate if quiescence was reversible, TG1 and OB1 cells left for 9 days without medium renewal (and called thereafter Q9 cells) were reintroduced into freshly prepared NS34 culture medium. Medium change was preformed twice, at day 9 and at day 13. As indicated in Fig 1B, medium renewal led to an increase of the percentage of TG1 and OB1 GSCs incorporating EdU suggesting that cells are able to re-enter the S phase, *i*.*e*. that the non-dividing state obtained by the absence of medium renewal is reversible. Cell cycle exit and reentrance of TG1 and OB1 GSCs was confirmed by cell cycle analysis using propidium iodide staining coupled to the study of modifications of Ki-67 protein levels, known to be high in dividing cells and low in non-cycling cells. As shown in Fig 2A, 2B and 2D, absence of medium renewal led to a significant increase in the number of TG1 and OB1 cells in the G0 phase with a maximum obtained after 8 days in non-renewed medium. From days 8 to 14, the percentage of cells in the G0 phase did not vary significantly (Fig 2B and 2D). Concomitant with the increase of the percentage of cells in the G0 phase, there is a decrease in the number of dividing cells (cells expressing Ki-67 in the G1, S and G2/M phases of the cell cycle) (Fig 2A upper and lower panels and Fig 2B and 2D). When Q9 quiescent cells were submitted to fresh medium, both TG1 and OB1 GSCs were able to re-enter the cell cycle as shown by the increase of the percentage of cells in G1, S, G2/M and the decrease of the number of cells in phase G0 (Fig 2A upper and lower panels and Fig 2C and 2E). Interestingly, the mRNA levels of p21, a cyclin-dependent kinase inhibitor, reported to control both entry into quiescence and maintenance of the quiescent state [26] and whose expression may be controlled by p53, were significantly higher (4 fold) in quiescent TG1 GSCs after 9 days without medium renewal compared to their proliferating counterparts. The expression of this gene decreased when cells were placed back in proliferating conditions (Fig 3A). No significant changes in the expression level of this gene were observed between proliferating and quiescent OB1 GSCs (Fig 3B) pointing to differences between these two GSC types. Of note, the percentage of cells showing DNA fragmentation (cells in sub-G1) did not significantly vary from day 1 to day 14 without medium change in TG1 and OB1 GSCs (Fig 2A upper and lower panels and Fig 2B and 2D). mRNA expression levels of p53, a known apoptosis regulator, showed a slight decrease (c.a. 50%) in Q9 quiescent TG1 GSCs which remained unchanged when these cells were reintroduced in proliferating conditions for 1–4 days (Fig 3A). No significant variations were observed in p53 expression levels in OB1 GSCs under similar conditions (Fig 3B). The mRNA levels of Bax, a mediator of mitochondrial apoptosis whose expression is controlled by p53, were also similar in proliferating *versus* Q9 quiescent TG1 and OB1 GSCs (Fig 3A and 3B). These data reinforce the previous results and suggest that cells maintained *in vitro* without medium renewal for several days did not undergo massive apoptosis. In addition, the absence of β-galactosidase positive TG1 or OB1 GSCs after 9 days without medium renewal suggested that the cells obtained under these conditions were not senescent (Fig 3D and 3E). Altogether, these data strongly support the conclusion that quiescent and viable non-dividing GSCs may be obtained *in vitro* after several days without medium renewal.

**Fig 4:**
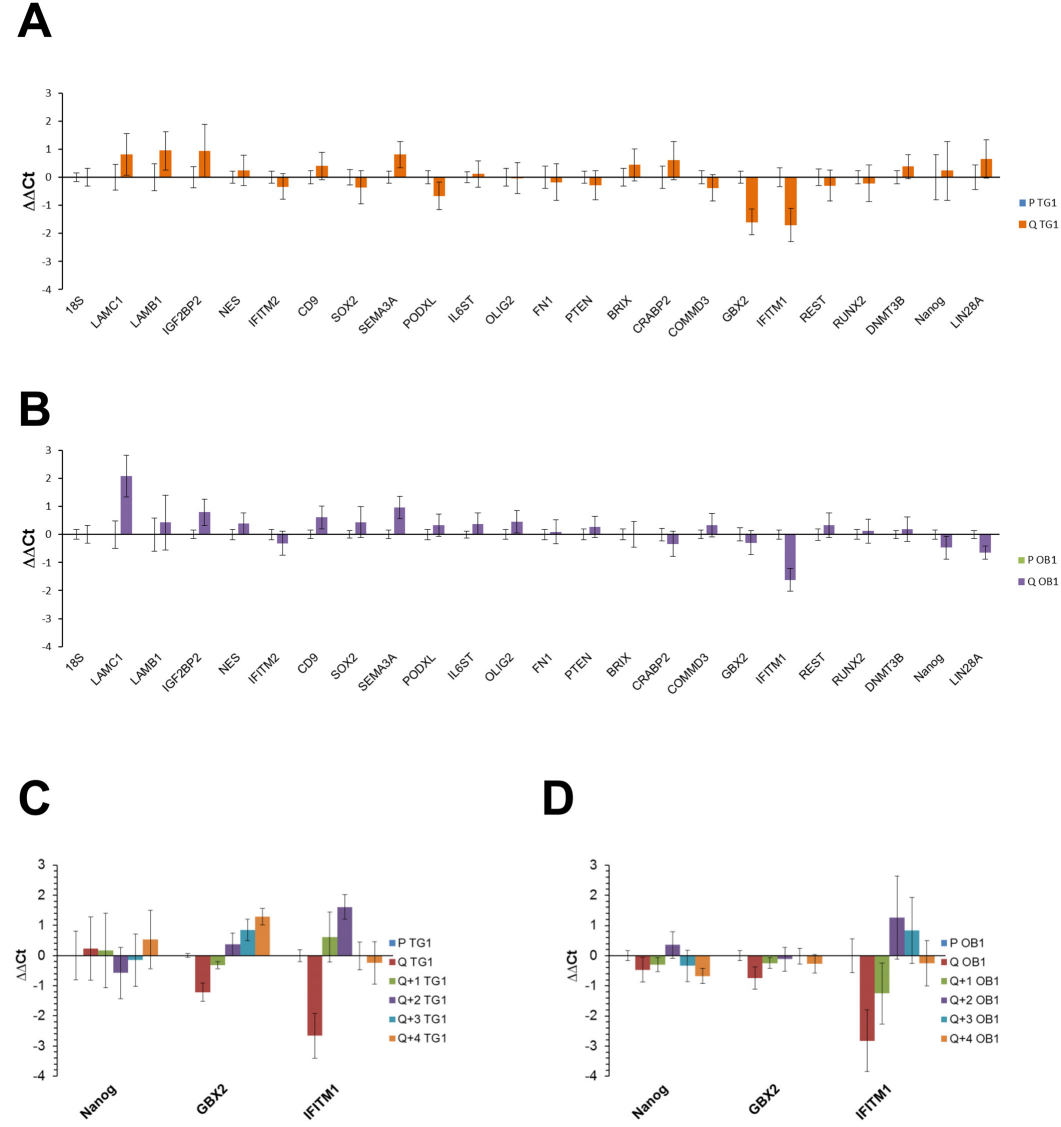
Expression of stemness, pluripotency or differentiation related genes in TG1 and OB1 cells under different *in vitro* culture conditions. (A-B) Histograms representing mRNA expression levels of stemness, pluripotency or differentiation associated genes included in the TaqMan Human Stem Cell Pluripotency Array from Life Technologies in proliferating (P) and quiescent (Q) (9 days without medium renewal) TG1 (A) and OB1 (B) GSCs. Results were normalized to the 18S rRNA levels and expressed as ΔΔCt taking proliferating TG1 (A) and OB1 (B) GSCs as calibrator samples. Data are from three independent experiments, each performed in duplicate. (C-D) Histograms representing mRNA expression levels of Nanog, GBX2 and IFITM1 genes in proliferating (P) and quiescent (Q) TG1 (C) and OB1 (D) GSCs obtained after 9 days without medium renewal or quiescent cells after 9 days without medium renewal reintroduced in proliferating culture medium for 1–4 days (Q+1-Q+4). Results are expressed as in A-B. Data are from two independent experiments, each performed in duplicate.

To determine whether quiescent culture conditions modify the stem-like phenotype of GSCs by inducing cell differentiation, expression of 90 well-defined genes validated as pluripotency or differentiation markers was analyzed in TG1 and OB1 GSCs grown under proliferating or quiescent conditions using TaqMan Human Stem Cell Pluripotency Arrays. Each array contains 7 genes expressed in undifferentiated cells or involved in maintenance of pluripotency, 31 genes correlated with stemness and 52 differentiation markers (see list in S2 Table). 18S rRNA, which did not show any expression change under the experimental conditions tested, was used as housekeeping gene. Only genes with ΔCt (gene cycle threshold (Ct) minus 18s rRNA cycle threshold) values ≤ 21 in at least one of the conditions tested were considered. Genes with ΔCt values > 21 corresponded to genes with low expression and poor signal to noise ratios. 23 genes were considered to be significantly expressed. As shown in Fig 4A and 4B, most of these genes (but GBX2, IFITM1 and LAMC1 (only in OB1 cells)) did not show a major change in their expression between proliferating and quiescent states. Namely, no significant changes in gene expression were observed for the pluripotency markers Sox2 and Nanog (confirmed in individual TaqMan assays for Nanog, see Fig 4C and 4D). Nanog expression was also evaluated at the protein level using FACS. More than 85% of the cells expressed this marker. This percentage remained similar between proliferating and quiescent cells (85% *vs* 89%, respectively, for TG1 GSCs; 88% *vs* 86% respectively, for OB1 GSCs). In addition, nuclear localization of Nanog was confirmed by immunocytochemistry (data not shown). Nanog expression also remained stable when quiescent cells re-enter the cell cycle (Fig 4C and 4D). The expression of GBX2 and IFITM1, two other stemness markers which presented some variation between proliferative and quiescent states on the arrays, was further analyzed in individual TaqMan assays. As shown in Fig 4C and 4D, quiescence induced a slight increase in GBX2 mRNA levels in TG1 GSCs (ΔΔCt: -1.22 +/- 0.3; fold change: 2.32 +/- 0.48) and OB1 cells (ΔΔCt: -0.75 +/- 0.37; fold change: 1.68 +/- 0.43). These variations were reversed when quiescent cells re-entered the cell cycle (Fig 4C and 4D). The GBX2 gene product has previously been shown to promote reprogramming and maintenance of the pluripotent state of mouse embryonic stem-cells downstream of a LIF/Stat3-dependent pathway [27]. A greater change in expression was observed for IFITM1 when TG1 and OB1 cells became quiescent (ΔΔCt: -2.66 +/- 0.75; fold change: 6.32 +/- 3.26 and ΔΔCt: -2.82 +/- 1.02; fold change: 7.06 +/- 4.99, respectively) (Fig 4C and 4D). When cells were reintroduced into proliferating conditions (following medium renewal), IFITM1 expression decreased rapidly to reach levels similar to those observed in proliferating GSCs (Fig 4C and 4D). IFITM1 was previously shown to regulate proliferation either negatively or positively depending on the cell model [28, 29]. Our data are in favor of an anti-proliferative effect of IFITM1 since its expression increases in quiescent cells. Finally, a slight decrease in the expression of LAMC1, an early differentiation marker, was detected but only in quiescent OB1 cells. On the overall, quiescence did not appear to cause major changes in the stemness of GSCs.

To further characterize quiescent TG1 and OB1 GSCs, we examined various properties previously described for these cells in proliferating conditions, namely the expression of cell surface markers, their clonal and differentiation properties as well as their engraftment ability after orthotopic injection in mouse brain. Concerning cell surface markers, quiescent GSCs expressed CXCR4 and CD56 and were CD133 negative, similarly to their proliferating counterparts (S1A–S1C Fig) [23, 30, 31]. In addition, quiescence did not alter TG1 and OB1 GSCs’ clonal properties since isolated quiescent cells, when placed back into fresh culture medium, formed new neurospheres (S2A and S2B Fig). Moreover, as their proliferating counterparts [23], quiescent cells kept their ability to differentiate when exposed to serum containing medium. Results for differentiation of both proliferating and quiescent TG1 and OB1 GSCs are presented in S3 Fig. Serum addition led to cell adherence and morphological changes reflected by flattening and acquisition of a fusiform shape. These morphological changes were accompanied by a decrease in the expression of the stemness-related gene Sonic Hedgehog (SHH) in proliferating and quiescent TG1 GSCs and in proliferating OB1 cells (S3B and S3D Fig). Concomitantly, a marked increase in the expression of the astrocytic marker glial fibrillary acid protein (GFAP) was observed in proliferating TG1 and OB1 cells and in quiescent TG1 GSCs (S3C and S3E Fig). A significant change in the expression of the neuronal marker β3 tubulin (TUBB3) was only observed when quiescent cells (TG1 or OB1) were subjected to differentiation (S3B and S3D Fig). Altogether these data suggest that quiescent GSCs obtained *in vitro* maintain their differentiation ability when subjected to serum supplemented medium and point to a preferential differentiation (under the conditions used) towards an astrocytic phenotype.

The last point investigated in this study concerned GSCs’ *in vivo* engraftment ability. This property was reported previously for TG1 and OB1 GSCs [23, 30]. Results presented in S4 Fig suggest that intracerebral engraftment was not altered when quiescent GSCs were injected into immune-deficient animal brains. Indeed, eight weeks after orthotopical injection, human cells were present in mouse brains, with no differences in cellularity whether injected cells were proliferating or quiescent (S4A and S4B Fig). In addition, in both cases, Ki-67 positive cells were detected, suggesting that the quiescent phenotype may be reversed *in vivo* (S4C and S4D Fig).

Altogether, the results presented suggest that i) quiescence can be obtained *in vitro* for GSCs, ii) quiescent GSCs retain the stem, clonal, differentiation and *in vivo* engraftment properties of their proliferative counterparts. GSCs were thus designated as quiescent after 9 to 16 days in culture without medium renewal.

### Prestwick Library screening of proliferating and quiescent GSCs

**Fig 5:**
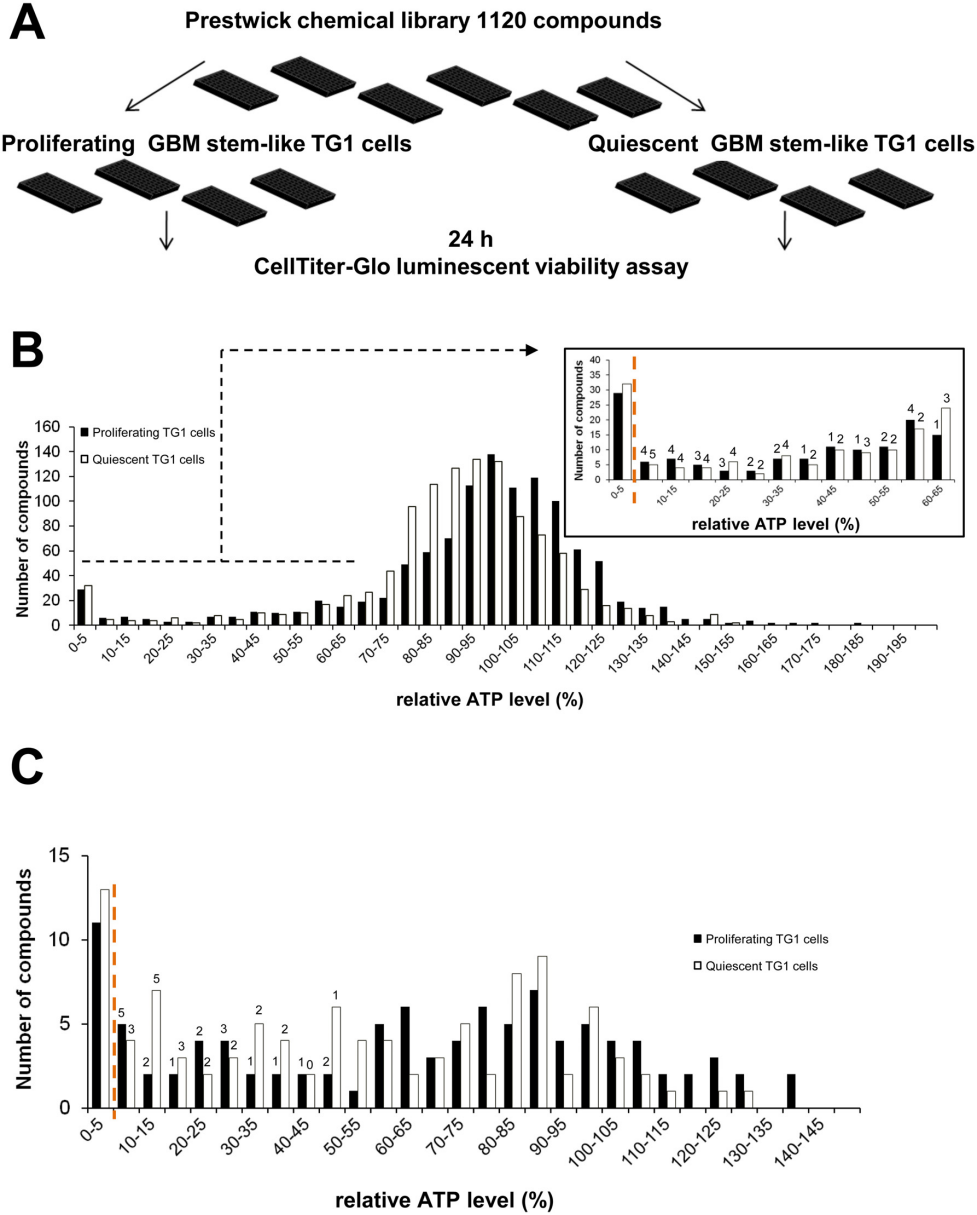
Prestwick Library screen on GSCs using the ATP-Glo cell based assay. (A) Schematic representation of the assay design and protocol. GBM: glioblastoma. (B) Results of the primary screen are represented as histograms of the relative ATP signal levels obtained for each compound screened against proliferating (black bars) and quiescent (open bars) TG1 cells. Molecules producing ATP levels that exceeded 200% of the control levels are not shown. Zoom in of results for compounds with an ATP level below 65% of the control levels is provided. A compound was considered as a hit either if it reduced relative ATP levels to less than 5% of control levels (compounds on the left of the orange dotted line) or if the corresponding luminescent signal was lower than the mean signal of negative control wells minus 5 times the standard deviation from this value (number of molecules indicated on the top of each bar). (C) 86 compounds reducing relative ATP levels and 16 molecules increasing relative ATP levels were tested in a secondary screen at concentrations of 5 and 50 μM. Results of the secondary screen performed at 50 μM are shown following the same representation as in (B).

The Prestwick Library was screened in order to find molecules with cytotoxic activity on proliferating and /or quiescent GSCs (Fig 5A). In the primary screen (Fig 5B), about 5% of the compounds tested significantly reduced the relative ATP level of proliferating or quiescent TG1 cells. Of the 1120 compounds, 57 were active on proliferating cells and 69 on quiescent GSCs, with 40 compounds exhibiting similar effects on both cell types. Seventeen compounds triggered an increase in ATP levels.

To confirm hits from the primary screen, a secondary screen was performed on 86 compounds that reduced relative ATP levels and 16 molecules that increased them. The secondary screen confirmed the activity of approximately 50% of the compounds that lowered ATP levels (29/57 compounds for proliferating TG1 GSCs and 33/69 for cells grown under quiescent conditions) (Fig 5C). Twenty-three of the confirmed hits were active on both proliferating and quiescent TG1 GSCs. None of the 16 compounds tested for their potential to increase cell ATP levels was confirmed. Results of primary and secondary screens are summarized in S3 Table.

Dose-response curves were generated using both proliferating and quiescent TG1 GSCs for 20 out of 39 active compounds selected in the secondary screen (list in Table 1). Compounds excluded were antibacterial, antifungal, anti-parasitic agents or molecules endowed with known detergent activity. All those excluded compounds showed activity on both proliferating and quiescent cells. Preliminary experiments were performed on the 20 selected compounds to verify that measured luminescence changes were related to an effect of the tested molecule on cell ATP levels and not to an interference with the readout setup. Analysis of the different dose-response curves allowed classifying the compounds into three groups, according to their EC_50_ values for TG1 GSCs in either their proliferative or quiescent state (Table 1). Structures and smile codes of the twenty hit compounds are represented in S4 Table.

**Table 1:** EC50 values of Prestwick Library hit compounds on the different cell types tested.

ATC	Compound name	Therapeutic/	P TG1	Q TG1	P TG16	Q TG16	P OB1	Q OB1	Astrocytes	f-NSC	HEK 293
classification		pharmacological class	GSCs	GSCs	GSCs	GSCs	GSCs	GSCs	(HA cells)		
			EC_50_ (μM)	EC_50_ (μM)	EC_50_ (μM)	EC_50_ (μM)	EC_50_ (μM)	EC_50_ (μM)	EC_50_ (μM)	EC_50_ (μM)	EC_50_ (μM)
**Group 1**											
C01BD01	Amiodarone hydrochloride	Cardiovascular system/Antiarrhythmic/Adrenergic blocker	10±3	10±3	11±1	14±5	12±1	19±1	22±7	10	28±4
C01DX02	Prenylamine lactate	Vasodilatator	22±3	27±3	32±2	42±8	30±7	49±4	30±2	9±2	25±6
C04AX19	Suloctidil	Vasodilatator peripheral/Hypolipidemic	11±1	13±1	13.5±0.1	14±6	15±2	20±4	11±1	5±1	10±1
C08CA02	Felodipine	Cardiovascular system/Antihypertensive/Calcium channel blocker	38±1	30±3	39±4	33±3	43±5	41±6	36±6	59±12	55±2
C08EA01	Fendiline hydrochloride	Cardiovascular system/Antiarrhythmic/Antianginal/L-calcium channel blocker	22±1	32±1	42±1	45±16	40±13	48±9	42±8	11±1	34±9
C08EX02	Perhexilin maleate	Cardiovascular/Vasodilatator	10±1	14±3	11±1	21±4	12±1	22±4	12±2	3±1	12±1
G03DC03	Lynestrenol	Progestogen	36±2	43±8	32±2	28±6	26±5	56±10	20±1	23±5	57±8
KEGG Brite C09154	Ellipticine	Anticancer	13±1	19±5	13±1	56±10	8±1	30±9	5±1	7±2	13±2
L02BA01	Tamoxifen citrate	Anticancer	14±2	20±1	20±1	20±3	28±3	29±3	15±4	14±9	30±10
N06AB06	Sertraline	Antidepressant/Psychoanaleptic	19±1	39±1	31±1	44±9	32±11	45±2	24±1	10±2	23±1
P02CF01	Ivermectin	Antiparasitic/Anthelmintic/Acaricid	3±1	4±1	1.5±0.2	6±2	3±1	7±2	3±1	4±1	13±1
KEGG C08921	Beta-Escin	Peripheral vascular disorders/Antioedematous/	17±3	12±3	6±1	8±1	12±1	6±1	7±1	nd	31±1
		Antiinflammatory/Glucocorticoid like?									
**Group 2**											
N05AF05	Zuclopenthixol hydrochloride	Antipsychotic	20±3	52±3	29±5	48±18	27±9	> 100	12±1	13±1	29±1
R06AX11	Astemizole	Antihistaminic	12±2	28±3	17±2	58±9	13±3	43±16	19±4	6.3±0.2	21±9
R06AX12	Terfenadine	Antihistaminic	8±1	17±1	9±1	24±6	10±2	22±4	9±2	4±1	8±1
KEGG Brite CPD:C07609	Parthenolide	Antiinflammatory/Antisecretory/Spasmolytic	15±2	48±6	10±2	nd	18±4	35±11	59±4	8.4±0.2	13±5
**Group 3**											
A06AB02	Bisacodyl	Cathartic laxative	> 100	1.07±0.04	> 100	1.3±0.1	> 100	0.5±0.1	0.6+/-0.3*	> 100	> 100
C03CC01	Ethacrynic acid	Diuretic	81±11	41±11	> 100	12±2	60±20	24±3	> 100	21±11	> 100
KEGG c01514	Luteolin	Expectorant	> 100	20±2	> 100	34±4	> 100	18±4	> 100	70±36	> 100
G03DA04	Progesterone	Progestogen	> 100	25±11	75±6	25±9	89±29	23±11	> 100	> 100	36±10

Screening of the Prestwick Library on TG1 human glioblastoma stem-like cells (GSCs) unveiled 20 molecules decreasing the relative ATP level in these cells. Dose response curves were performed with the 20 molecules using ATP Glo assay as readout. Molecules were tested on 3 different types of proliferating (P) and quiescent (Q) GSCs (TG1, TG16, OB1), non-cancer neural cells including human fetal neural stem cells (f-NSC) and primary astrocytes (HA cells) as well as HEK 293 cells, a non-cancer and non-neural cell line. Hits are classified into 3 groups according to their differential activity or not on proliferative and quiescent TG1 GSCs. In each group, molecules were clustered according to their ATC (Anatomical, Therapeutic, Clinical) classification. nd: not determined.

*: the maximum effect observed for this compound on HA cells was a reduction of only 30% of ATP levels which was not due to cell death.

**Fig 6:**
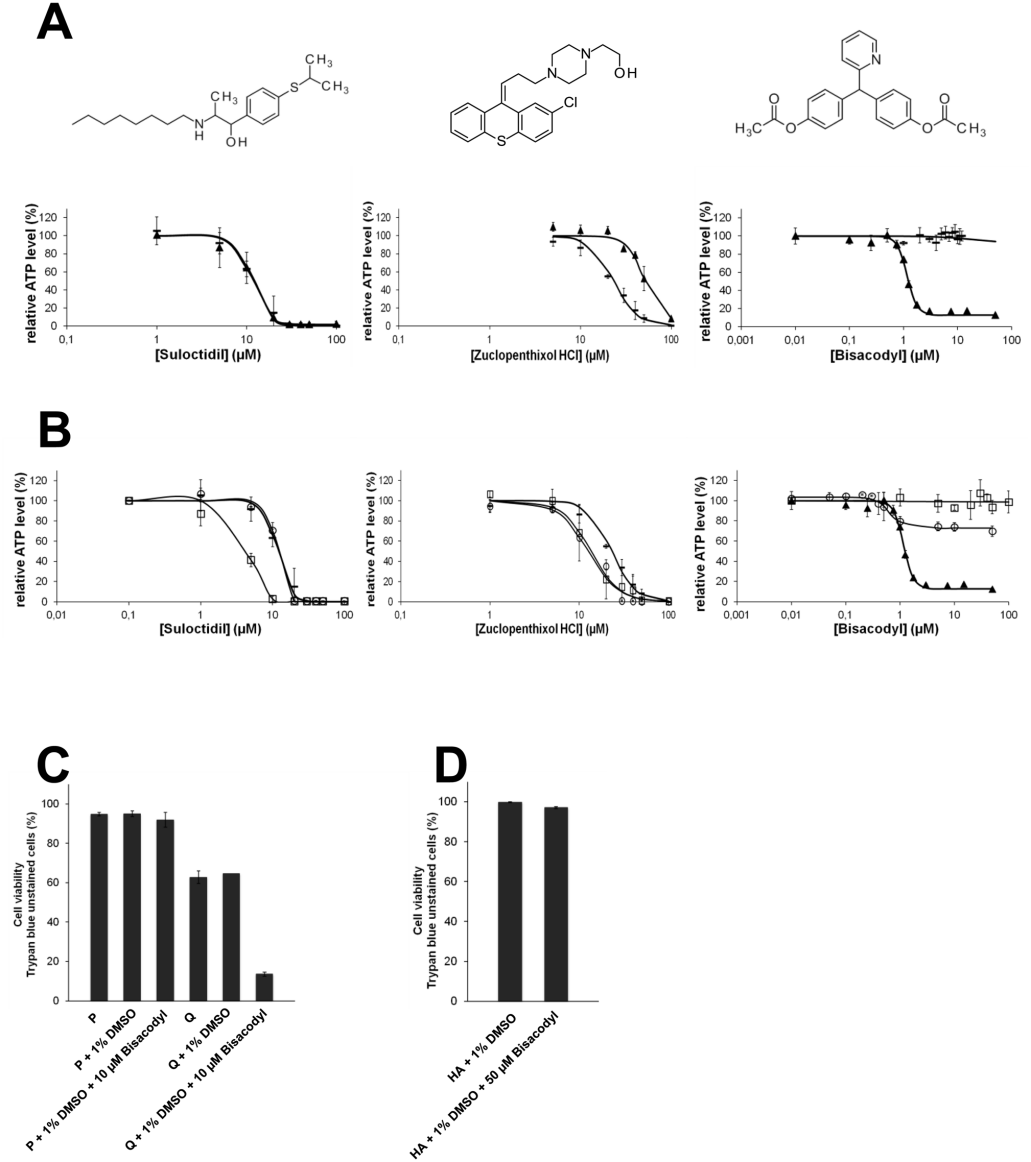
Dose-response curves for compounds exhibiting the strongest cytotoxic effect on GSCs. (A) Chemical structures of selected compounds and dose-response curves of suloctidil (left), zuclopenthixol HCl (middle) and bisacodyl (right) with representative activity profiles on proliferating (─) and quiescent (▲) TG1 GSCs. The fitted sigmoidal logistic curve to ATP-Glo cell survival assay readouts is shown on each plot (n = 3). (B) Dose-response curves of suloctidil (left), zuclopenthixol HCl (middle) and bisacodyl (right) on proliferating TG1 cells (─), quiescent TG1 cells (▲), human primary astrocytes (О) and human fetal neural stem cells (◻). Curves were fitted as in A (n = 3). (C-D) Cell viability measurements (trypan blue staining) on proliferating (P) or quiescent (Q) TG1 cells (C) and HA cells (D) treated with bisacodyl (compound **1** in Fig 9).

Representative results of the activity profiles of each group are shown in Fig 6A. Suloctidil, a vasodilator, is representative of a group of compounds (group 1) with cytotoxic activity towards both proliferating and quiescent TG1 GSCs (Fig 6A, left panel). For this group, compound EC_50_ on proliferating GSCs was less than two-fold higher or lower compared to the value observed on their quiescent counterparts. Twelve compounds belong to this family (Table 1). The antipsychotic zuclopenthixol hydrochoride (Fig 6A, middle panel) is representative of a group of 4 molecules which exhibited higher activity towards proliferative cells (group 2). In this group, compound EC_50_ was more than two fold lower for proliferative TG1 GSCs compared to quiescent cells. Group 3, represented by the laxative bisacodyl (Fig 6A, right panel) unveils molecules with selectivity for quiescent TG1 GSCs. In this group, EC_50_ of a given compound was at least two fold lower for quiescent TG1 GSCs compared to proliferative cells. Bisacodyl was the molecule with the lowest EC_50_ and which appeared with outstanding specificity for quiescent TG1 GSCs (Fig 6A right panel and Table 1).

**Fig 7:**
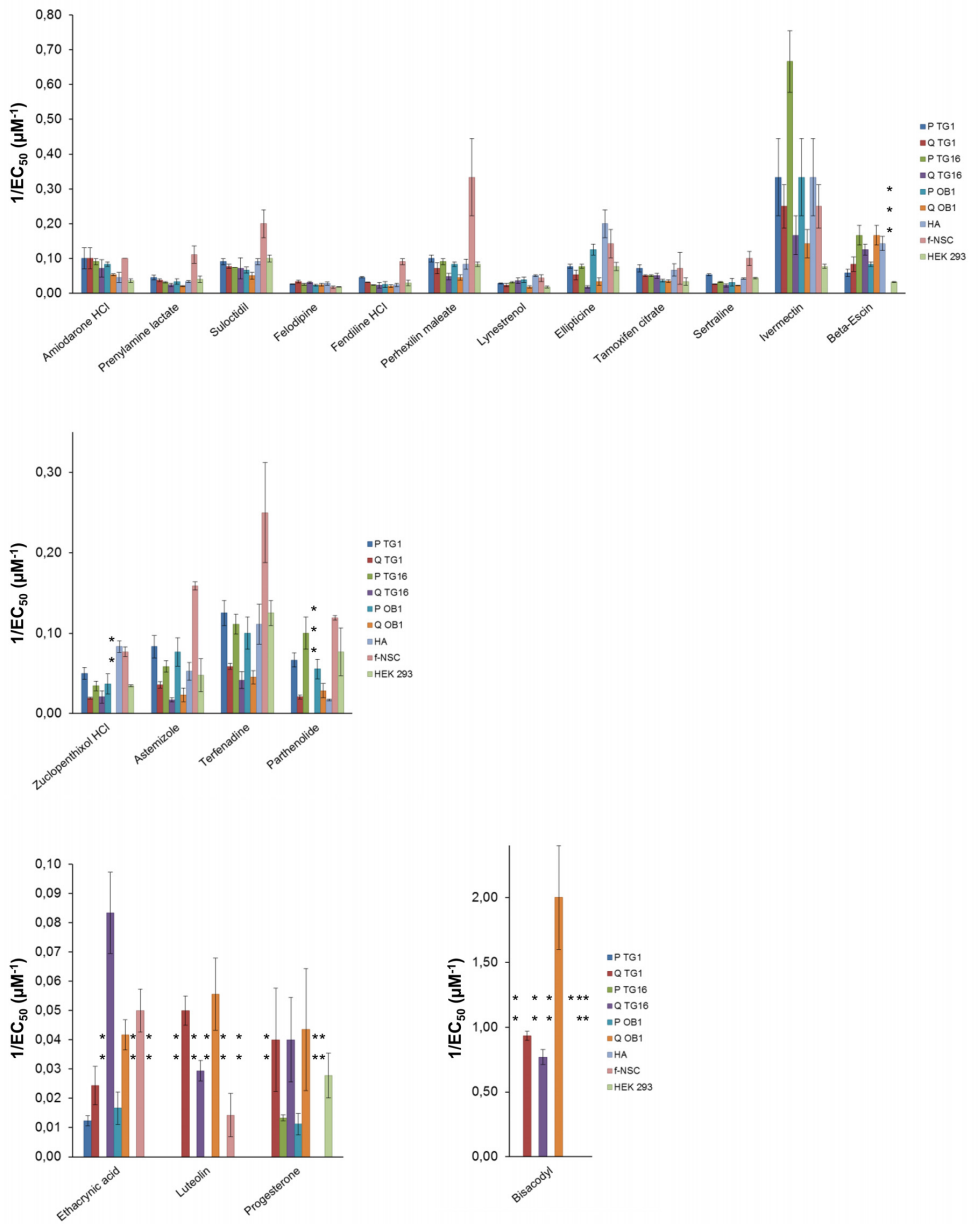
Activity of Prestwick Library hit compounds on GSCs and control cell types. Graphical presentation of the activity of the high-throughput screen hit compounds (expressed as 1/EC_50_) on proliferating (P) and quiescent (Q) GSCs derived from three patients (TG1, TG16 and OB1), human primary astrocytes (HA cells), human fetal neural stem cells (f-NSC) and a human embryonic kidney cell line (HEK 293). Upper, middle and low panels: group 1, group 2 and group 3 molecules as defined in Table 1, respectively. EC_50_ values correspond to mean values calculated from fitted dose-response curves to ATP-Glo assay readings of three independent experiments (n = 3). *: Given that the maximum effect of bisacodyl on HA cells is a 30% reduction of ATP levels which is not due to cell death (see Fig 6D), the corresponding EC_50_ value was not taken into account. **: EC_50_ values observed >100 μM. ***: EC_50_ values not determined.

The activity of the 20 molecules was further tested on stem-like cells derived from two other distinct human glioblastomas (TG16 and OB1 GSCs). As for TG1 GSCs, dose response curves were performed on cells grown under proliferating and quiescent conditions. Activity profiles and EC_50_ values obtained were similar to those observed for TG1 GSCs (Table 1 and Fig 7).

To determine the specificity of the selected compound towards GSCs, dose-response curves were performed on the non-cancerous neural cells f-NSCs, and HA cells as well as on to the non-neural cell line HEK 293. Most of the 20 compounds, namely those belonging to groups 1 and 2 (Table 1), showed cytotoxicity, after the 24 hours of the test, towards all the cell types tested, whether they were neural or not, cancerous or not (Table 1 and Fig 7). Compounds of group 3 which showed higher activity on quiescent GCSs compared to proliferative ones were mostly inactive or showed reduced activity on the other cell types. One molecule, bisacodyl, stood out from the rest by showing a specific activity on GSCs grown under quiescent conditions, with a low EC_50_ ~1 μM (Figs 6A and 7; Table 1). For quiescent TG1 GSCs, trypan blue and 7-AAD staining indicated that the ATP level decrease induced by bisacodyl resulted from cell death (Fig 6C). In addition, bisacodyl showed no activity on f-NSCs and HEK 293 cells (Table 1, Figs 6B and 7). A decrease in the relative ATP level could be observed for HA cells (primary astrocytes) (Fig 6B). However, using trypan blue exclusion, we showed that bisacodyl did not alter HA viability (Fig 6D), suggesting that this compound might induce metabolic changes in these cells.

Altogether, these data, pointed to bisacodyl as a highly potent and selective inhibitor of quiescent GSC survival. This property drew our attention and triggered further investigation of the molecule.

### Bisacodyl acts on GSCs via DDPM, its active metabolite

**Fig 8:**
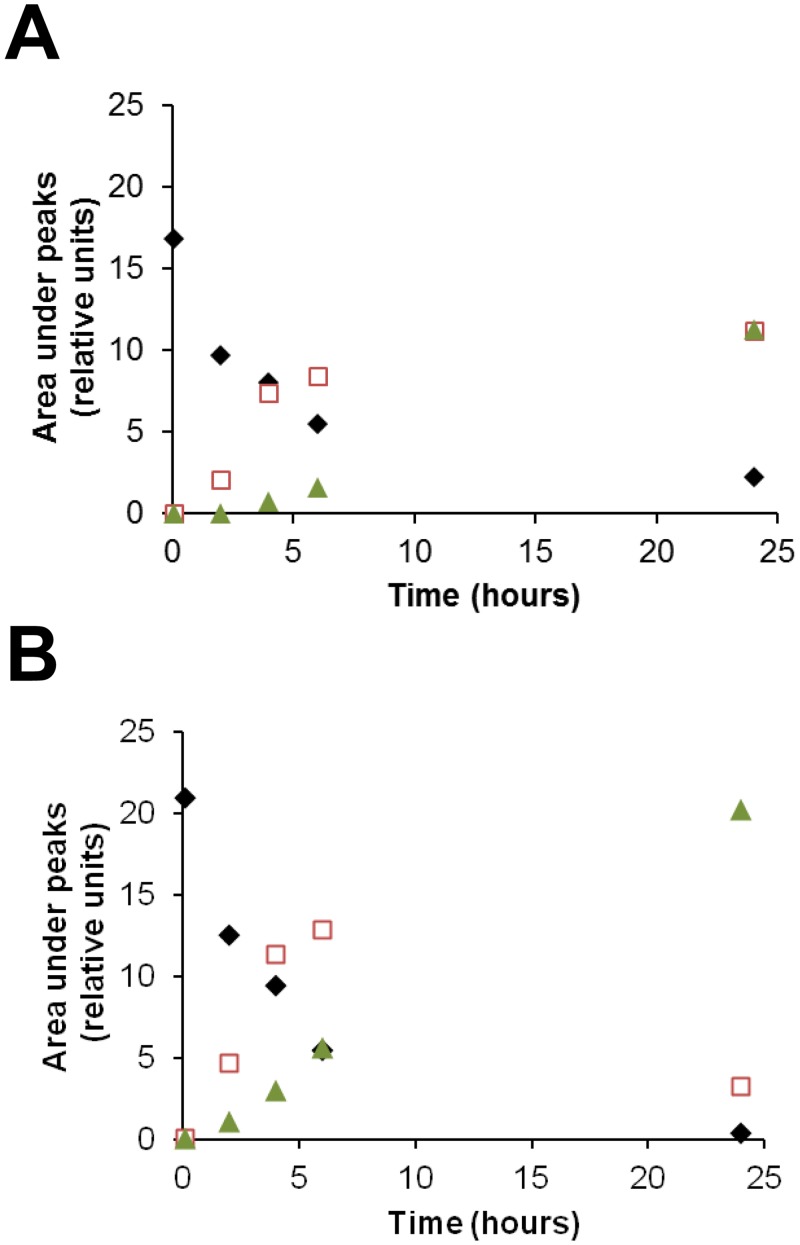
Bisacodyl stability in GSCs’ culture media. Bisacodyl was dissolved in freshly prepared culture medium used for the culture of proliferating cells (A) or in conditioned culture medium of quiescent TG1 GSCs (B) at a final concentration of 10 μM in 1% DMSO. The presence of bisacodyl (diamonds) and its deacetylated derivatives (mono- (squares) and di- (triangles) deacetylated forms), was followed as a function of time. Ordinate represents area under the HPLC elution peaks of the different molecular species present.

**Fig 9:**
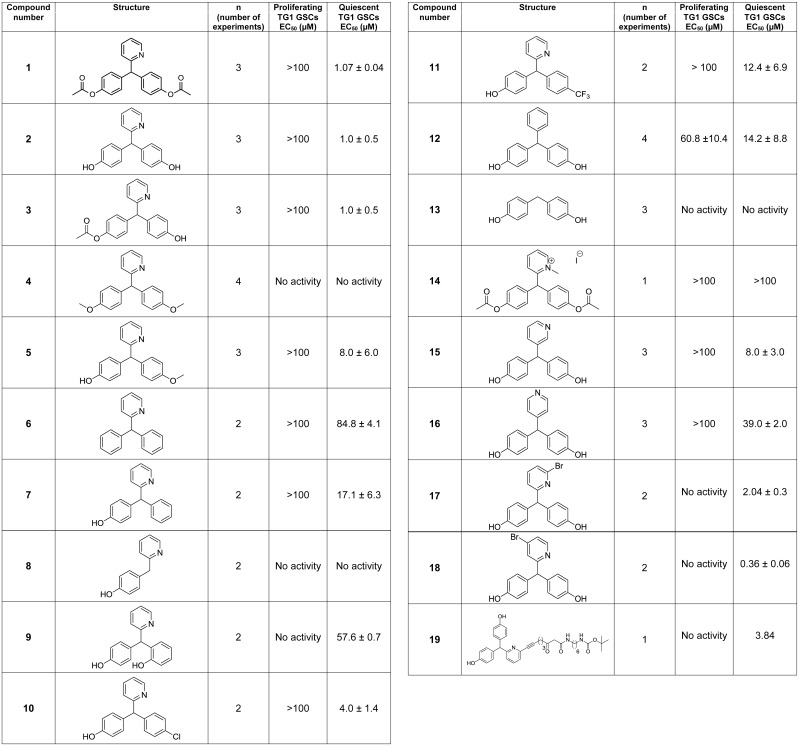
Structure-activity data. Tables representing structure-activity data of bisacodyl (compound **1**) and bisacodyl derivatives (compounds **2**–**19**) on proliferating and quiescent TG1 glioblastoma stem-like cells (GSCs). The ATP-Glo cell survival assay was used to measure compound activity. Compound structures are shown. Compounds were tested in triplicate in each experiment.

Bisacodyl (Fig 6A upper-right panel) is a marketed laxative compound corresponding to a pro-drug. Following oral administration, it is rapidly converted to its active metabolite 4,4'-dihydroxydiphenyl-2-pyridyl-methane (DDPM) also known as BHPM, through hydrolysis of its two acetyl groups [32]. To get deeper insight into the active compound under our experimental conditions, stability of bisacodyl in GSCs' culture medium as well as activity of DDPM on TG1 GSCs were investigated. Stability of bisacodyl in the culture media (freshly prepared medium for proliferating conditions, conditioned medium in contact with quiescent cells for 9 days for quiescent conditions) was followed as a function of time using HPLC as indicated under Materials and Methods. Bisacodyl eluted from the column as a peak at 1.78 min. In proliferating condition medium, this peak disappeared with a half-life of 4 hours whereas a half-life of 2 hours was observed in quiescent cell conditioned medium (Fig 8A and 8B). Concomitantly, in both proliferating condition culture medium and quiescent cell conditioned medium, two other peaks appeared on the chromatogram. One of them, with a 1.56 min retention time, was transient and corresponds to the monoester derivative of bisacodyl (compound **3** in Fig 9). The second, with a 1.35 min retention time, corresponds to DDPM (compound **2** in Fig 9). As shown in Fig 8B, DDPM was the only derivative present in the quiescent TG1 GSCs' conditioned medium after 24h. Evolution of the areas under the three peaks as a function of time (Fig 8A and 8B) is in favor of the hydrolysis of bisacodyl (Fig 6A upper-right panel and compound **1**, Fig 9) in the culture medium and formation of DDPM (compound **2**, Fig 9). As ATP level readouts were performed after a 24h of GSCs' incubation with bisacodyl, one might suggest that the effect observed was due to DDPM. The effect of DDPM (obtained commercially or synthesized in-house) on the relative ATP level of TG1 GSCs was therefore evaluated. Experimental results were totally superimposable to those obtained using bisacodyl (data not shown) with the same specificity towards TG1 GSCs grown under quiescent conditions, suggesting that bisacodyl (Fig 6A upper-right panel and compound **1**, Fig 9) is hydrolyzed to its active DDPM derivative (compound **2**, Fig 9) in the cell culture media of both proliferative and quiescent TG1 GSCs. A similar activity profile was obtained with compound **3** (Fig 9), the monoester derivative of bisacodyl.

### Bisacodyl pharmacophore identification

With the aim at identifying the pharmacophore elements present on DDPM (compound **2**, Fig 9), additional derivatives were synthesized (Fig 9). The importance of the two hydroxyl groups was investigated through synthesis of non-hydrolysable methoxy- derivatives (compounds **4** and **5**, Fig 9). Compound **4** with two methoxyphenyl rings was inactive on both proliferative and quiescent TG1 GSCs, whereas compound **5** with one free phenol group and one methoxyphenyl ring was, similarly to DDPM, active on quiescent TG1 GSCs only, albeit with a 8 fold higher EC_50_. Compound **6** in which the hydroxyl groups of DDPM (compound **2**) were removed can be considered as inactive, similarly to compound **4**. On the other hand, compound **7** where only one of the two hydroxyl groups of DDPM was removed behaved similarly to compound **5** and exhibited selective activity on quiescent TG1 GSCs with an EC_50_ of 17.1 ± 6.3 μM. Solubility measurements indicated that the lack of activity of compound **6** was not due to reduced solubility (data not shown). The activity of the molecule thus appeared to require at least one phenol group in the structure. The second phenyl ring is however important since a total loss in activity was observed for compound **8** where one of the phenol groups was replaced by hydrogen and compound **9** where one of the two hydroxyl groups was moved to the ortho position. Interestingly, the *p*-chloro- and *p*-trifluoromethyl-phenyl monophenols **10** and **11** retained activity (EC_50_ = 4.0 ± 1.4 μM and 12.4 ± 6.9 μM, respectively) with an excellent selectivity versus proliferating TG1 GSCs, suggesting that pharmacomodulation is possible at this position.

In a second step, the importance of the pyridyl ring and the position of the nitrogen atom on the ring were investigated. Phenyl replacement of the pyridyl ring (compound **12**) resulted in an at least 10 fold activity decrease of DDPM (compound **2**) towards quiescent GSCs. Furthermore, the compound presented activity on cells grown under proliferative conditions, although high concentrations were needed for activity. Removing the pyridyl group (compound **13**) or forming a methyl pyridinium (compound **14**) resulted in a total loss of activity towards TG1 GSCs. The importance of the nitrogen atom position on the pyridyl ring was also investigated. Compounds **15** and **16** with nitrogen in positions *meta* and *para*, respectively, kept their total specificity towards quiescent GSCs. However, their efficacy was decreased compared to DDPM (compound **2**), with a ~8—fold increase in EC_50_ for compound **15** and ~40—fold for compound **16**. Those data suggested a role of the pyridyl ring in the activity of DDPM (compound **2**) towards GSCs and its specificity towards cells grown under quiescent conditions. Optimal activity was obtained with the nitrogen atom at the ortho position of the ring.

We further checked the incidence of pyridyl ring substitutions. As shown in Fig 9, mono bromide substituted derivatives appeared promising. Bromide at position 6 of the pyridyl ring (compound **17**) did not alter activity and specificity. Furthermore, compound **18**, with the bromide at position 4, showed increased activity towards quiescent GSCs compared to compound **1**. DDPM (compound **2**), thus appears amenable for further structure activity relationship (SAR) studies aiming at increasing its activity.

The position 6 was also explored for the production of derivatives that could be used for other purposes and namely target identification. Addition of a linker at this position led to compound **19**, which retained activity and selectivity as DDPM (compound **2**). This result opens the possibility to synthesize substituted resins or to develop derivatives for pull down assays. A target identification program based on related compounds is currently under investigation.

In conclusion, our results indicate that bisacodyl (compound **1**, Fig 9) acts through its deacetylated metabolite, DDPM (compound **2**, Fig 9), and the minimal pharmacophoric pattern consists in a p-hydroxyphenyl-aryl-pyridyl-2-methane. Minor modifications lead to changes in potency and/or specificity though pharmacomodulation appears feasible both on the aryl and pyridinyl nuclei, as currently studied. The SAR study is currently on-going and will be disclosed later.

## Discussion

Scientific literature of the past several years points to the presence of quiescent, slow-cycling cancer stem-like cells in a number of tumor types [14]. Namely, a restricted cell population of Ki-67 negative and nestin positive cells was identified in a murine glioblastoma model and stem-like cells with molecular signatures applying to slow-rate dividing cells were evidenced by a single-cell RNA-seq approach [33, 34]. Tumor cell quiescence including the dormant phenotype observed for cancer stem-like cells *in vivo*, represents a major mechanism underlying treatment resistance or tumor recurrence and/or metastasis several years following initial therapeutic intervention [33, 35, 36]. Cellular quiescence is defined as an actively maintained state of proliferation arrest with cells being able to re-enter the cell cycle in response to proliferating stimuli. Quiescent cells are characterized by the lack of expression of proliferation and apoptotic markers, low rate of BrdU incorporation and label retention properties indicating a low turnover [36]. In this study, we have identified culture conditions that induce *in vitro* quiescence of GSCs. Quiescence of these cells was demonstrated by reduction of EdU incorporation, lowering of Ki-67 expression with concomitant absence of significant variations in the expression of apoptosis-related genes. In addition, increased expression of the cell cycle negative regulatory protein p21 was observed in quiescent TG1 GSCs. Interestingly, our data point out the reversibility of the quiescent state following re-exposure to proliferating culture conditions. In addition, we show that quiescent GSCs obtained *in vitro* retain all the characteristics of their proliferating counterparts including cell surface and stem-cell marker expression, clonal and differentiation properties as well as *in vivo* engraftment ability. Quiescent GSCs as well as their proliferating counterparts were subsequently used in a screening assay aiming at identifying compounds with activity on these cells.

Screening the Prestwick Library using the ATP-Glo cell assay, disclosed 20 molecules that decreased cells’ ATP levels in a dose-dependent manner. Changes in ATP levels may reflect alteration in either energy metabolism and/or cell survival. The complete drop in cell ATP level observed in the presence of most of the active molecules we identified, points to a cytotoxic effect.

The Prestwick Library is mainly composed of FDA-approved drugs plus some natural substances. Most hit molecules can thus be clustered according to the ATC (Anatomic, Therapeutic and Clinic) classification (Table 1). Seven out of the twenty hit molecules belong to anatomic group C of molecules acting on the cardiovascular system, although with different therapeutic indications and pharmacological modes of action. Nine more molecules belong to six other anatomical groups and four molecules are natural products. Among those twenty molecules, only tamoxifen and ellipticine are considered as anti-cancer drugs. The Prestwick Library contains 60 other molecules labeled as anticancer drugs. Most of them are antiproliferative agents. However the experimental screening conditions used in the present study, with short time exposure of cells to chemical compounds (24h compared to 48h or 72h classically), did not uncover those anticancer molecules. Tamoxifen, an estrogen receptor (ER) modulator derived from triphenylethylene, is used for ER-positive breast cancer treatment and prevention of breast cancer in high-risk women [37]. At doses in the micromolar range, the compound also exerts ER independent cytotoxic activity on different cell types [38–40]. Cytotoxicity towards human and rat glioma cell lines was reported to occur *via* PKC, PI3K/AKT, JNK and ERK signaling pathways [41, 42]. Tamoxifen has also been evaluated, in association with temozolomide, as a second line therapeutic strategy for the treatment of recurrent glioblastoma [43]. Recently, apoptotic cell death induced by tamoxifen (with EC_50_ values of approximately 30 μM) has been reported in the tumorigenic human neural glial cell line HNGC-2 which forms neurospheres and presents neural stem cell markers [44]. This is in agreement with the cytotoxic effect on human GSCs observed in the present study. However, our data also point to the lack of specificity of the molecule at those doses, as it presents the same efficacy towards non-cancerous cells such as human astrocytes and neural stem cells. In addition, the activity of the molecule does not seem to be related to the proliferative state of the cells, as similar efficacy was observed for proliferative and quiescent GSCs.

The second molecule, ellipticine, is a planar alkaloid isolated from *Apocyanaceae* plants. A methyl-hydroxy derivative (Celiptium) was developed by Sanofi for treating metastatic breast malignancies, but the drug showed numerous adverse effects. Besides its intercalating properties and inhibition of topoisomerase II, ellipticine and derivatives act on a variety of targets [45–49]. Ellipticine presents cytotoxicity towards various cancer cell lines including U-87 MG glioblastoma cells [50–53]. Recently, the molecule has been shown to reduce proliferation and self-renewal ability of aldehyde dehydrogenase 1 class A1-positive breast cancer stem cells [54]. On GSCs treated for 24h, the cytotoxic activity of ellipticine is uncovered at concentrations > 10 μM. A difference in sensitivity between GSCs can be observed as OB1 and TG16 GSCs appear more sensitive to the molecule when grown under proliferative conditions, whereas TG1 cells exhibit the same sensitivity, whether proliferating or quiescent. However, the cytotoxic activity of ellipticine is not specific to cancer cells and the molecule acts, even with higher efficacy, on non-cancer cells (HA cells and f-NSCs in the present study).

Among the hit molecules retained in our screening setting and used in other non-cancer indications, some were already reported to reduce cancer cell viability. Among the molecules in group 1 (Table 1), the vasodilator drug prenylamine has been shown to induce an intracellular calcium increase and cell death in human ovarian tumor cells at a concentration of 100 μM [55]. However, this molecule shows high risks of cardiac arrhythmias and its antiproliferative activities were not further investigated. Fendiline, known as a L-type calcium channel blocker was shown to inhibit, in a calcium independent manner, the survival of human oral cancer cells (OC2) at concentrations between 5–25 μM [56]. In the same concentration range, fendiline inhibits K-Ras signaling in a variety of cancer cells (pancreatic, colon, lung and endometrial) that express oncogenic K-Ras and, as a result, their proliferation [57]. The calcium antagonist vasodilatator perhexiline was also reported to decrease proliferation of the human colon cancer cell line HT29 with an EC_50_ of ca 10 μM [58], and in the micromolar concentration range, to stimulate phagocytosis and induce mTORC1 inhibition in MCF7 cells [59]. These latter two activities were shared, at concentrations > 10μM, with amiodarone, a highly prescribed antiarrhythmic agent which acts on diverse ion channels, namely the (Na^(+)^/Ca^(2+)^ exchanger (NCX), L-type Ca^(2+)^ channels and Na^(+)^ channels. Antiproliferative activities of the molecule on prostate cancer cells through potassium channel inhibition was reported earlier [60].

The serotonin reuptake inhibitor sertraline was shown to induce apoptosis of PC-3 cells [61] and human MG63 osteosarcoma cells [62] at concentrations of ca 30 μM. The drug also exerted a cytotoxic activity on the U-87 human GBM cell line with an EC_50_ of 8 μM and acted in synergy with imatinib to decrease U-87 cell proliferation *via* a drastic decrease in Akt phosphorylation and in an additive manner with temozolomide [63]. An antiproliferative phospho-Akt dependent activity of the drug has also been reported for melanoma cells [64]. Clinical trials using sertraline in combination with standard treatments have been performed for glioblastoma and colon cancer [65, 66]. The molecule also appeared in the list of nine repurposed drugs that could be used as adjuvants in new innovative GBM care [67]. In the present study, we show that sertraline also exhibits cytotoxicity towards GSCs at concentrations of ca 20 μM. However it shows the same potency towards the non-cancerous human astrocytes.

Cancer cell growth inhibitory effects and chemosensitization have also been reported for the anthelmintic drug ivermectin on human ovarian cancer cells [68, 69] and leukemia cells [70]. The molecule acted in the 5–20 μM range through mechanisms such as inhibition of the oncogenic protein kinase PAK1 [68, 69], inhibition of importin/HE4 (human epididymis protein 4) [71] or increase in ROS production [70]. Antiproliferative activities and chemosensitization are also properties of beta escin, a natural mixture of triterpenoid saponins which acts on leukemia cells [72], hepatocellular carcinoma [73, 74], cholangiocarcinoma [75], human lung carcinoma A549 cells [76], human acute leukemia Jurkat T cells [77], human colon carcinoma cell lines [78] and pancreatic cancer cells [79]. Several mechanisms appear to sustain the activity of the molecule, namely apoptosis [75], downregulation of JAK/STAT pathways [74, 76] and inhibition of nuclear kappa B factor [80, 81]. The involvement of the antihypertensor and antianginal felodipine in cancer cell death is not very well documented although one report involved this compound in cholangiocarcinoma [82].

Thus, most molecules of group 1, showed activity on other (non stem-like) cancer cell types (even if information is sometimes limited) at concentrations in the same range as those reported in Table 1 for their cytotoxicity on proliferating or quiescent GSCs. However, at these concentrations, the molecules were also cytotoxic towards non cancer cells and namely astrocytes, HEK 293 cells and neural stem cells.

Concerning molecules of group 2, the histamine H1 receptor antagonist astemizole, currently used as antimalarial and antihistaminic drug, has been proposed as a potential chemotherapeutic agent [83–85]. Its anti-proliferative activity occurs through different mechanisms, namely by targeting proteins involved in cancer progression including human ether a-go-go 1 (hEag1) and Eag-related gene (hErg) potassium channels [83, 85–89] and by modulating autophagy [84]. Terfenadine, a second histamine H1 receptor antagonist has previously been reported for its apoptosis-inducing properties against a variety of cancer cells [90–92], including human melanoma cells [93, 94] and more recently prostate cancer cells [95]. The molecule appears to act through a variety of mechanisms, some implying changes in calcium homeostasis or ROS production. So far, there are no reports of terfenadine effects on human glioblastoma. In the present study we show that terfenadine and astemizole show cytotoxic activity on GSCs with higher efficacy on proliferating cells (EC_50_ of ca 10 μM; comparable to the one measured for other cancer cells). The molecules remain active on quiescent cells, but EC_50_ values are increased by about two fold. As for the other molecules already discussed, there is no specificity for cancer cells compared to non-cancer cells. In addition, their activity on ether a-go-go K^+^ channels (IC_50_ in the nanomolar range) also sustains the cardiotoxic effect of these molecules with QT interval prolongation and torsades de pointe [96–99].

Cytotoxic activity of the sesquiterpene lactone parthenolide has been reported several decades ago [100, 101]. Since, the molecule was shown to act on various cancer cell types including GBM cells [102–104] in which it induced apoptosis *via* an AKT/NF-kB dependent pathway [104]. However, as for other cancer cells, more pathways may be activated [105–107]. EC_50_ values between 13 and 29 μM were determined for different GBM cells (U373, U-87, C6 and U138 MG), similar to those reported here for proliferative GSCs (Table 1). Astrocytes appeared less sensitive to parthenolide with an EC_50_ value of 186 ± 10 μM in the literature [104] and 59 ± 4 μM in our study. Recently, structurally related molecules with activity towards a variety of cancer cells in the nanomolar and low micromolar range were obtained [108]. As far as the neuroleptic zuclopenthixol is concerned, only one publication relates its possible involvement in apoptosis of mouse lymphoma cells. [109]. Zuclopenthixol is the cis-isomer of clopenthixol, a mixed antagonist of dopaminergic G-protein coupled receptors D1 and D2 [110]. A comparative GPCR receptor expression study in TG1, OB1, HA and f-NSC cells performed in our laboratory [31] pointed to DR2 receptor expression in these cells. However, the high concentrations of zuclopenthixol required to induce cytotoxicity do not suggest the involvement of this receptor in GSC cell death induced by this compound.

Molecules in group 3 correspond to compounds with selectivity towards quiescent GSCs. Interestingly, two compounds of this group, ethacrynic acid (EA) and luteolin, are known in the literature as chemoadjuvants or for their anticancer properties towards a variety of cancer cell types. EA, a loop diuretic which inhibits the Na^(+)^-K^(+)^-2Cl^(-)^ kidney symport, presents additional effects and namely inhibitory activity towards glutathione S-transferase [111] which was used to overcome chemotherapeutic drug resistance [112–115], including in GBM [116]. Besides, EA was also shown as an inhibitor of multiple myeloma [117–119], renal cancer, as well as of chronic lymphocytic leukemia cell survival [120] by interfering with the Wnt/beta-catenin signaling pathway [121]. Depending on the cell type, concentrations between ca 40 and 230 μM were required to induce cell death. At those high concentrations, cytotoxicity of the molecule can be observed for TG1 and OB1 GSCs in the proliferative and quiescent state (Table 1). As we did not test our molecules at concentrations above 100 μM, no information can be given for an effect of EA at high concentrations on proliferative TG16. Among the non-cancer cells tested here, f-NSCs appear the most sensitive to EA with an EC_50_ of 21 ± 11 μM. This difference in activity of EA depending on the cell type or physiological state of the cell may be further explored.

Luteolin, the second molecule of group 3 with known potential anticancer activity is a bioflavonoid (3',4',5,7-tetrahydroxy-flavone) found in various plants, including dietary food vegetables. The molecule shows antimicrobial, anti-inflammatory, antioxidant and anticancer activities [122–124]. The efficacy of the molecule relies on a variety of mechanisms as reviewed in [122, 125–127]. More recently, apoptosis and inhibition of the JAK/STAT3 pathway in cholangiocarcinoma [128], upregulation of miR34a in gastric cancer cells [129], apoptosis and upregulation of Fas signaling in Hep-2 cells [130], apoptosis and interference with PI3K/Akt/mTOR signaling in non-small cell lung cancer [131], cell cycle arrest and apoptosis of liver cancer cell lines [132], ROS generation and intracellular copper mobilization in different cancer cell types [133] and inhibition of cyclin G-associated kinase in PC-3 prostate cancer cells [134] were reported for luteolin. Effective concentrations vary, depending on the cell type and the time of exposure, between 8 and 50 μM. Concerning neural cancers, it was shown that under non cytotoxic doses (15–30 μM), luteolin interferes with GBM cell migration [135]. Only one publication deals with luteolin effects on glioma stem-like cells and shows that treatment of U251 glioma stem-like cells with 10 μM luteolin significantly inhibits their sphere forming abilities [136]. In the present study, cytotoxicity of luteolin at concentrations below 100 μM was only observed for GSCs in the quiescent state, pointing to differences in signaling pathways between the two states. The present study also discloses a higher resistance of proliferating GSCs towards luteolin compared to other cells tested in the literature (which, with one exception, were non stem-like cells). Interestingly, under the concentrations tested, luteolin did not show any effect on astrocytes and HEK 293 cells and high concentrations are required to affect f-NSCs. Thus, through its pleotropic targets, luteolin appears as a potential therapeutic agent, but a poor pharmacological tool which does not allow better knowledge of GSCs' pathophysiology.

No cytotoxicity or antiproliferative activity towards cancer cells was attributed so far to the remaining compounds identified in our screening, *i*.*e*. suloctidil and lynestrenol in group 1 and bisacodyl and progesterone in group 3. Suloctidil is a vasodilator and anti-platelet agent that has recently been found in a repurposing screen at 50 μM to have fungilytic activity on Cryptococcus neoformans (a property shared with several other molecules found in the present screen, namely perhexiline, tamoxifen, amiodarone and sertraline [137]. However, as opposed to the other molecules, no antitumor activity has been reported for this compound. Its activity profile on GSCs and non-cancer cell lines is similar to the one observed for perhexiline, amiodarone or sertraline, which were documented as affecting cancer cell survival. Thus, at concentrations in the low micromolar range, suloctidil might be considered as a cytotoxic agent, with no specificity towards cancer cells.

The stimulant laxative bisacodyl appeared in this study as a molecule with highly selective cytotoxicity towards GSCs in their quiescent state and no adverse effect towards non-cancer neural cells. Furthermore, the molecule exerts its unique cytotoxic activity with the highest efficacy (EC_50_ value in the micromolar range) compared to the other molecules tested and this activity was observed for temozolomide-resistant GSCs [23] originating from GBM of three different patients and presenting different molecular signatures (*i*.*e*. p53 mutation is observed in TG16 but not in TG1 or OB1 cells). We showed that in the culture medium, bisacodyl is transformed into DDPM, its known *in vivo* active metabolite. Similar results on cell viability were obtained using either bisacodyl or DDPM. The key structural elements necessary to retain activity and/or specificity have been characterized. They consist in the p-phenolarylpyridinylmethane scaffold with some limited pharmacomodulation freedom on the aryl and pyridine moieties that remain to be further explored. DDPM and its mono bromide substituted derivative corresponding to compound **18** presented in this study remain the most potent and specific compounds identified so far.

The molecular mechanisms underlying the cytotoxic effect of bisacodyl/DDPM on GSCs as well as the direct molecular target(s) of the active metabolite are under investigation. The laxative effect of bisacodyl is thought to occur *via* a mechanism involving ion channel (Na^+^/K^+^) perturbation at the intestinal level. However, few mechanistic studies are available for this old drug marketed in the sixties. The present finding appeals further studies to unveil both the specific activity of bisacodyl/DDPM on quiescent GSCs and some aspects of GSC functioning. Finding a molecule with selective cytotoxic activity on quiescent GSCs is very attractive as cancer stem-like cells are known to be particularly chemo- and radio-resistant. Their ability to enter quiescence *in vivo* reinforces their propensity to escape chemotherapies [14]. To our knowledge, bisacodyl/DDPM is the first small molecule endowed with cytotoxic activity towards slow-growing cancer stem-like cells. None of the chemical screens described in the literature investigated cancer stem-like cells under dormant conditions, whatever the cancer type considered [12, 13, 138, 139]. The present finding opens new avenues for an old molecule with an original mode of action towards cells designated as central culprits in cancer maintenance and relapse after therapy.

## Supporting Information

S1 Fig Expression of surface markers in proliferating and quiescent GSCs. (A-B) Immunohistochemical staining of proliferating and quiescent TG1 and OB1 GSCs with CXCR4 (A) and CD56 (B) antibodies. Nuclei are stained with DAPI. Image magnification: 40x. Scale bars, 20 μm. (C) Expression of the CD133 surface marker was evaluated in proliferating (P) and quiescent (Q) TG1 and OB1 GSCs using flow cytometry (lower panels). Controls using FITC-conjugated antibody isotypes are shown (upper panels). The percentage of CD133 positive cells is indicated.
(TIF)

S2 Fig Evaluation of clonal properties of proliferating and quiescent GSCs. (A) Evaluation of the clonal properties of proliferating (─) and quiescent (▲) TG1 cells. The graph represents the mean number of spheres obtained after 3 weeks in culture as a function of the initial number of cells in a well. (B) Evaluation of the clonal properties of proliferating (─) and quiescent (▲) OB1 cells. Results are represented as in (A). Images of clonal neurospheres obtained from TG1 and OB1 GSCs after 3 weeks are shown as inserts in (A) and (B), respectively.
(TIF)

S3 Fig Differentiation ability of proliferating and quiescent TG1 and OB1 GSCs. (A) Phase contrast images of proliferating (P) and quiescent (Q) GSCs dissociated and incubated in culture medium supplemented with 10% fetal calf serum (FCS) for 0 (d0) to 14 days (d14). Scale bars: 100 μm. (B-E) Histograms representing mRNA expression levels of the sonic hedgehog (SHH), β3-tubulin (TUBB3) and glial fibrillary acid protein (GFAP) genes in proliferating (P) and quiescent (Q) TG1 (B, C) and OB1 (D, E) GSCs cultured in differentiation inducing medium containing 10% FCS for 0 (d0) to 14 days (d14). Results, related to the cycle threshold (Ct) value obtained for each gene, were normalized to the 18S rRNA expression levels in each condition and are shown as fold change (2^∧-ΔΔCt^) observed in proliferating or quiescent TG1 or OB1 cells after 14 days in the presence of serum compared to the expression observed in the same cells at day 0. Data are from two independent experiments.
(TIF)

S4 Fig *In vivo* engraftment properties of proliferating and quiescent GSCs. (A-B) Proliferating (A) or quiescent (B) GSCs were grafted in the left striatum of nude mice. Mice were sacrificed 8 weeks later and brain sections were immunostained with an antibody recognizing specifically human vimentin. Brown vimentin staining thus allows identifying human cells. Scale bar in insert images of the whole brain indicating the region containing human GSCs: 500 μm. Scale bars in enlarged images: 100 μm. (C-D) Immunohistochemical labeling of brain sections obtained as in (A-B) with an anti-Ki-67 antibody.
(TIF)

S1 Methods Expression of stemness, pluripotency and differentiation markers in proliferating and quiescent GSCs. The expression levels (mRNA) of 90 genes enclosing stemness, pluripotency and differentiation markers and 6 endogenous controls (see S2 Table) were studied in both proliferating and quiescent TG1 and OB1 GSCs using the real-time PCR based TaqMan Human Stem Cell Pluripotency Array (Applied Biosystems, Life Technologies). Briefly, total RNA was extracted using TriReagent (Invitrogen). cDNA synthesis was performed with 10 μg of total RNA at 37°C for 2 hours using the High Capacity cDNA Archive kit (Applied Biosystems, Life Technologies). Quantitative PCR was performed using the ABI Prism 7900HT Sequence Detection System (Applied Biosystems). Cycle threshold (Ct) values were measured. Ribosomal RNA18s was used as housekeeping gene. Expression levels of Nanog, GBX2 and IFITM1 were also verified using individual TaqMan gene expression assays from Applied Biosystems, Life Technologies as described in the Materials and Methods section. FACS analysis was used to study Nanog protein expression in proliferating and quiescent TG1 and OB1 GSCs. Neurospheres were dissociated and cells were resuspended in PBS. Fixable viability Dye eFluor 450 labeling (eBioscience) was performed to irreversibly label dead cells. Following washing with PBS, cells were fixed with 2% paraformaldehyde in PBS (10 min at room temperature). Permeabilization (5 min at room temperature) was performed in the presence of 0.1% of saponin and cells were then washed once in HBSS buffer containing saponin (0.1%). Non-specific sites were blocked in PBS solution containing 3% BSA and 2.5% of fetal bovine serum for 30 min at room temperature. Primary antibody labeling (anti-Nanog rabbit polyclonal; Abcam; 1/200) was performed overnight at 4°C. Secondary antibody incubation (1 hour at room temperature) was followed by FACS analysis. Cells labeled with the secondary antibody in the absence of the primary antibody were used as negative controls.
(DOCX)

S2 Methods Expression of surface markers. Detection of CXCR4 and CD56 expression using immunocytochemistry. Cells were mechanically dissociated and centrifuged onto slides with a Cytospin centrifuge. Cellular auto-fluorescence was blocked by incubating cells with 0.06% KMnO4 at room temperature for 10 min. After a blockade with 1% BSA in PBS, cells were incubated with CXCR4 antibodies (Merck Millipore, AB1847, 1:50) or CD56 antibodies (BioLegend, 304601, 1:25) at 4°C overnight. Cells were then washed and incubated with Alexa 488-conjugated secondary antibodies (Jackson Immuno Research, 1:250) for 20 min in the dark at room temperature. DAPI was used for counterstaining before fluorescence microscopy observation. **Evaluation of CD133 expression by flow cytometry**. Following mechanical dissociation and washing in PBS, 1x10^6^ proliferating and quiescent TG1 and OB1 cells were resuspended in 100 μL PBS with 0.5% BSA. Cells were then incubated with 10 μL of FITC-conjugated CD133 antibody (Miltenyi Biotec, 130-105-226) or 10 μL of FITC-conjugated isotype control (Miltenyi Biotec, 130-104-562) for 10 min in the dark in a refrigerator. Cells were washed in 2mL PBS with 0.5% BSA and then analyzed with a FACSCalibur flow cytometer (BD Biosciences). Propidium iodide was used to exclude dead cells.
(DOCX)

S3 Methods Clonality tests. Clonality of proliferating and quiescent TG1 and OB1 GSCs was evaluated in 96-well plates seeded at various cell densities (1, 2, 5, 10, 20 and 50 cells/well) in 0.2 ml of freshly prepared NS34 medium (16 wells/cell density condition). The number of wells containing at least one secondary sphere as well as the number of spheres in each well were evaluated after 3 weeks in culture.
(DOCX)

S4 Methods Evaluation of *in vitro* differentiation properties of GSCs. Proliferating and quiescent (9 days without medium renewal) TG1 and OB1 GSCs were dissociated and seeded in freshly prepared NS34 medium supplemented with 10% FBS (Day 0). Cells were then maintained in this differentiation-inducing culture medium for 14 days. At day 0 and day 14, 2.5–5 x 10^6^ cells in each condition were collected and total RNA was extracted, purified, characterized and reverse transcribed as described in the Materials and Methods section. Expression at the mRNA level of stem cell and differentiation markers (Sonic Hedgehog (SHH): Hs 00179843-m1, β3 tubulin (TUBB3): Hs 00801390-S1 and glial fibrillary acid protein (GFAP): Hs00909233-m1) was determined using individual TaqMan gene expression assays (Applied Biosystems, Life Technologies) and quantitative PCR analysis as described in previous sections.
(DOCX)

S5 Methods Evaluation of *in vivo* engraftment properties of GSCs. Mice (NMRI Nude, Janvier) were anesthetized by inhalation of isoflurane. 10^5^ glioblastoma OB1 cells (proliferating and quiescent) were injected into the left striatum of mice (4 mice per experimental setting), using a stereotaxic set-up with a 10 μL Hamilton syringe. The needle was kept in place for 5 min after injection and then slowly removed. 8 weeks after injection, mice were anesthetized with xylazine, then perfused with 4% formaldehyde. Mouse brains were fixed in 4% formaldehyde, embedded in paraffin and cut into serial 10 μm-thick sections. Human cells were identified with immunohistochemical staining with an antibody against vimentin (clone V9, Dako). Ki-67 positive cells were identified with immunohistochemical staining with corresponding antibodies from Dako (clone MIB-1).
(DOCX)

S6 Methods Experimental section for the synthesis of the chemical compounds. (DOCX)

S7 Methods LC/MS analytical data. (DOCX)

S1 Table Cell source, cell handling and resource sharing information. P: proliferating; Q: quiescent.
(DOCX)

S2 Table Assay ID numbers, IDs and names of genes included in the TaqMan Human Stem Cell Pluripotency Arrays from Applied Biosystems, Life Technologies. List of the 90 stem cell or differentiation associated genes and 6 housekeeping genes (ACTB, RAF1, CTNNB1, GAPDH, EEF1A1, 18S) included in the Human Stem Cell Pluripotency Array from Life Technologies.
(DOCX)

S3 Table Primary and secondary screen hit compounds. GSCs: glioblastoma stem-like cells. Hit compounds selected both in the primary and secondary screens for their activity in at least one of the conditions tested are highlighted in yellow.
(DOCX)

S4 Table Hit compound structures. Molecules were disclosed from the Prestwick Library for their activity on GSCs.
(XLSX)
