# Supplementary figures and images for: Chemical Library Screening and Structure-Function Relationship Studies Identify Bisacodyl as a Potent and Selective Cytotoxic Agent Towards Quiescent Human Glioblastoma Tumor Stem-Like Cells
Source: PLoS One. 2015 Aug 13;10(8):e0134793. doi: 10.1371/journal.pone.0134793 (PMC4536076; doi:10.1371/journal.pone.0134793)

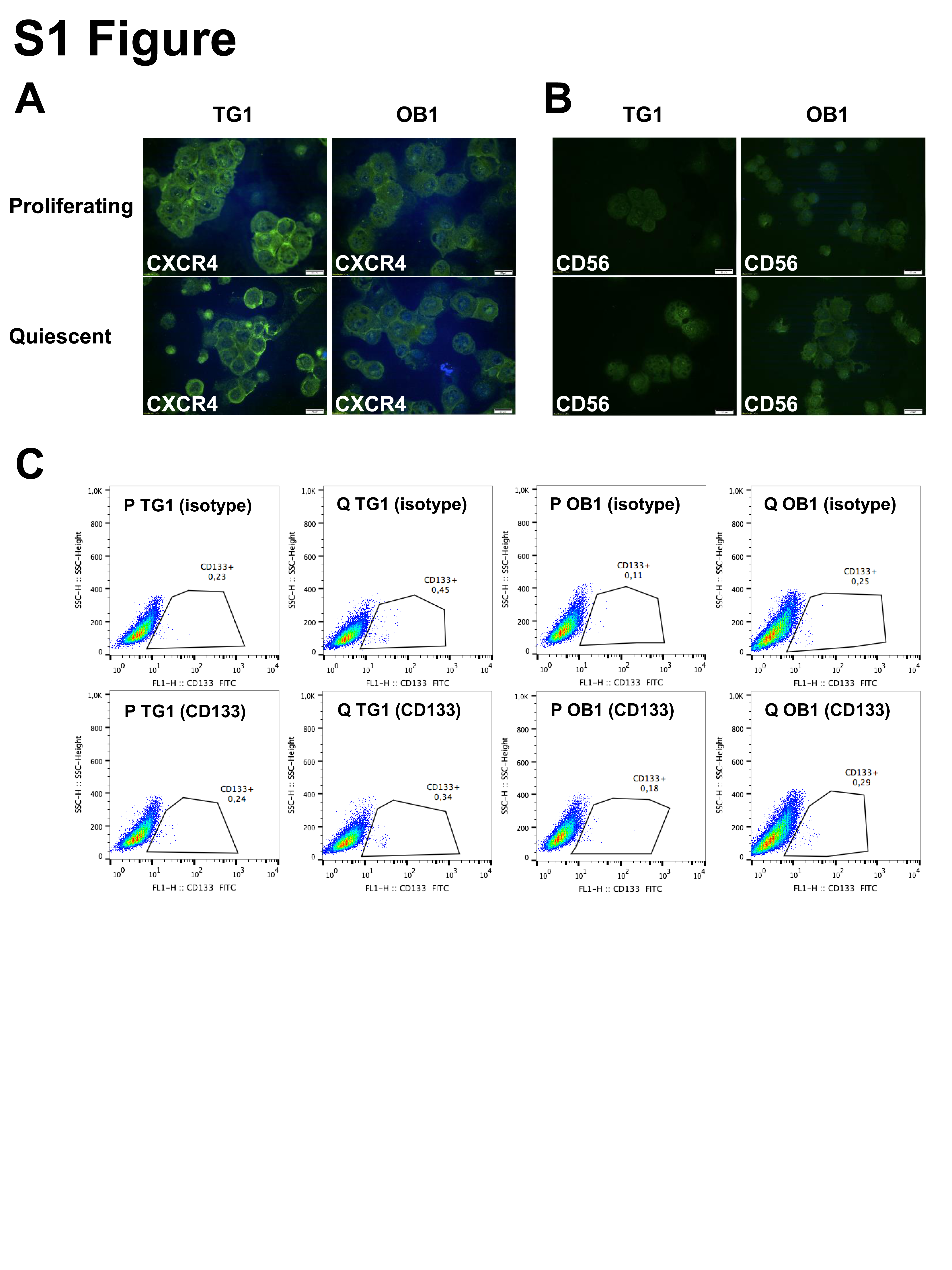

Supplement: S1 Fig — (A-B) Immunohistochemical staining of proliferating and quiescent TG1 and OB1 GSCs with CXCR4 (A) and CD56 (B) antibodies. Nuclei are stained with DAPI. Image magnification: 40x. Scale bars, 20 μm. (C) Expression of the CD133 surface marker was evaluated in proliferating (P) and quiescent (Q) TG1 and OB1 GSCs using flow cytometry (lower panels). Controls using FITC-conjugated antibody isotypes are shown (upper panels). The percentage of CD133 positive cells is indicated. (TIF) [file pone.0134793.s001.tif]

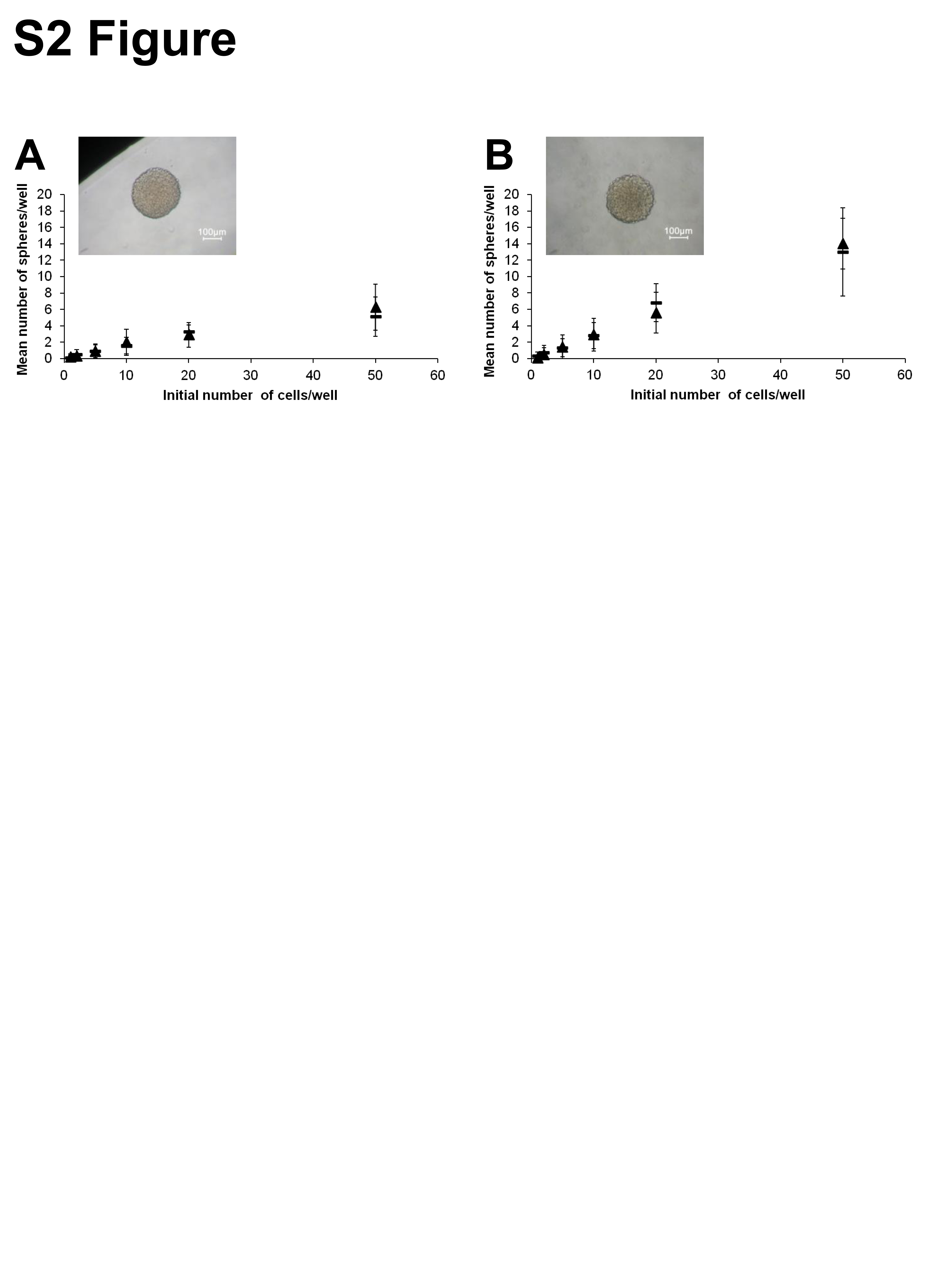

Supplement: S2 Fig — (A) Evaluation of the clonal properties of proliferating (─) and quiescent (▲) TG1 cells. The graph represents the mean number of spheres obtained after 3 weeks in culture as a function of the initial number of cells in a well. (B) Evaluation of the clonal properties of proliferating (─) and quiescent (▲) OB1 cells. Results are represented as in (A). Images of clonal neurospheres obtained from TG1 and OB1 GSCs after 3 weeks are shown as inserts in (A) and (B), respectively. (TIF) [file pone.0134793.s002.tif]

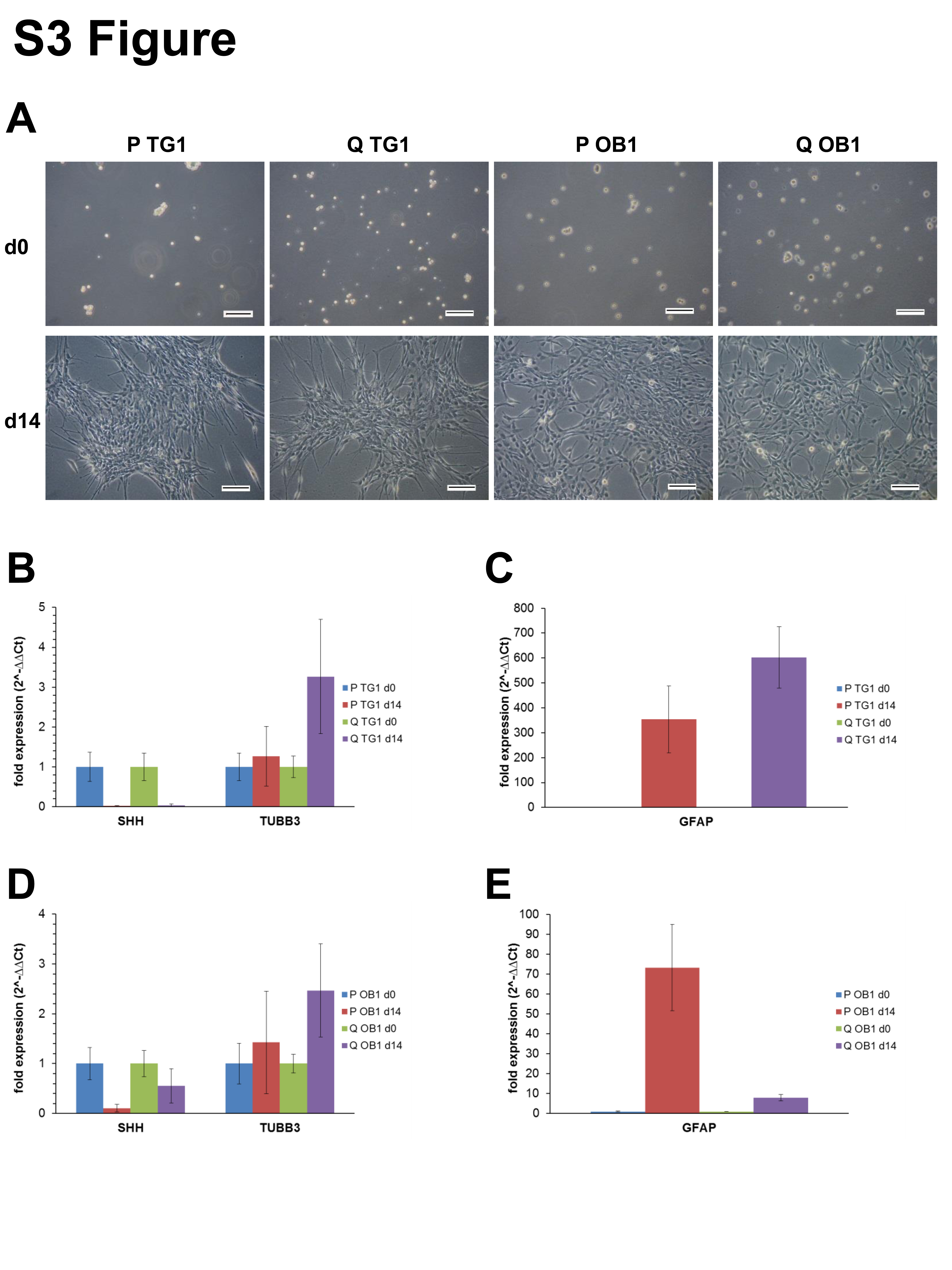

Supplement: S3 Fig — (A) Phase contrast images of proliferating (P) and quiescent (Q) GSCs dissociated and incubated in culture medium supplemented with 10% fetal calf serum (FCS) for 0 (d0) to 14 days (d14). Scale bars: 100 μm. (B-E) Histograms representing mRNA expression levels of the sonic hedgehog (SHH), β3-tubulin (TUBB3) and glial fibrillary acid protein (GFAP) genes in proliferating (P) and quiescent (Q) TG1 (B, C) and OB1 (D, E) GSCs cultured in differentiation inducing medium containing 10% FCS for 0 (d0) to 14 days (d14). Results, related to the cycle threshold (Ct) value obtained for each gene, were normalized to the 18S rRNA expression levels in each condition and are shown as fold change (2∧-ΔΔCt) observed in proliferating or quiescent TG1 or OB1 cells after 14 days in the presence of serum compared to the expression observed in the same cells at day 0. Data are from two independent experiments. (TIF) [file pone.0134793.s003.tif]

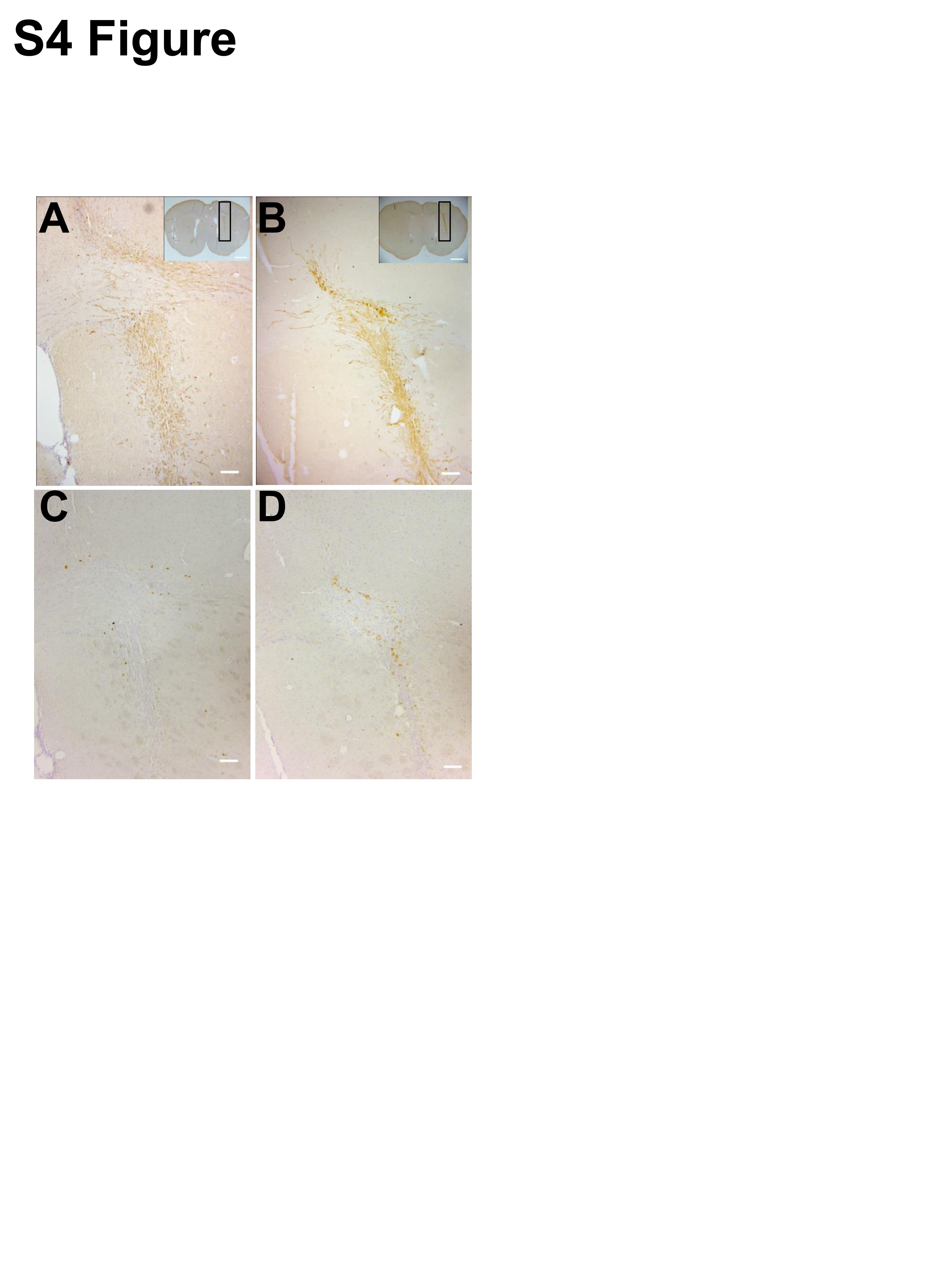

Supplement: S4 Fig — (A-B) Proliferating (A) or quiescent (B) GSCs were grafted in the left striatum of nude mice. Mice were sacrificed 8 weeks later and brain sections were immunostained with an antibody recognizing specifically human vimentin. Brown vimentin staining thus allows identifying human cells. Scale bar in insert images of the whole brain indicating the region containing human GSCs: 500 μm. Scale bars in enlarged images: 100 μm. (C-D) Immunohistochemical labeling of brain sections obtained as in (A-B) with an anti-Ki-67 antibody. (TIF) [file pone.0134793.s004.tif]
