# Supplementary material for: Chemical Library Screening and Structure-Function Relationship Studies Identify Bisacodyl as a Potent and Selective Cytotoxic Agent Towards Quiescent Human Glioblastoma Tumor Stem-Like Cells
Source: PLoS One. 2015 Aug 13;10(8):e0134793. doi: 10.1371/journal.pone.0134793 (PMC4536076; doi:10.1371/journal.pone.0134793)
Supplement: S1 Methods — The expression levels (mRNA) of 90 genes enclosing stemness, pluripotency and differentiation markers and 6 endogenous controls (see S2 Table) were studied in both proliferating and quiescent TG1 and OB1 GSCs using the real-time PCR based TaqMan Human Stem Cell Pluripotency Array (Applied Biosystems, Life Technologies). Briefly, total RNA was extracted using TriReagent (Invitrogen). cDNA synthesis was performed with 10 μg of total RNA at 37°C for 2 hours using the High Capacity cDNA Archive kit (Applied Biosystems, Life Technologies). Quantitative PCR was performed using the ABI Prism 7900HT Sequence Detection System (Applied Biosystems). Cycle threshold (Ct) values were measured. Ribosomal RNA18s was used as housekeeping gene. Expression levels of Nanog, GBX2 and IFITM1 were also verified using individual TaqMan gene expression assays from Applied Biosystems, Life Technologies as described in the Materials and Methods section. FACS analysis was used to study Nanog protein expression in proliferating and quiescent TG1 and OB1 GSCs. Neurospheres were dissociated and cells were resuspended in PBS. Fixable viability Dye eFluor 450 labeling (eBioscience) was performed to irreversibly label dead cells. Following washing with PBS, cells were fixed with 2% paraformaldehyde in PBS (10 min at room temperature). Permeabilization (5 min at room temperature) was performed in the presence of 0.1% of saponin and cells were then washed once in HBSS buffer containing saponin (0.1%). Non-specific sites were blocked in PBS solution containing 3% BSA and 2.5% of fetal bovine serum for 30 min at room temperature. Primary antibody labeling (anti-Nanog rabbit polyclonal; Abcam; 1/200) was performed overnight at 4°C. Secondary antibody incubation (1 hour at room temperature) was followed by FACS analysis. Cells labeled with the secondary antibody in the absence of the primary antibody were used as negative controls. (DOCX) [file pone.0134793.s005.docx]

**S1 Methods. Expression of stemness, pluripotency and differentiation markers in proliferating and quiescent GSCs.** The expression levels (mRNA) of 90 genes enclosing stemness, pluripotency and differentiation markers and 6 endogenous controls (see S2 Table) were studied in both proliferating and quiescent TG1 and OB1 GSCs using the real-time PCR based TaqMan Human Stem Cell Pluripotency Array (Applied Biosystems, Life Technologies). Briefly, total RNA was extracted using TriReagent (Invitrogen). cDNA synthesis was performed with 10 µg of total RNA at 37°C for 2 hours using the High Capacity cDNA Archive kit (Applied Biosystems, Life Technologies). Quantitative PCR was performed using the ABI Prism 7900HT Sequence Detection System (Applied Biosystems). Cycle threshold (Ct) values were measured. Ribosomal RNA18s was used as housekeeping gene. Expression levels of Nanog, GBX2 and IFITM1 were also verified using individual TaqMan gene expression assays from Applied Biosystems, Life Technologies as described in the Materials and Methods section. FACS analysis was used to study Nanog protein expression in proliferating and quiescent TG1 and OB1 GSCs. Neurospheres were dissociated and cells were resuspended in PBS. Fixable viability Dye eFluor® 450 labeling (eBioscience) was performed to irreversibly label dead cells. Following washing with PBS, cells were fixed with 2 % paraformaldehyde in PBS (10 min at room temperature). Permeabilization (5 min at room temperature) was performed in the presence of 0.1 % of saponin and cells were then washed once in HBSS buffer containing saponin (0.1 %). Non-specific sites were blocked in PBS solution containing 3 % BSA and 2.5 % of fetal bovine serum for 30 min at room temperature. Primary antibody labeling (anti-Nanog rabbit polyclonal; Abcam; 1/200) was performed overnight at 4°C. Secondary antibody incubation (1 hour at room temperature) was followed by FACS analysis. Cells labeled with the secondary antibody in the absence of the primary antibody were used as negative controls.
