# Supplementary material for: Chemical Library Screening and Structure-Function Relationship Studies Identify Bisacodyl as a Potent and Selective Cytotoxic Agent Towards Quiescent Human Glioblastoma Tumor Stem-Like Cells
Source: PLoS One. 2015 Aug 13;10(8):e0134793. doi: 10.1371/journal.pone.0134793 (PMC4536076; doi:10.1371/journal.pone.0134793)
Supplement: S2 Methods — Cells were mechanically dissociated and centrifuged onto slides with a Cytospin centrifuge. Cellular auto-fluorescence was blocked by incubating cells with 0.06% KMnO4 at room temperature for 10 min. After a blockade with 1% BSA in PBS, cells were incubated with CXCR4 antibodies (Merck Millipore, AB1847, 1:50) or CD56 antibodies (BioLegend, 304601, 1:25) at 4°C overnight. Cells were then washed and incubated with Alexa 488-conjugated secondary antibodies (Jackson Immuno Research, 1:250) for 20 min in the dark at room temperature. DAPI was used for counterstaining before fluorescence microscopy observation. Evaluation of CD133 expression by flow cytometry. Following mechanical dissociation and washing in PBS, 1x10^6^ proliferating and quiescent TG1 and OB1 cells were resuspended in 100 μL PBS with 0.5% BSA. Cells were then incubated with 10 μL of FITC-conjugated CD133 antibody (Miltenyi Biotec, 130-105-226) or 10 μL of FITC-conjugated isotype control (Miltenyi Biotec, 130-104-562) for 10 min in the dark in a refrigerator. Cells were washed in 2mL PBS with 0.5% BSA and then analyzed with a FACSCalibur flow cytometer (BD Biosciences). Propidium iodide was used to exclude dead cells. (DOCX) [file pone.0134793.s006.docx]

**S2 Methods. Expression of surface markers.**

**Detection of CXCR4 and CD56 expression using immunocytochemistry.** Cells were mechanically dissociated and centrifuged onto slides with a Cytospin centrifuge. Cellular auto-fluorescence was blocked by incubating cells with 0.06% KMnO4 at room temperature for 10 min. After a blockade with 1% BSA in PBS, cells were incubated with CXCR4 antibodies (Merck Millipore, AB1847, 1:50) or CD56 antibodies (BioLegend, 304601, 1:25) at 4°C overnight. Cells were then washed and incubated with Alexa 488-conjugated secondary antibodies (Jackson Immuno Research, 1:250) for 20 min in the dark at room temperature. DAPI was used for counterstaining before fluorescence microscopy observation.

**Evaluation of CD133 expression by flow cytometry**. Following mechanical dissociation and washing in PBS, 1x10^6^ proliferating and quiescent TG1 and OB1 cells were resuspended in 100 µL PBS with 0.5 % BSA. Cells were then incubated with 10 µL of FITC-conjugated CD133 antibody (Miltenyi Biotec, 130-105-226) or 10 µL of FITC-conjugated isotype control (Miltenyi Biotec, 130-104-562) for 10 min in the dark in a refrigerator. Cells were washed in 2mL PBS with 0.5 % BSA and then analyzed with a FACSCalibur flow cytometer (BD Biosciences). Propidium iodide was used to exclude dead cells.
