# Supplementary material for: Chemical Library Screening and Structure-Function Relationship Studies Identify Bisacodyl as a Potent and Selective Cytotoxic Agent Towards Quiescent Human Glioblastoma Tumor Stem-Like Cells
Source: PLoS One. 2015 Aug 13;10(8):e0134793. doi: 10.1371/journal.pone.0134793 (PMC4536076; doi:10.1371/journal.pone.0134793)
Supplement: S3 Methods — Clonality of proliferating and quiescent TG1 and OB1 GSCs was evaluated in 96-well plates seeded at various cell densities (1, 2, 5, 10, 20 and 50 cells/well) in 0.2 ml of freshly prepared NS34 medium (16 wells/cell density condition). The number of wells containing at least one secondary sphere as well as the number of spheres in each well were evaluated after 3 weeks in culture. (DOCX) [file pone.0134793.s007.docx]

**S3 Methods. Clonality tests.** Clonality of proliferating and quiescent TG1 and OB1 GSCs was evaluated in 96-well plates seeded at various cell densities (1, 2, 5, 10, 20 and 50 cells/well/) in 0.2 ml of freshly prepared NS34 medium (16 wells/cell density condition). The number of wells containing at least one secondary sphere as well as the number of spheres in each well were evaluated after 3 weeks in culture.
