# Supplementary material for: Chemical Library Screening and Structure-Function Relationship Studies Identify Bisacodyl as a Potent and Selective Cytotoxic Agent Towards Quiescent Human Glioblastoma Tumor Stem-Like Cells
Source: PLoS One. 2015 Aug 13;10(8):e0134793. doi: 10.1371/journal.pone.0134793 (PMC4536076; doi:10.1371/journal.pone.0134793)
Supplement: S4 Methods — Proliferating and quiescent (9 days without medium renewal) TG1 and OB1 GSCs were dissociated and seeded in freshly prepared NS34 medium supplemented with 10% FBS (Day 0). Cells were then maintained in this differentiation-inducing culture medium for 14 days. At day 0 and day 14, 2.5–5 x 10^6^ cells in each condition were collected and total RNA was extracted, purified, characterized and reverse transcribed as described in the Materials and Methods section. Expression at the mRNA level of stem cell and differentiation markers (Sonic Hedgehog (SHH): Hs 00179843-m1, β3 tubulin (TUBB3): Hs 00801390-S1 and glial fibrillary acid protein (GFAP): Hs00909233-m1) was determined using individual TaqMan gene expression assays (Applied Biosystems, Life Technologies) and quantitative PCR analysis as described in previous sections. (DOCX) [file pone.0134793.s008.docx]

**S4 Methods. Evaluation of *in vitro* differentiation properties of GSCs.** Proliferating and quiescent (9 days without medium renewal) TG1 and OB1 GSCs were dissociated and seeded in freshly prepared NS34 medium supplemented with 10% FBS (Day 0). Cells were then maintained in this differentiation-inducing culture medium for 14 days. At day 0 and day 14, 2.5-5 x 10^6^ cells in each condition were collected and total RNA was extracted, purified, characterized and reverse transcribed as described in the Materials and Methods section. Expression at the mRNA level of stem cell and differentiation markers (Sonic Hedgehog (SHH): Hs 00179843-m1, β3 tubulin (TUBB3): Hs 00801390-S1 and glial fibrillary acid protein (GFAP): Hs00909233-m1) was determined using individual TaqMan gene expression assays (Applied Biosystems, Life Technologies) and quantitative PCR analysis as described in previous sections.
