# Supplementary material for: Chemical Library Screening and Structure-Function Relationship Studies Identify Bisacodyl as a Potent and Selective Cytotoxic Agent Towards Quiescent Human Glioblastoma Tumor Stem-Like Cells
Source: PLoS One. 2015 Aug 13;10(8):e0134793. doi: 10.1371/journal.pone.0134793 (PMC4536076; doi:10.1371/journal.pone.0134793)
Supplement: S5 Methods — Mice (NMRI Nude, Janvier) were anesthetized by inhalation of isoflurane. 105 glioblastoma OB1 cells (proliferating and quiescent) were injected into the left striatum of mice (4 mice per experimental setting), using a stereotaxic set-up with a 10 μL Hamilton syringe. The needle was kept in place for 5 min after injection and then slowly removed. 8 weeks after injection, mice were anesthetized with xylazine, then perfused with 4% formaldehyde. Mouse brains were fixed in 4% formaldehyde, embedded in paraffin and cut into serial 10 μm-thick sections. Human cells were identified with immunohistochemical staining with an antibody against vimentin (clone V9, Dako). Ki-67 positive cells were identified with immunohistochemical staining with corresponding antibodies from Dako (clone MIB-1). (DOCX) [file pone.0134793.s009.docx]

**S5 Methods. Evaluation of *in vivo* engraftment properties of GSCs.** Mice (NMRI Nude, Janvier) were anesthetized by inhalation of isoflurane. 10^5^ glioblastoma OB1 cells (proliferating and quiescent) were injected into the left striatum of mice (4 mice per experimental setting), using a stereotaxic set-up with a 10 µL Hamilton syringe. The needle was kept in place for 5 min after injection and then slowly removed. 8 weeks after injection, mice were anesthetized with xylazine, then perfused with 4% formaldehyde. Mouse brains were fixed in 4% formaldehyde, embedded in paraffin and cut into serial 10 µm-thick sections. Human cells were identified with immunohistochemical staining with an antibody against vimentin (clone V9, Dako). Ki-67 positive cells were identified with immunohistochemical staining with corresponding antibodies from Dako (clone MIB-1).
