# Supplementary material for: Chemical Library Screening and Structure-Function Relationship Studies Identify Bisacodyl as a Potent and Selective Cytotoxic Agent Towards Quiescent Human Glioblastoma Tumor Stem-Like Cells
Source: PLoS One. 2015 Aug 13;10(8):e0134793. doi: 10.1371/journal.pone.0134793 (PMC4536076; doi:10.1371/journal.pone.0134793)
Supplement: S6 Methods — (DOCX) [file pone.0134793.s010.docx]

**S6 Methods. Experimental section for the synthesis of the chemical compounds**

Access to compounds **1-19** is represented in Schemes **1-6** given below:

**Scheme 1.** **Preparation of de-acylated and methoxylated analogs of bisacodyl (compound 1).*^a^***

***^a^*** Reagents and conditions: (i) LiOH, H_2_O/1,2-dimethoxyethane, room temperature (rt); (ii) KOH in EtOH (10% w/w), rt, (iii) NaH, MeI, DMF, 0°C, (iv) NaH, MeI, DMF, 0°C.

**Scheme 2.** **Preparation of symmetrical bisacodyl derivatives.*^b^***

***^b^***Reagents and conditions: (i) triflic acid (ii) NaHCO_3_

**Scheme 3.** **Preparation of compound 7**.***^c^***

***^c^*** Reagents and conditions: (i) (CF_3_SO_2_)_2_O, Et_3_N, CH_2_Cl_2_, rt; (ii) HCOOH, Et_3_N, PPh_3_, Pd(OAc)_2_, DMF, rt.

**Scheme 4.** **Preparation of bis-aryls derivatives of bisacodyl.*^d^***

***^d^*** Reagents and conditions: (i) 2-bromopyridine, *n*BuLi, Et_2_O, -78 ^o^C to rt; (ii) HI_aq._ (58% w/w), AcOH, Δ; (iii) BBr_3_, CH_2_Cl_2_, -78 ^o^C.

**Scheme 5.** **Preparation of the dissymmetrical chloro and trifluoromethyl derivatives 10 and 11**.^e^

^e^ Reagents and conditions: (i) (CF_3_CO)_2_O, BF_3_•Et_2_O, toluene; (ii) 2-bromopyridine, n-BuLi, Et_2_O, -78^o^C to rt; (iii) HI_aq._ (58% w/w), AcOH, Δ; (iv) BBr_3_, CH_2_Cl_2_, -78^o^C.

**Scheme 6.** **Preparation of 2-(bis(4-acetoxyphenyl)methyl)-1-methylpyridinium iodide**.^f^

^f^ Reagents and conditions: (i) acetone, MeI, room temperature, 48 h.

**General Methods**

Reactions were performed under an argon atmosphere unless specified otherwise. Chemicals and solvents were purchased from Sigma-Aldrich and were used without further purification. Analytical thin layer chromatography (TLC) was performed using silica gel plates Merck 60F254 and plates were visualized by exposure to ultraviolet light. Compounds were purified using Armen spot flash chromatography on silica gel Merck 60 (particle size 0.040-0.063mm). Yields refer to isolated compounds, estimated to be >97% pure as determined by ^1^H NMR or HPLC. ^1^H and ^13^C NMR spectra were recorded at ambient temperature at 400 MHz with tetramethylsilane (TMS) as an internal standard (Bruker UltraShield Plus). Chemical shifts (*δ*) of samples in CDCl_3_, CH_3_OD, [D_6_]acetone or [D_6_]DMSO are in parts per million (ppm). The splitting patterns are designated as: s singlet, br s broad singlet, d doublet, dd doublet of doublets, ddd doublet of doublet of doublets, t triplet, td triplet of doublets, m multiplet, and br m broad multiplet. Coupling constants (*J*) between two nuclei separated by *n* chemical bonds are denoted in Hertz (Hz). FTIR spectra were recorded with a Nicolet 380 apparatus (Thermo). HRMS spectra were recorded with an Agilent Technologies 6520 Q-TOF apparatus. Melting points were recorded with a Büchi B-540 melting point apparatus. Analytical RP-HPLC-MS was performed using a LC-MSD 1200SL Agilent with a Thermo Hypersilgold® column (C18, 30 mm × 1 mm; 1.9 μm) using the following parameters: 1) The solvent system: A (acetonitrile) and B (0.05% TFA in H_2_O); 2) A linear gradient: t = 0 min, 98% B; t = 5 min, 5% B; t = 6 min, 5% B; t = 7 min, 98% B; t = 9 min, 98% B; 3) Flow rate of 0.3 mL/min; 4) Column temperature: 50°C; 5) The ratio of products was determined by integration of spectra recorded at 210 nm or 254 nm; 6) Ionization mode: MM-ES+APCI.

**General method A for the preparation of condensation products 2, 6, 9, 12, 15-18 according to Scheme 1.**^[[1]](#footnote-1)^

The aldehyde (1 mmol) and the aryl derivative (11 mmol, 11 eq) were dissolved at 0 °C in CF_3_SO_3_H (3 mL, 34 mmol, 34 eq). After 3-24 h stirring at room temperature, the mixture was poured onto ice and neutralized with aqueous NaOH (15% w/w). The solution was extracted with EtOAc or CH_2_Cl_2_ (3 x 200 mL) and the organic layers were washed with H_2_O (3 x 150 mL), brine (50 mL), H_2_O (50 mL) and further dried over cotton. The target compound was separated from the crude product using column chromatography. *Note: the para/ortho regioisomer was in most cases isolated as the minor product.*

**General procedures for the preparation of dissymmetrical compounds according to Scheme 5.**

**General method B: access to benzophenone derivatives.**^[[2]](#footnote-2)^

TFAA (1.5 eq) was added to a slurry of benzoic acid derivative (1 eq) and phenyl derivative (46 eq). The resulting mixture was heated for 1 hour at 80°C before BF_3_^.^Et_2_O (2 %mol) was added, and further stirred at this temperature until completion of the reaction (followed by HPLC analysis). EtOH was added and the mixture was first held at reflux for 1 h, then cooled to ambient temperature over 60 min and finally stirred at 0-5°C (NaCl bath) before the solid being filtered off and further washed with EtOH (30 mL) and dried i*n vacuo*.

**General method C: benzophenone alkylation.**^[[3]](#footnote-3)^

nBuLi (1.05 eq; 1.6 M in hexane) was added dropwise at -78 °C to a solution of 2-bromopyridine (1 eq,) 1M in Et_2_O ). The resulting solution was then stirred for 2h below -40 °C before the benzophenone derivative (1 eq) solution in Et_2_O (0.75 M) was added dropwise. The resulting slurry was further stirred overnight at room temperature and quenched with saturated aqueous NH_4_Cl. The volatiles were evaporated in vacuo and the residue was recrystallized from MeOH or purified using column chromatography.

**General method D: dehydroxylation of bis-aryl(pyridin-2-yl)methanol.^[[4]](#footnote-4)^**

The methanol derivative (0.3 mmol) was added to a mixture of concentrated aqueous HI (300 µL; 50% w/w) and acetic acid (1.5 mL). Then the solution was stirred at 100 °C for 4 h, before being cooled to 0 °C, basified to pH 9 with aqueous NaOH (15% w/w) and diluted with CH_2_Cl_2_ (50 mL). The organic layer was washed with saturated aqueous Na_2_S_2_O_3_ and dried over anhydrous MgSO_4_. Final purification was achieved using column chromatography.

**General method E: demethylation.**

BBr_3_ (2 eq; 1 M in CH_2_Cl_2_) was added dropwise at -78 °C to a solution of methoxyphenyl derivative (1 eq, 1M in CH_2_Cl_2_). Then, the solution was stirred at room temperature for 4 h. Saturated aqueous NaHCO_3_ was added (60 mL) and the organic layer separated, dried over cotton and concentrated under vacuum to yield a residue of good purity to be used as such.

**Compounds**

**4,4'-dihydroxydiphenyl(pyridine-2-yl)methane** **(2)**

**Route 1 (Scheme 1)**: 4,4'-(2-pyridinylmethylene)bis-1,1'-diacetate **1** (bisacodyl; 12.7 g, 35.14 mmol, 1 eq) was dissolved in a solution of KOH (10.92 g, 0.195 mol, 5.5 eq) in EtOH (40% w/w) and stirred for 48 h at room temperature. The resulting solution was evaporated to dryness and aq. HCl (10% w/w) was first added followed by saturated aq. NaHCO_3_. The solution was extracted with EtOAc and the organic layers were combined, dried over cotton and evaporated to dryness to give **2** as a white solid (9.22 g, 95 %). R_f_ = 0.64 (CH_2_Cl_2_/EtOAc: 50/50). ^1^H NMR (CD_3_OD) *δ* = 5.5 (s, 1H), 6.71 (d, *J =* 8.5 Hz, 4H), 6.9 (d, *J =* 8.4 Hz, 4H), 7.13 (d, *J =* 7.9 Hz, 1H), 7.24 (dd, *J =* 7.5 Hz, J = 5 Hz, 1H), 7.7 (td, *J =* 7.7 Hz, 1.9 Hz, 1H), 8.44 (d, *J =* 5 Hz, 1H). ^13^C NMR (CD_3_OD) *δ* = 58.7, 116.2, 122.9, 125.4, 131.3, 135.2, 138.6, 149.6, 158.2, 165.5.

**Route 2 (Scheme 2)**: 2-pyridinecarboxaldehyde (95 µL, 1 mmol, 1eq) in phenol (1.035 g, 11 mmol, 11 eq) were dissolved in CF_3_SO_3_H (3 mL, 34 mmol, 34 eq). After 3 h stirring at rt, the resulting orange mixture was poured on ice and neutralized with aq. NaOH (15 % w/w). The solution was extracted with EtOAc (600 mL) and the organic layer further washed with H_2_O (3 x 150 mL), brine (50 mL) and dried over cotton. The orange crude product was concentrated and purified by column chromatography (CH_2_Cl_2_/EtOAc: 100/0 to 80/20) to give **2** as a white solid (100 mg, 38%). R_f_ = 0.15 (CH_2_Cl_2_/EtOAc: 80/20). Mp: 226 °C.

**4-((4-hydroxyphenyl)(pyridin-2-yl)methyl)phenyl acetate (3)**

Aq. LiOH (75 mg, 15 mL, 3.13 mmol, 1.13 eq) was added at rt to a solution of **1** (1g, 2.77 mmol, 1 eq) in 1,2-dimethoxyethane (30 mL). The resulting purple solution was further stirred for 1h45 at this temperature, then diluted with H_2_O (5 mL) and extracted with EtOAc (3 x 75 mL). The organic layers were dried over cotton and concentrated in vacuo. The resulting residue was purified by column chromatography (CH_2_Cl_2_/EtOAc: 100/0 to 80/20) to obtain **3** as a white solid (415 mg, 47 %). R_f_ = 0.43 (CH_2_Cl_2_/EtOAc: 80/20). ^1^H NMR (CDCl_3_): *δ =* 2.29 (s, 3H), 5.63 (s, 1H), 5.91 (s, 1H), 6.66 (d, *J =* 8.5 Hz, 2H), 6.94 (d, *J =* 8.8 Hz, 2H), 7.02 (d, *J =* 8.8 Hz, 2H), 7.08 (d, *J =* 8.1 Hz, 1H), 7.18 (d, *J =* 8.5 Hz, 3H), 7.64 (td, *J =* 7.6 Hz, 2.2 Hz,1H), 8.6 (d, *J =* 5.0 Hz, 1H). ^13^C NMR (CDCl_3_): *δ =* 21.2, 57.3, 115.6, 121.4, 121.7, 123.9, 130.2, 130.3, 137.1, 140.2, 148.9, 149.3, 155, 163.4.

**4,4'-dimethoxydiphenyl(pyridin-2-yl)methane (4).**

DDPM **2** (134 mg, 483 μmol, 1 eq) was dissolved in DMF (5 mL). Then, NaH (60 % in mineral oil, 60 mg, 1.5 mmol, 3.11 eq) was added portionwise to the solution at 0 °C. The resulting suspension was further stirred 7 min at 0 °C. Then, CH_3_I (120 μL, 1.987 mmol, 4.1 eq) was added dropwise and the mixture further stirred at this temperature during 3 h before being quenched with ~~a~~ sat. aq. NH_4_Cl and water, respectively. CH_2_Cl_2_ and H_2_O were added. The organic layer was separated and further washed with H_2_O, dried over cotton and concentrated in vacuo. Final purification using column chromatography (CH_2_Cl_2_/EtOAc: 100/0 to 80/20) yielded ester **4** as a yellow oil (134 mg, 92 %). ^1^H-NMR (300 MHz, CDCl_3_): *δ =* 3.78 (s, 6H), 5.60 (s, 1H), 6.83 (d, *J* = 8.7 Hz, 4H), 7.09 (d, *J =* 8.7 Hz, 4H), 7.13-7.15 (m, 1H), 7.61 (td, *J* = 7.8, 1.9 Hz, 1H), 8.60 (d, *J* = 4.5 Hz, 1H). ^13^C NMR (100 MHz, CDCl_3_): *δ =* 55.3, 57.5, 113.9, 121.5, 123.8, 130.3, 135.1, 137.0, 149.0, 158.3, 163.7.

**4-hydroxyphenyl-4-methoxyphenyl-(pyridin-2-yl)-methane (5).**

Compound **2** (100 mg, 360.6 μmol, 1 eq) was dissolved in DMF (15 mL). NaH (60% in mineral oil, 16 mg, 400.1 μmol, 1 eq) was then added in portion at 0 °C. After 5 min stirring at this temperature, CH_3_I (22.5 μL, 360.6 μmol, 1 eq) was added. The resulting purple solution was further stirred at 0 °C during 3 h, before being quenched with water (15 mL) and extracted with CH_2_Cl_2_ (30 mL). The organic layer was washed with sat. NaHCO_3_ (20 mL), aq. LiCl (1g in 20 mL), H_2_O (3 x 20 ml) and dried over cotton. The concentrated crude product was purified by flash chromatography (CH_2_Cl_2_/AcOEt: 100/0 to 85/15) to yield titled compound **5** as an orange oil (m = 80 mg, 76 %). R_f_ = 0.20 (CH_2_Cl_2_/EtOAc: 90/10). ^1^H NMR (300 MHz, CDCl_3_): *δ =* 3.77 (s, 3H), 5.63 (s, 1H), 6.48-6.51 (d, J = 8.7 Hz, 2H), 6.76-6.79 (d, *J =* 8.4 Hz, 2H), 6.81-6.84 (d, *J =* 8.7 Hz, 2H), 7.05-7.08 (d, *J =* 8.7 Hz, 3H), 7.17-7.21 (td, *J =* 5.9, 1.6 Hz, 1H), 7.63-7.69 (td, *J =* 7.8, 1.9 Hz, 1H), 8.57-8.58 (d, *J =* 4.1 Hz, 1H). ^13^C NMR (400 MHz, CDCl_3_): *δ =* δ 55.33, 57.16, 113.9, 115.8, 121.8, 124.0, 130.3, 130.4, 133.3, 134.6, 137.5, 148.4, 155.5, 158.3, 148.8, 155.8, 158.3, 164.1.

*The less polar bis-anisole derivative was also isolated during the purification process.*

**4-hydroxyphenyl-4-trifluoromethylsulfatephenyl-(pyridin-2-yl)-methane (S-1).**

Et_3_N (66 mg, 652 μmol, 1.2 eq) and TFAA (160 mg, 567 μmol, 1.05 eq) were subsequently added to a solution of **2** (150 mg, 541 μmol, 1 eq) in CH_2_Cl_2_ (10 mL). Then, the mixture was stirred overnight at rt, before being quenched with sat. aq. Na_2_CO_3_. CH_2_Cl_2_ (100 mL) was added to the solution and the organic layer separated and further washed with H_2_O (200 mL), dried over cotton and concentrated under vacuum. The crude product was purified by column chromatography (CH_2_Cl_2_/EtOAc: 100/0 to 50/50) to yield compound **S-1** as a yellow oil (50 mg, 24 %). ^1^H-NMR (300 MHz, CDCl_3_): *δ =* 6.13 (s, 1H), 6.76 (d, *J* = 8.4 Hz, 2H), 6.91 (d, *J* = 8.4 Hz, 2H), 7.35 (d, *J* = 6.9 Hz, 1H), 7.52(td, *J* = 6.2, 5.8 Hz, 1H), 8.04 (td, *J* = 5.6, 8.1 Hz, 1H), 8.63 (d, *J* = 2.2 Hz, 1H).

**Diphenyl-2-pyridylmethane (6).**

Compound **6** is commercially available from Acros (cat # 38794)

**4-hydroxyphenyl-4-phenyl-(pyridin-2-yl)-methane (7).**

PPh_3_ (9 mg), Pd(OAc)_2_ (7 mg), HCOOH (20 μL) and Et_3_N (48 μL) were added to a solution of compound **S-1** (40 mg, 0.1 mmol, 1 eq) in DMF (1.2 mL). The reaction mixture was then stirred overnight at reflux, before being quenched with sat. aq. Na_2_CO_3._ CH_2_Cl_2_ (50 mL) was added and the organic layer separated and further washed with H_2_O (100 mL), dried over cotton and concentrated in vacuo. Final purification using column chromatography yielded phenol **7** (11 mg, 40 %). ^1^H-NMR (300 MHz, CDCl_3_): *δ =* 5.69 (s, 1H), 6.57 (d, *J* = 8.4 Hz, 2H), 6.86 (d, *J* = 8.1 Hz, 2H), 7.08 (d, *J* = 7.2 Hz, 1H), 7.15-7.32 (m, 7H), 7.67 (td, *J* = 5.6, 5.9 Hz, 1H), 8.60 (d, *J* = 5.0 Hz, 1H).

**4-Methoxyphenyl-(pyridin-2-yl)-methanol (S-3)**^[[5]](#footnote-5)^

Following the procedure described by Shibahara *et al,^5^*  **S-3** was obtained as a beige solid (27 %) after trituration in Et_2_O. All analytical data correspond to those described in the literature.^5^

**2-(4-Methoxybenzyl)pyridine (S-4)**

~~~~Alcohol **S-3** (0.3 mmol) was added to a mixture of conc. aq. HI (300 µL; 50% w/w) and AcOH (1.5 mL). The solution was then stirred at 100 °C for 4 h, before being cooled to 0 °C, basified to pH 9 with aq. NaOH (15 % w/w) and diluted with CH_2_Cl_2_ (50 mL). The organic layer was washed with sat. aq. Na_2_S_2_O_3_ and further dried over anhydrous MgSO_4_. Final purification using column chromatography yielded **S-4** as a yellow oil (quant.). All analysis correspond to those described in the literature.^5^

**2-(4-Hydroxybenzyl)pyridine (8)**

Using general method E, **8** was obtained as white crystals (60 %), after purification using column chromatography (CH_2_Cl_2_/EtOAc: 100/0 to 80/20). ^1^H NMR (300 MHz, CDCl_3_): *δ =* 3.9 (s, 2H), 6.67 (d, *J* = 8.4 Hz, 2H), 7.05 (d, *J* = 8.4 Hz, 2H), 7.21 (m, 2H), 7.68 (td, *J* = 1.6, 7.6 Hz, 1H), 8.46 (d, *J* = 4.8 Hz, 1H), 9.2 (s, OH). ^13^C NMR (400 MHz, CDCl_3_): *δ =* 42.9, 115.1, 121.2, 122.8, 129.8, 129.9, 136.7, 148.9, 155.6, 161.2. HRMS: calcd. for C_12_H_11_NOH [M+H]^+^~~,~~ 186.0918; found 186.0909.

**4-Chloro-4’-methoxybenzophenone (S-7a)**

Using general method B, **S-7a** was isolated as a grey solid (41 %), after precipitation. ^1^H NMR (CDCl_3_): *δ =* 3.87 (s, 3H), 6.94 (d, *J =* 8.7 Hz, 1 H), 7.43 (d, *J* =8.7 Hz, 1 H), 7.68 (d, *J* =8.7 Hz, 1 H), 7.77 (d, J =8.7 Hz, 1 H).

**4-trifluoromethyl-4’-methoxybenzophenone (S-7b)**

Using general method B, **S-7b** was precipitated as a grey solid (55 %). ^1^H NMR (CDCl_3_): *δ =* 3.92 (s, 3 H), 7.00 (d, *J* = 8.7 Hz, 1 H), 7.76 (d, *J* = 8.7 Hz, 1 H), 7.82-7.88 (m, 2 H). ^13^C NMR (CDCl_3_): *δ =* 55.6, 113.8, 123.7, 125.2, 129.4, 129.8, 132.6, 133.3, 141.5, 163.7, 194.3.

**(4-Chlorophenyl)(4-methoxyphenyl)(pyridin-2-yl)methanol (S-8a)**

Using general method B, **S-7b** was precipitated as a grey solid (55 %). ^1^H NMR (CDCl_3_): *δ =* 3.92 (s, 3 H), 7.00 (d, *J* = 8.7 Hz, 1 H), 7.76 (d, *J* = 8.7 Hz, 1 H), 7.82-7.88 (m, 2 H). ^13^C NMR (CDCl_3_): *δ =* 55.6, 113.8, 123.7, 125.2, 129.4, 129.8, 132.6, 133.3, 141.5, 163.7, 194.3.

**(2-hydroxyphenyl)-(4-hydroxyphenyl)-(pyridin-2-yl)-methane (9)**

 Obtained as byproduct during the preparation of **2** (Scheme 2, Route 2): white solid (36 mg, 13 %). R_f_ = 0.63 (CH_2_Cl_2_/EtOAc: 80/20). ^1^H NMR (300 MHz, CD_3_OD): *δ =* 5.79 (s, 1H), 6.66-6.85 (m, 8H), 7.04-7.25 (m, 3H), 7.68-7.73 (td, *J =* 7.8, 1.9 Hz, 1H), 8.41-8.42 (d, *J =* 4.7 Hz, 1H). ^13^C NMR (400 MHz, CD_3_OD): *δ =* 54.17, 116.0, 116.8, 120.3, 122.9, 125.2, 128.9, 130.7, 131.1, 131.5, 134.4, 138.6, 149.2, 156.4, 156.8, 165.0.

**(4-Chlorophenyl)(4-methoxyphenyl)(pyridin-2-yl)methane (S-9a)**

Using general method D, **S-9a** was isolated as a colorless oil (54 %), after purification using column chromatography (heptane/CH_2_Cl_2_: 50/50 to 0/100). ^1^H NMR (CDCl_3_): *δ =* 3.76 (s, 3 H), 5.60 (s, 1 H), 6.82 (d, *J* = 8.8 Hz, 1 H), 7.03-7.11 (m, 3 H), 7.12-7.15 (m, 1 H), 7.23 (d, *J* = 8.4 Hz, 1 H), 7.60 (td, *J* = 1.8, 7.7 Hz, 1 H), 8.61 (d, *J* = 4.9 Hz, 1 H). ^13^C NMR (CDCl_3_): *δ =* 53.6, 55.5, 114.2, 120.0, 124.1, 128.8, 130.4, 130.8, 132.6, 134.3, 137.4, 141.5, 149.1, 158.6, 162.9.

**4-hydroxyphenyl-4-chlorophenyl-pyridin-2-yl-methane (10)**

Using general method E, **10** was obtained as a colorless oil (54 %), after purification using column chromatography (heptane/CH_2_Cl_2_: 50/50 to 0/100). ^1^H NMR (CDCl_3_): *δ =* 5.62 (s, 1 H), 6.55 (d, *J* = 8.6 Hz, 2 H), 6.82 (d, *J* = 8.6 Hz, 2 H), 7.06-7.11 (m, 3 H), 7.23-7.28 (m, 3 H), 7.60 (td, *J* = 1.6, 7.6 Hz, 1 H), 8.61 (d, *J* = 4.8 Hz, 1 H). ^13^C NMR (CDCl_3_) : *δ =* 57.2, 115.8, 122.0, 123.9, 128.6, 130.3, 130.7, 132.5, 132.8, 137.5, 141.0, 148.7, 155.3, 163.1. HRMS: calcd. for C_18_H_14_ClNOH [M+H]^+^~~,~~ 296.0842 [^35^Cl], 298.0842 [^37^Cl]; found 296.0839 [^35^Cl], 298.0811 [^37^Cl].

**4-methoxyphenyl-4-trifluoromethylphenyl-2-pyrimidyl-methane (S-9b)**

Using general method D, **S-9b** was isolated as a colorless oil (87 %), after purification using column chromatography (heptane/CH_2_Cl_2_: 50/50 to 0/100).

**4-hydroxyphenyl-4-trifluoromethylphenyl-2-pyrimidyl-methane (11)**

Using general method E, **11** was obtained as white crystals (70 %), after purification using column chromatography (CH_2_Cl_2_/EtOAc: 100/0 to 80/20). ^1^H NMR (CDCl_3_): *δ =* 5.88 (s, 1 H), 6.61 (d, *J* = 8.5 Hz, 2 H), 6.82 (d, *J* = 8.5 Hz, 2 H), 7.21 (d, *J* = 8.2 Hz, 2 H), 7.28-7.34 (m, 1 H), 7.79 (t, *J* = 7.8 Hz, 1 H), 8.55 (d, *J* = 4.0 Hz, 1 H). HRMS: calcd. for C_19_H_14_F_3_NOH [M+H]^+^~~,~~ 330.1105; found 330.1108.

**4,4’-dihydroxyphenyl-phenyl-methane (12).**

Compound **12** was prepared according to general method A (Scheme 2) as a white solid (traces). R_f_ = 0.38 (CH_2_Cl_2_/MeOH: 95/5). ^1^H NMR (300 MHz, CDCl_3_): *δ =* 5.35 (s, 1H), 6.67-6.70 (d, *J =* 8.4 Hz, 4H), 6.86-6.89 (d, *J =* 8.4 Hz, 4H), 7.04-7.07 (d, *J =* 7.2 Hz, 2H), 7.14-7.16 (t, *J =* 7.2 Hz, 1H), 7.20-7.25 (t, *J =* 7.5 Hz, 2H).

**Bis(4-hydroxyphenyl)methane (13).**

Compound **13** is commercially available from Aldrich (cat # B47006)

**2-(bis(4-acetoxyphenyl)methyl)-1-methylpyridinium iodide (14)**

CH_3_I (732 mg, 5.15 mmol, 18.6 eq) was added to a solution of **1** (100 mg, 0.277 mmol, 1 eq) in acetone (4.20 mL). Then, the reaction mixture was stirred overnight at rt. Evaporation to dryness yielded an orange residue which was purified by column chromatography (CH_2_Cl_2_/MeOH: 100/0 to 96/4) to yield **14** as a yellow solid (49 mg, 33 %). ^1^H NMR (CDCl_3_): *δ =* 2.31 (s, 6H), 4.37 (s, 3H), 6.49 (s, 1H), 7.15 (d, J=8.9 Hz, 4H), 7.37 (d, J=8.7 Hz, 4H), 7.49 (dd, J=8.1Hz, 1.6 Hz, 1H), 8.09 (ddd, J=7.7 Hz, 6.2 Hz,1.6 Hz, 1H), 8.25 (td, J=7.9 Hz, 1.6 Hz , 1H), 8.94 (dd, J=6.3 Hz, 1.2 Hz, 1H).

**4,4'-dihydroxyphenyl-3-pyridinyl-methane (15)**

Prepared according to General method A (Scheme 2). 3-pyridine-carboxyaldehyde **S-11** (100 mg, 0.934 mmol, 1 eq) and phenol (1 g, 10.64 mmol, 11.4 eq) were dissolved in triflic acid (3.39 g, 2 mL, 22.6 mmol, 25 eq). The resulting dark solution was stirred overnight at rt. Then, the reaction mixture was poured onto ice, neutralized with sat. NaHCO_3_ and extracted with EtOAc (3 x 50 mL). The organic layers were combined, dried over medical cotton and concentrated in vacuo. The resulting residue was purified by column chromatography (reversed phase, CH_3_CN/H_2_O: 5/95 to 70/30) to give compound **15** as a white solid (29 mg, 11 %). ^1^H NMR (Aceton-*d_6_*): *δ =* 5.49 (s, 1H), 6.78 (d, *J =* 8.5 Hz, 4H), 6.95 (d, *J =* 8.4 Hz, 4H), 7.28 (dd, *J =* 7.9 Hz, 4.8 Hz, 1H), 7.46 (ddd, *J =* 8 Hz, 2.4 Hz, 1.7 Hz, 1H), 8.28 (s, 2H), 8.4 (m, 2H). ^13^C NMR (Aceton-*d_6_*): *δ =* 53.4, 116.1, 124, 131, 136, 137, 141.5, 148.2, 151.5, 156.8, 205.9, 206.1, 206.3.

**4,4'-dihydroxyphenyl- 4-pyridinyl-methane (16)**

Prepared according to General method A (Scheme 2). 4-pyridinecarboxyaldehyde (**S-12**) (70 mg, 0.654 mmol, 1 eq) and phenol (701 mg, 7.45 mmol, 11.4 eq) were dissolved in triflic acid (2.37 g, 1.4 mL, 15.8 mmol, 25 eq). The resulting dark solution was stirred overnight at rt. Then, the reaction mixture was poured on ice, neutralized with sat. NaHCO_3_ and extracted with EtOAc (3 x 50 mL). The organic layers were combined, dried over cotton and concentrated in vacuo. The resulting residue was purified by column chromatography (reversed phase, CH_3_CN/H_2_O: 5/95 to 70/30) to give **16** as a white solid (27 mg, 15 %). ^1^H NMR (Aceton-*d_6_*) *δ*: 5.43 (s, 1H), 6.79 (d, *J =* 8.5 Hz, 4H), 6.96 (d, *J =* 8.4 Hz, 4H), 7.08 (dd, *J =* 4.4 Hz, 1.9 Hz, 2H), 8.27 (s, 2H), 8.46 (dd, *J =* 4.3 Hz, 1.5 Hz, 2H). ^13^C NMR (CD_3_OD) *δ*: 55.9, 116.3, 126.3, 131.3, 134.9, 149.8, 157.2, 157.3..

**4,4'-dihydroxyphenyl-2-(3-bromopyridinyl)-methane (17)**

****Prepared according to General method A (Scheme 2). 6-bromo-2-pyridinecarboxaldehyde (1 mmol, 1 eq) and phenol (11 mmol, 11 eq) were dissolved in CF_3_SO_3_H (3 mL, 34 mmol, 34 eq). After 3h stirring at rt, the orange mixture was poured onto ice, neutralized with aq. NaOH (15 % w/w) and was extracted with EtOAc (600 mL). The organic layer was washed with H_2_O (450 mL), brine (50 mL), H_2_O (50 mL) and dried over cotton. The orange crude product was concentrated and purified by column chromatography (CH_2_Cl_2_/EtOAc: 100/0 to 80/20) to yield **17** as a white solid (100 mg, 38 %). ^1^H NMR (DMSO-*d_6_*): *δ =* 5.43 (s, 1H), 6.79 (d, *J =* 8.5 Hz, 4H), 6.96 (d, *J =* 8.4 Hz, 4H), 7.08 (dd, *J =* 4.4 Hz, 1.9 Hz, 2H), 8.27 (s, 2H), 8.46 (dd, *J =* 4.3 Hz, 1.5 Hz, 2H). ^13^C NMR (DMSO-*d_6_*): *δ =* 58.3, 116.4, 119.5, 126.9, 131.4, 135.0, 141.0,151.3, 157.3, 164.5.

**4,4'-dihydroxyphenyl-2-(4-bromopyridinyl)-methane (18)**

Prepared according to General method A, Scheme 2****. 4-bromo-2-pyridinecarboxaldehyde (1 mmol, 1 eq) was dissolved in phenol (11 mmol, 11 eq) and CF_3_SO_3_H (3 mL, 34 mmol, 34 eq) was added. After 3h stirring at rt, the orange mixture was poured on ice, neutralized with aq. NaOH (15 % w/w) and extracted with EtOAc (600 mL). The organic layer was washed with H_2_O (450 mL), brine (50 mL), H_2_O (50 mL) and dried over cotton. The orange crude product was concentrated and purified by column chromatography (CH_2_Cl_2_/EtOAc: 100/0 to 80/20) to yield **18** as a white solid (105 mg, 40 %). ^1^H NMR (DMSO-*d_6_*): *δ =* 5.43 (s, 1H), 6.79 (d, *J =* 8.5 Hz, 4H), 6.96 (d, *J =* 8.4 Hz, 4H), 7.08 (dd, *J =* 4.4 Hz, 1.9 Hz, 2H), 8.27 (s, 2H), 8.46 (dd, *J =* 4.3 Hz, 1.5 Hz, 2H). ^13^C NMR (DMSO-*d_6_*): *δ =* 58.3, 116.4, 119.5, 126.9, 131.4, 135.0, 141.0,151.3, 157.3, 164.5.

***tert*-butyl(6-(6-(6-(bis(4-hydroxyphenyl)methyl)pyridin-2-yl)hex-5-ynamido)hexyl)carbamate (19)**

******CuI (6.5 mg, 0.034 mmol, 20 % mol) and Pd(PPh_3_)_2_Cl_2_ (12 mg, 0.017 mmol, 10 % mol) were added to a solution of 4,4'-((6-bromopyridin-2-yl)methylene)diphenol **18** (60 mg, 0.17 mmol) and tert-butyl (6-(hex-5-ynamido)hexyl)carbamate (80 mg, 0.26 mmol, 1.5 eq) in Et_3_N (1 mL). Then, the mixture was stirred overnight at reflux. After concentration under vacuum and purification using column chromatography (CH_2_Cl_2_/EtOAc: 100/0 to 80/40), compound **19** was isolated as a white solid (59 mg, 60 %). ^1^H NMR (CDCl_3_): *δ =* 1.06-1.21 (m, 4H), 1.30-1.38 (m, 13H, 1.74-1.79 (m, 2H), 2.23 (t, *J =* 6.8 Hz, 2H), 2.34 (t, *J =* 5.6 Hz, 2H), 2.86-2.99 (m, 4H), 4.60-4.65 (br m, 1H), 5.49 (s, 1H), 6.25-6.35 (br s, 1H), 6.51 (d, *J =* 8.2 Hz, 4H), 6.67 (d, *J =* 8.2 Hz, 4H), 6.86 (d, *J =* 7.6 Hz, 1H), 7.21 (d, *J =* 7.6 Hz, 1H), 7.54 (t, *J =* 7.8 Hz, 1H), 8.81 (s, 2H). ^13^C NMR (CDCl_3_): *δ =* 18.2, 24.0, 26.2, 26.4, 28.5, 29.5, 30.1, 34.4, 39.4, 40.6, 60.4, 79.7, 115.8, 123.1, 125.1, 130.2, 132.9, 155.5, 156.5, 164.5, 172. HRMS: calcd.for C_35_H_43_N_3_O_5_H [M+H]^+^~~:~~ 586.3275; found 586.3272.

1. Klumpp, D.A. ; Zhang, Y. ; Kindelin, P.J. ; Lau. S. *Tetrahedron* **2006**, 5915-5921. [↑](#footnote-ref-1)
2. Wilkinson, M. C. ; Saez, F.; Hon, W. L. *Synlett* **2006**, *7*, 1063-1066 [↑](#footnote-ref-2)
3. Mameri, S. ; Charbonniere, L.J. ; Ziessel, R.F. *Synthesis* **2003**, 17, 2713-2719. [↑](#footnote-ref-3)
4. Shi, B-F.; Maugel, N.; Zhang, Y-H. Yu J-Q. *Angew. Chem. Int. Ed.* **2008**, 47, 4882 –4886 [↑](#footnote-ref-4)
5. Shibahara, F.; Sugiura, R.; Yamaguchi, E.; Kitagawa, A.; Murai, T**.** *J. Org. Chem.*, **2009**, *74* (9), pp 3566–3568. [↑](#footnote-ref-5)
