# Supplementary material for: Chemical Library Screening and Structure-Function Relationship Studies Identify Bisacodyl as a Potent and Selective Cytotoxic Agent Towards Quiescent Human Glioblastoma Tumor Stem-Like Cells
Source: PLoS One. 2015 Aug 13;10(8):e0134793. doi: 10.1371/journal.pone.0134793 (PMC4536076; doi:10.1371/journal.pone.0134793)

**S7 Methods. LC/MS analytical data**

Compound **1** :


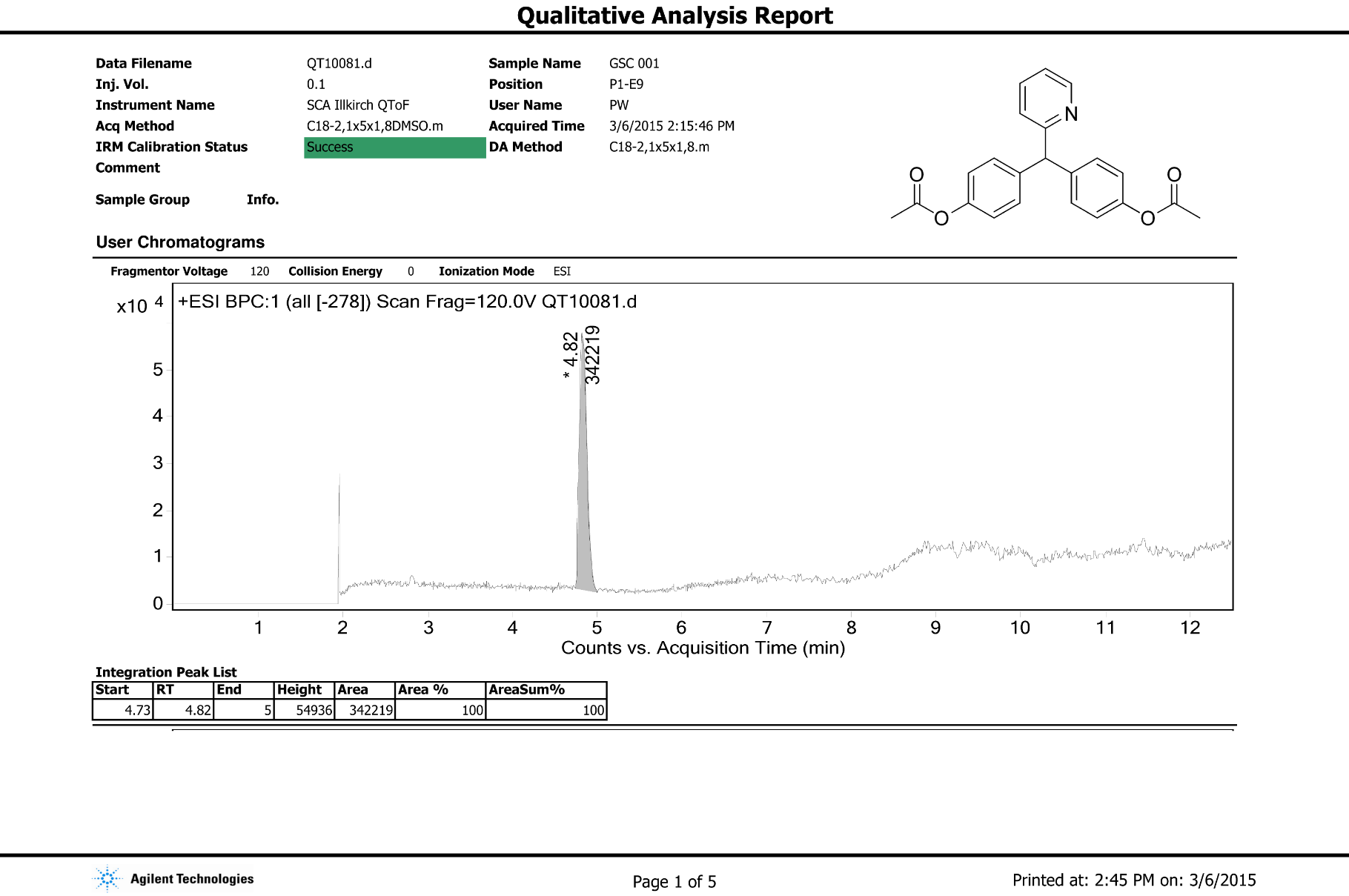


Compound **2**:


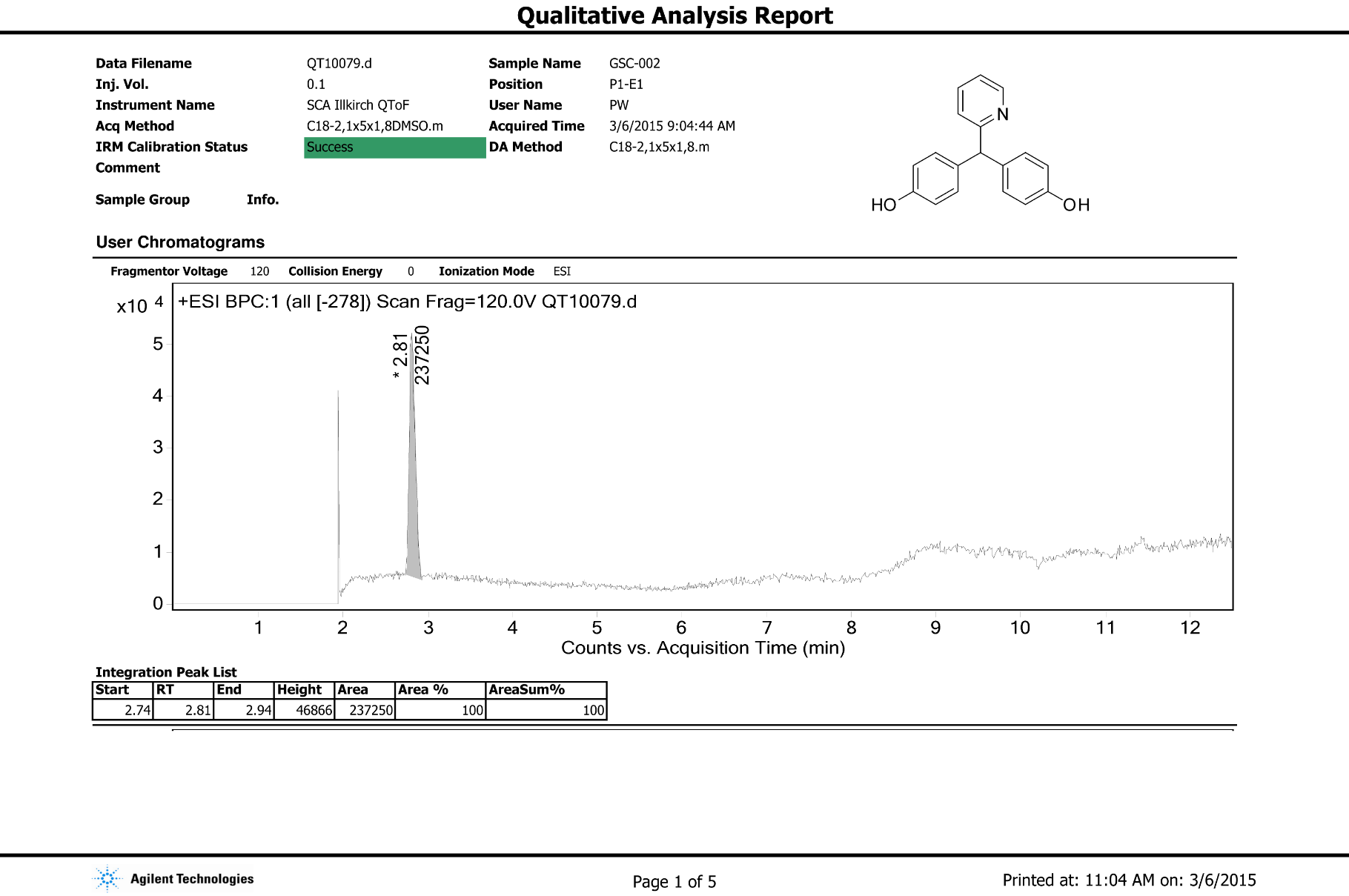


Compound **3** :


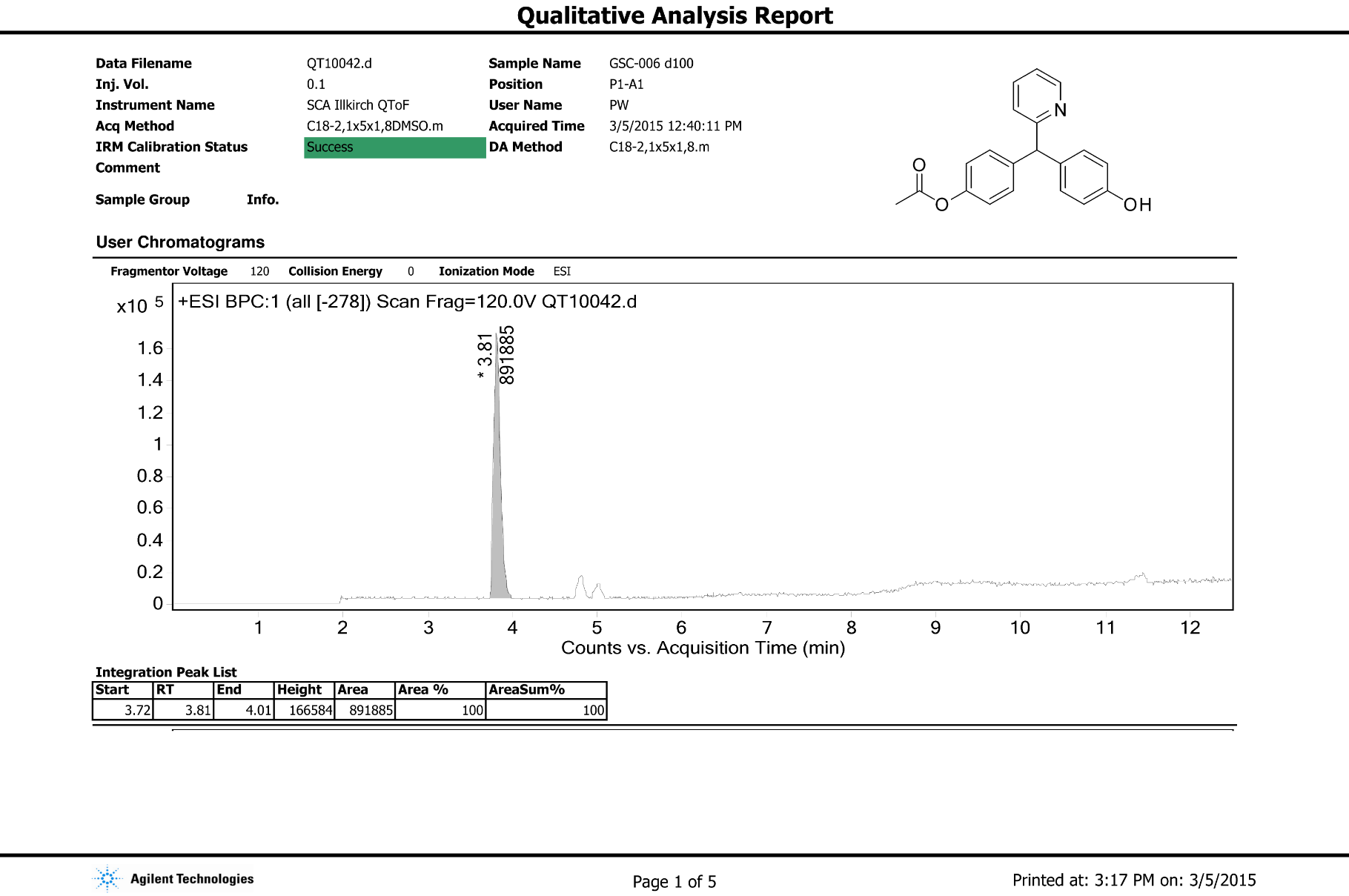


Compound **4** :


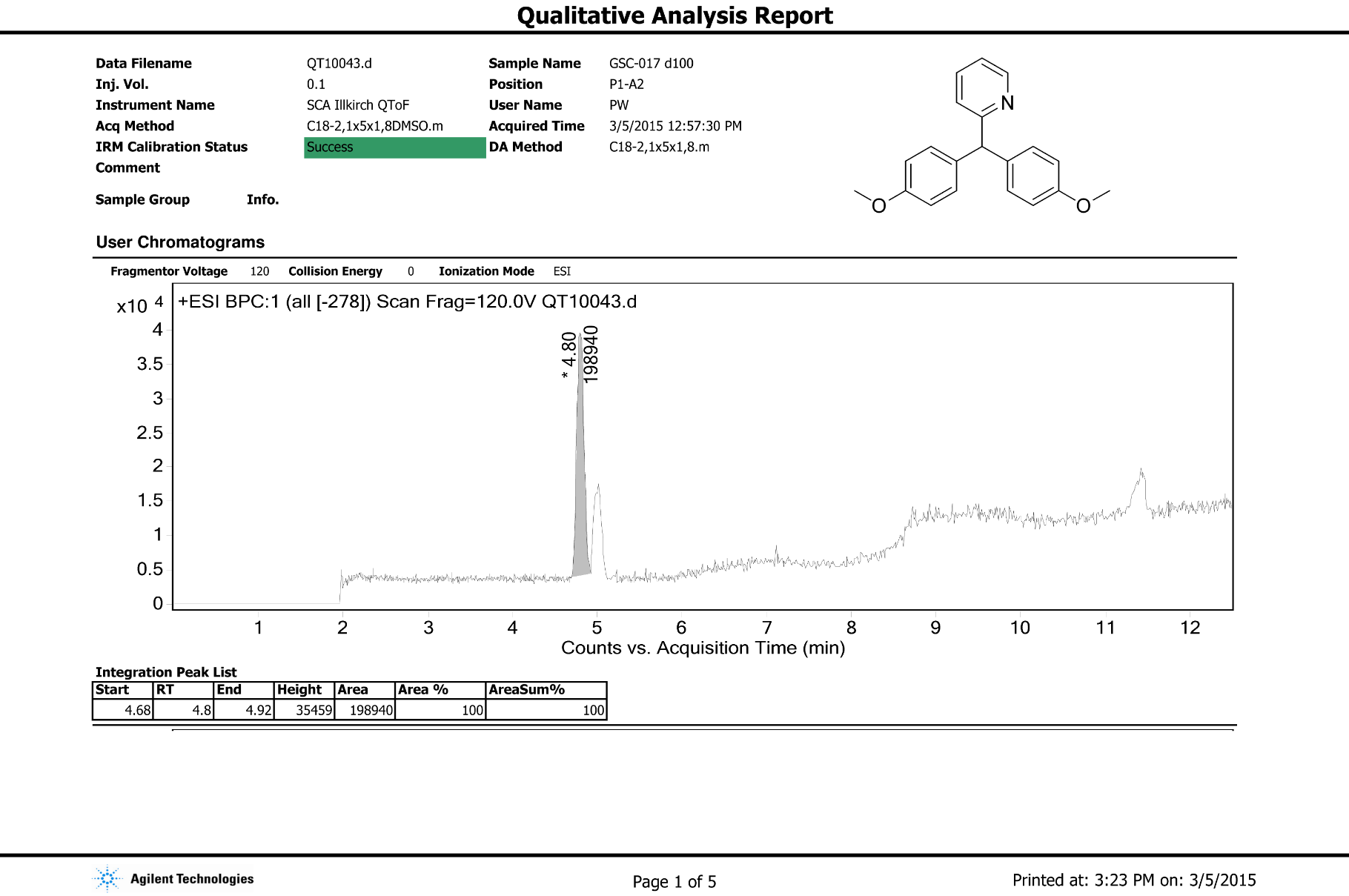


Compound **5** :


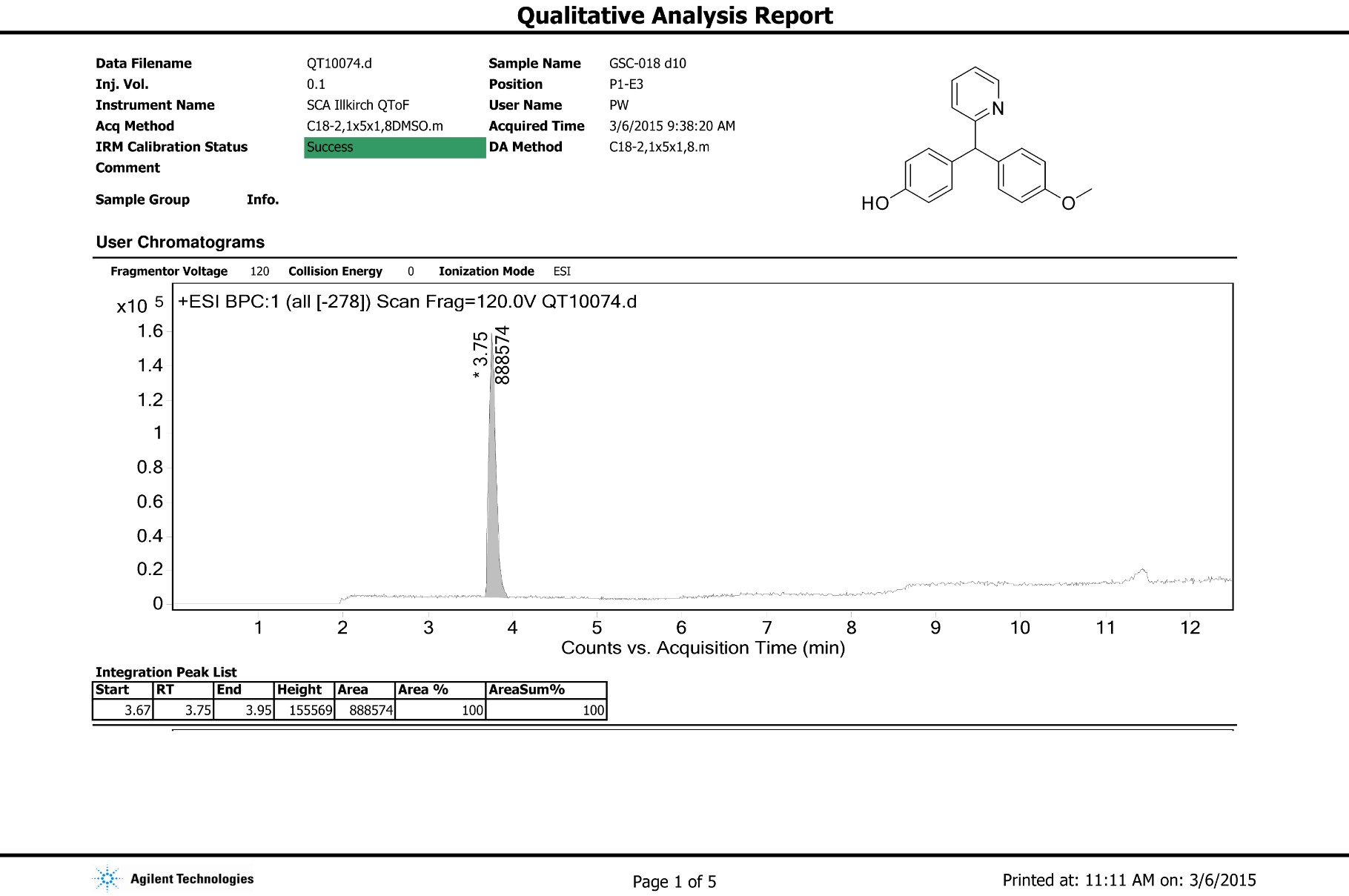


Compound **6** :


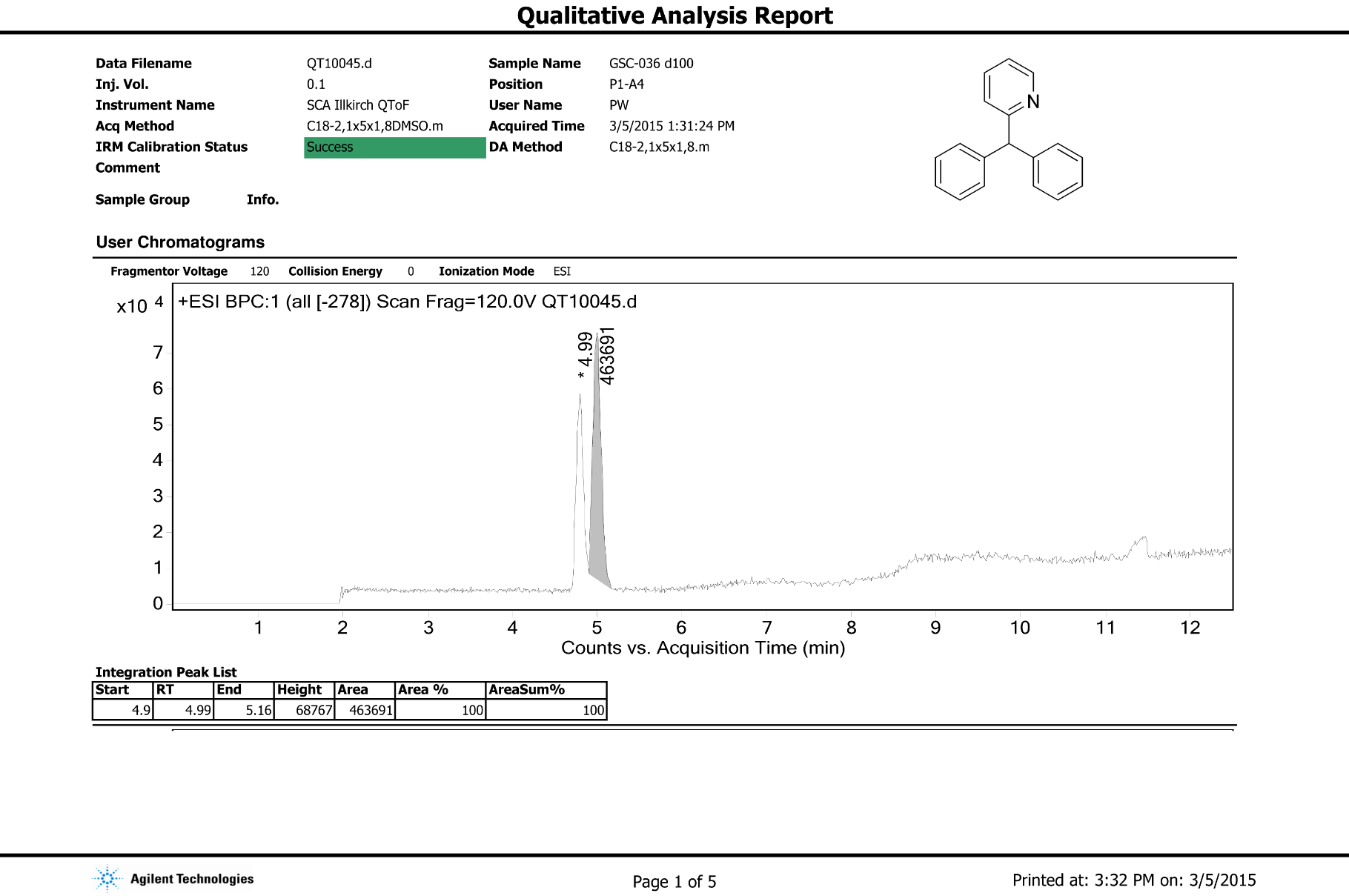


Compound **7**:

| **Compound Label** | **RT** | **Mass** | **Abund** | **Formula** | **Tgt Mass** |  |
| --- | --- | --- | --- | --- | --- | --- |
| Cpd 1: C18H15NO | 3,735 | 261,1155 | 196408 | C18H15NO | NaN |  |

Compound **8** :

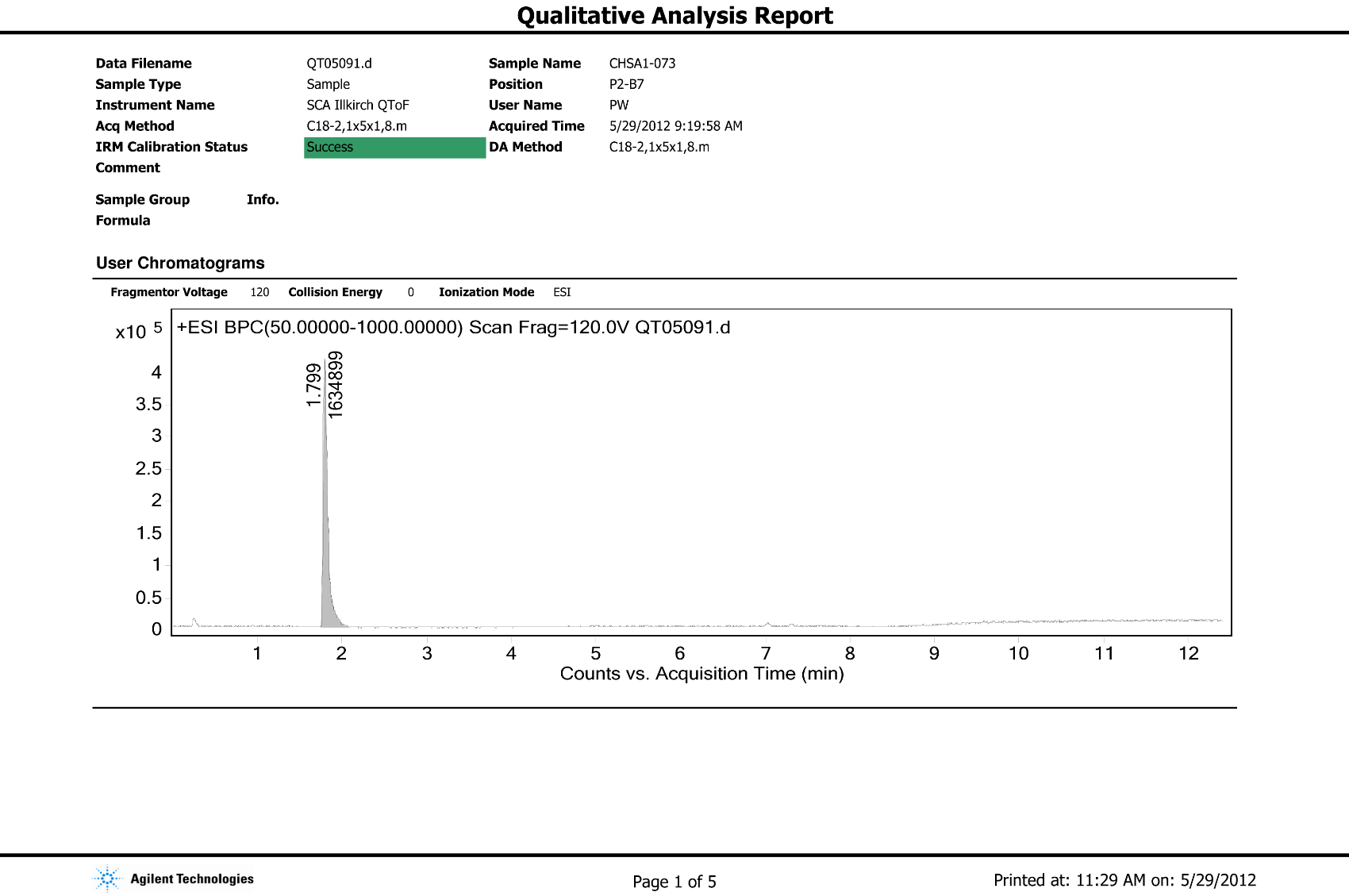


Compound **9** :

| **Sample Name** | | | | GSC-066 | | | | | | **Position** | | | | P1-A9 | | | | | **Instrument Name** | | | | | | | SCA Illkirch QToF | | | | | \|  \| \| --- \| |  |  |  |  |  |  |  |  |  |
| --- | --- | --- | --- | --- | --- | --- | --- | --- | --- | --- | --- | --- | --- | --- | --- | --- | --- | --- | --- | --- | --- | --- | --- | --- | --- | --- | --- | --- | --- | --- | --- | --- | --- | --- | --- | --- | --- | --- | --- | --- | --- |
| **User Name** | | | | PW | | | | | | **Inj Vol** | | | | 0.1 | | | | | **IRM Calibration Status** | | | | | | | Success | | | | |  |  |  |  |  |  |  |  |  |  |
| **Data Filename** | | | | QT05259.d | | | | | | **ACQ Method** | | | | C18-2,1x5x1,8.m | | | | | **Comment** | | | | | | |  |  |  |  |  |  |  |  |  |  |  |  |  |  |  |
| **Acquired Time** | | | | 7/2/2012 2:30:24 PM | | | | | |  |  |  |  |  |  |  |  |  |  |  |  |  |  |  |  |  |  |  |  |  |  |  |  |  |  |  |  |  |  |  |
|  |  |  |  |  |  |  |  |  |  |  |  |  |  |  |  |  |  |  |  |  |  |  |  |  |  |  |  |  |  |  |  |  |  |  |  |  |  |  |  |  |
| **Compound Label** | | | | | **RT** | | **Mass** | | | **Abund** | | **Formula** | | | **Tgt Mass** | | |  |  |  |  |  |  |  |  |  |  |  |  |  |  |  |  |  |  |  |  |  |  |  |
| Cpd 1: C18H15NO2 | | | | | 3,285 | | 277,11056 | | | 374227 | | C18H15NO2 | | | NaN | | |  |  |  |  |  |  |  |  |  |  |  |  |  |  |  |  |  |  |  |  |  |  |  |

Compound **10** :

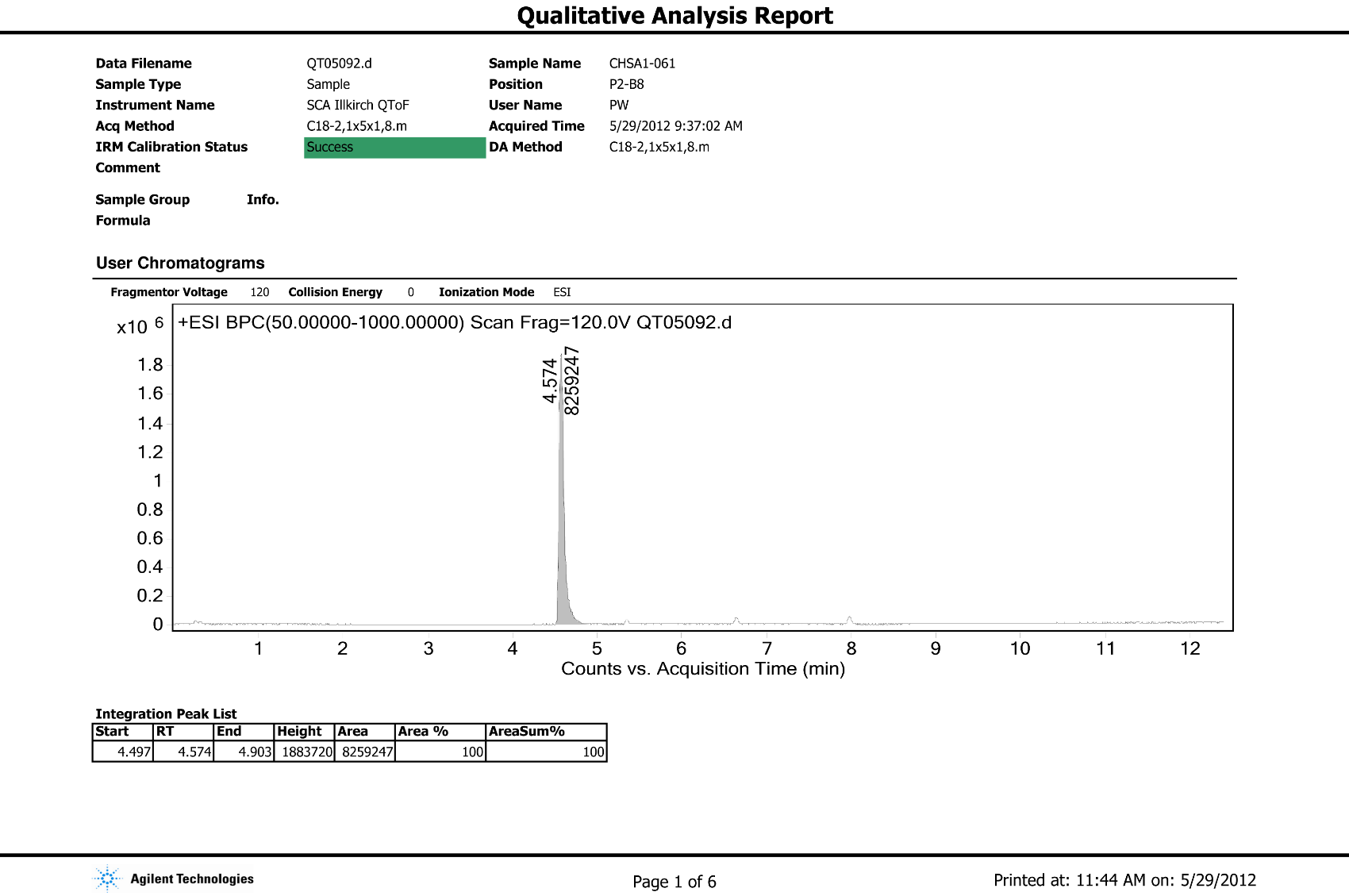


Compound **11**:

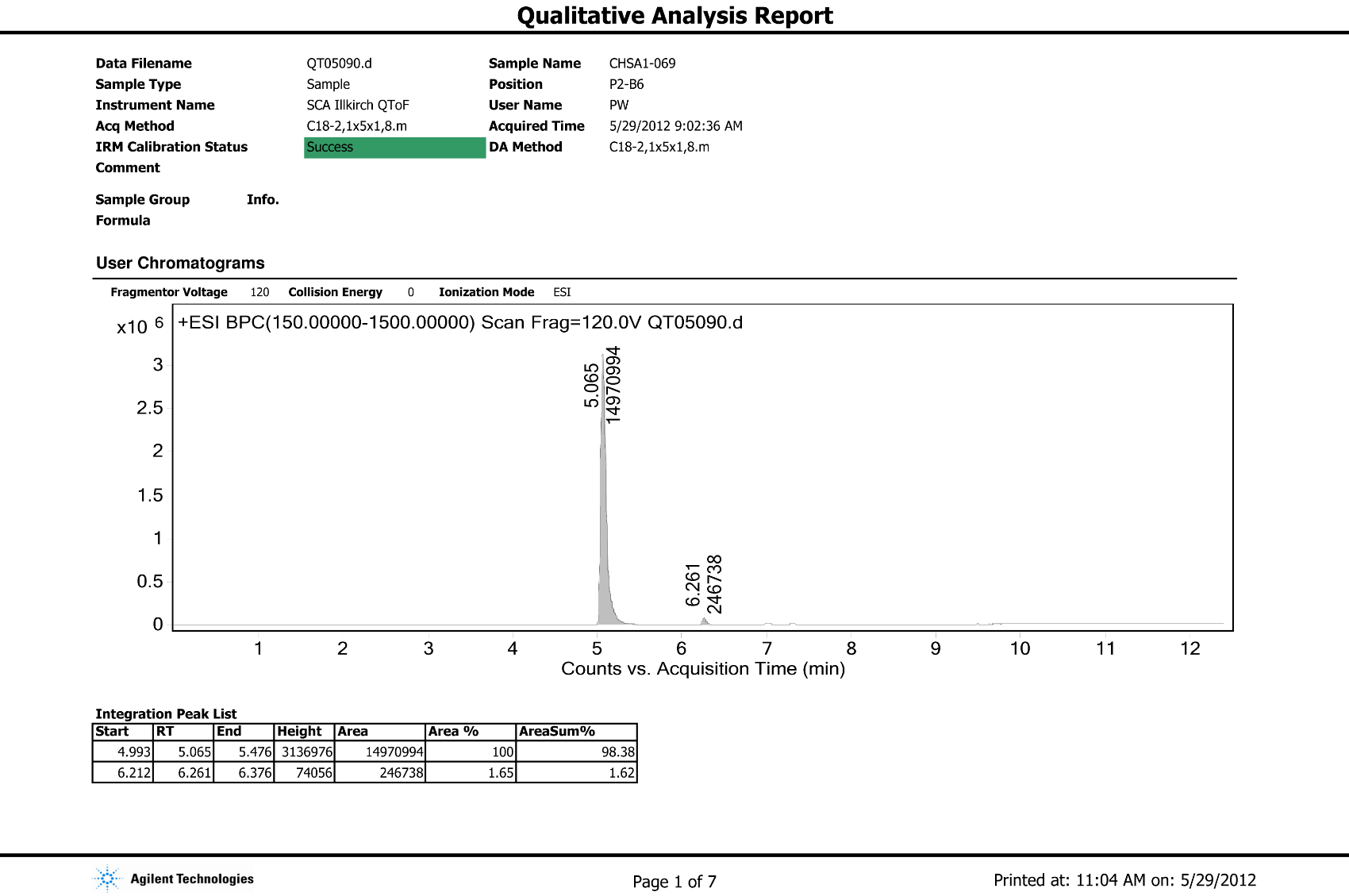


Compound **12** :

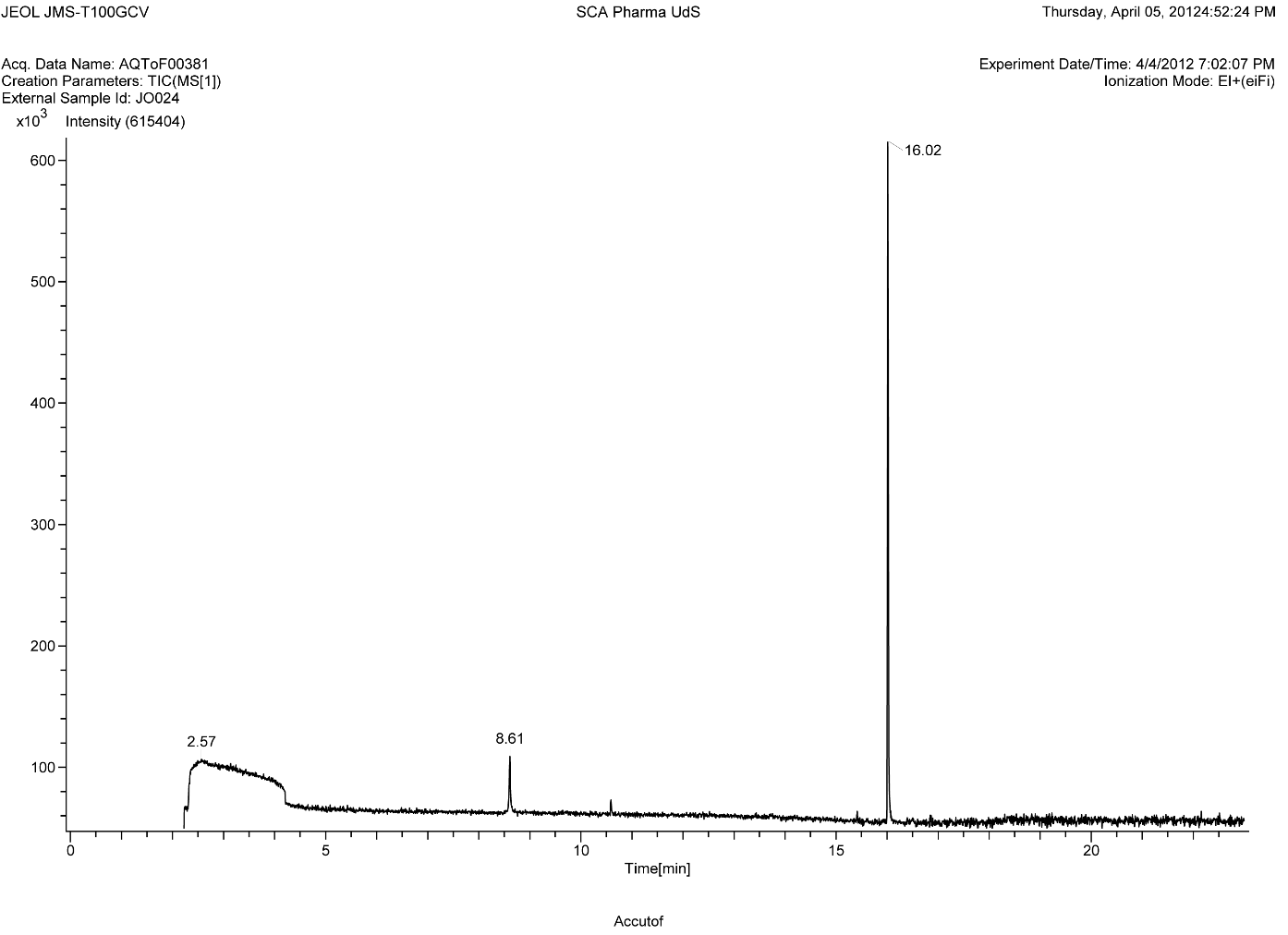


Compound **14** :

|  |  |  |  |  |  |  |  |  |  |  |  |  |  |  |  |  |  |  |  |  |  |  |  |  |  |  |  |  |  |  |  |  |  |  |  |
| --- | --- | --- | --- | --- | --- | --- | --- | --- | --- | --- | --- | --- | --- | --- | --- | --- | --- | --- | --- | --- | --- | --- | --- | --- | --- | --- | --- | --- | --- | --- | --- | --- | --- | --- | --- |
| **Data Filename** | | | | | | | QT05272.d | | | | | | **Sample Name** | | | | GSC-010 | | | | | |  |  | | | | | | | | | |  |  |
| **Inj. Vol.** | | | | | | | 0.1 | | | | | | **Position** | | | | P1-C4 | | | | | |  |  |  |  |  |  |  |  |  |  |  |  |  |
| **Instrument Name** | | | | | | | SCA Illkirch QToF | | | | | | **User Name** | | | | PW | | | | | |  |  |  |  |  |  |  |  |  |  |  |  |  |
| **Acq Method** | | | | | | | C18-2,1x5x1,8.m | | | | | | **Acquired Time** | | | | 7/2/2012 6:12:49 PM | | | | | |  |  |  |  |  |  |  |  |  |  |  |  |  |
| **IRM Calibration Status** | | | | | | | Success | | | | | | **DA Method** | | | | C18-2,1x5x1,8.m | | | | | |  |  |  |  |  |  |  |  |  |  |  |  |  |
| **Comment** | | | | | | |  |  |  |  |  |  |  |  |  |  |  |  |  |  |  |  |  |  |  |  |  |  |  |  |  |  |  |  |  |
| Column3 | | | | | Column4 | | | | | | Column3 | | | | |  |  |  |  |  |  |  |  |  |  |  |  |  |  |  |  |  |  |  |  |
| **Sample Group** | | | | |  | | | | | | **Info.** | | | | |  |  |  |  |  |  |  |  |  |  |  |  |  |  |  |  |  |  |  |  |
| **Formula** | | | | | C23H22INO4 | | | | | |  | | | | |  |  |  |  |  |  |  |  |  |  |  |  |  |  |  |  |  |  |  |  |
|  | | | | |  | | | | | |  | | | | |  |  |  |  |  |  |  |  |  |  |  |  |  |  |  |  |  |  |  |  |

Compound **15** :

| **Sample Name** | | | | GSC-003 | | | | | | **Position** | | | | P1-B5 | | | | | **Instrument Name** | | | | | | | SCA Illkirch QToF | | | | | \|  \| \| --- \| |  |  |  |  |  |  |  |
| --- | --- | --- | --- | --- | --- | --- | --- | --- | --- | --- | --- | --- | --- | --- | --- | --- | --- | --- | --- | --- | --- | --- | --- | --- | --- | --- | --- | --- | --- | --- | --- | --- | --- | --- | --- | --- | --- | --- | --- |
| **User Name** | | | | PW | | | | | | **Inj Vol** | | | | 0.1 | | | | | **IRM Calibration Status** | | | | | | | Success | | | | |  |  |  |  |  |  |  |  |
| **Data Filename** | | | | QT05264.d | | | | | | **ACQ Method** | | | | C18-2,1x5x1,8.m | | | | | **Comment** | | | | | | |  |  |  |  |  |  |  |  |  |  |  |  |  |
| **Acquired Time** | | | | 7/2/2012 3:55:43 PM | | | | | |  |  |  |  |  |  |  |  |  |  |  |  |  |  |  |  |  |  |  |  |  |  |  |  |  |  |  |  |  |
|  |  |  |  |  |  |  |  |  |  |  |  |  |  |  |  |  |  |  |  |  |  |  |  |  |  |  |  |  |  |  |  |  |  |  |  |  |  |  |
| **Compound Label** | | | | | **RT** | | **Mass** | | | **Abund** | | **Formula** | | | **Tgt Mass** | | |  |  |  |  |  |  |  |  |  |  |  |  |  |  |  |  |  |  |  |  |  |
| Cpd 1: C18H15NO2 | | | | | 3,286 | | 277,10963 | | | 3228 | | C18H15NO2 | | | NaN | | |  |  |  |  |  |  |  |  |  |  |  |  |  |  |  |  |  |  |  |  |  |

Compound **16** :


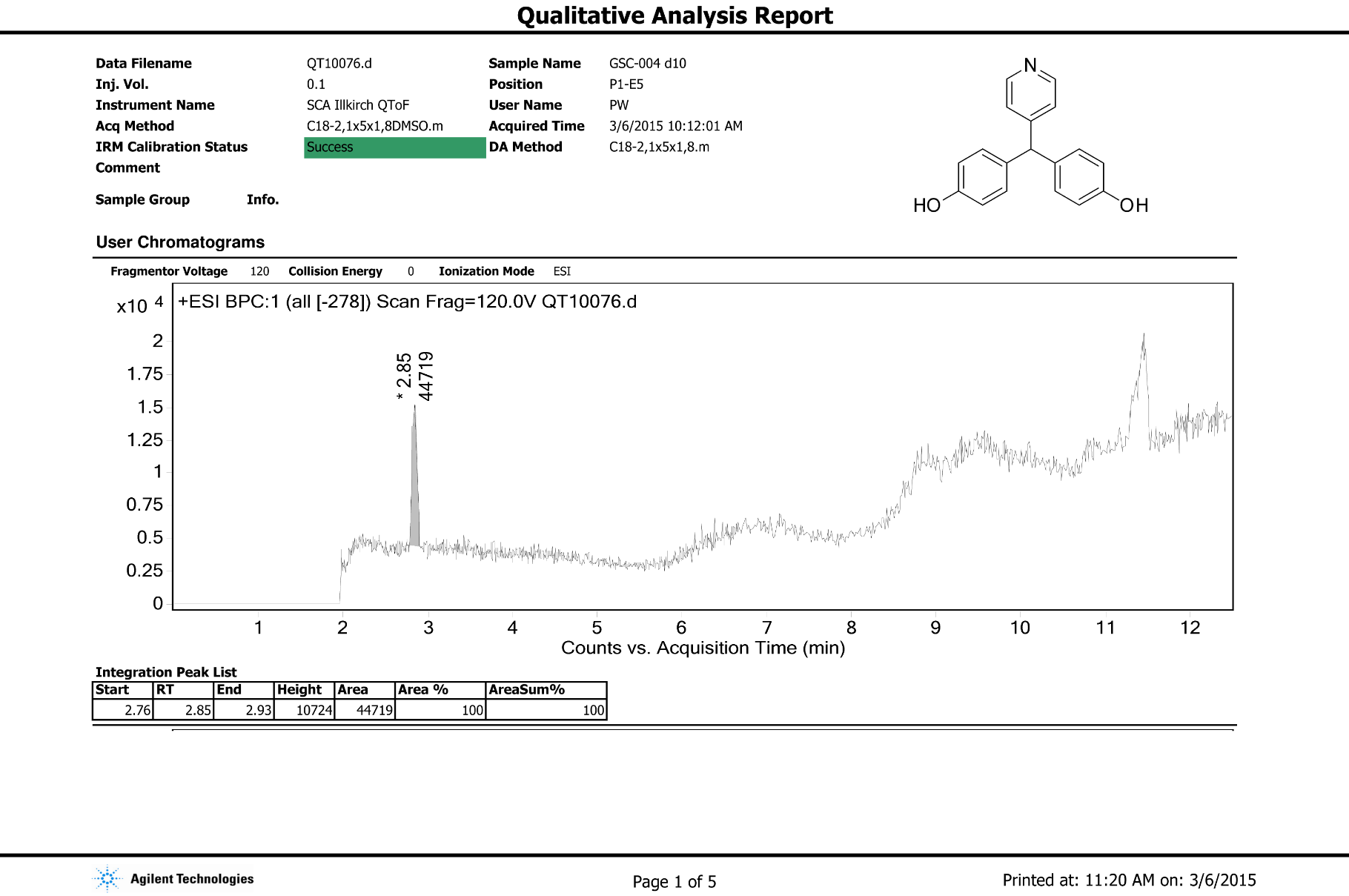


Compound **17** :

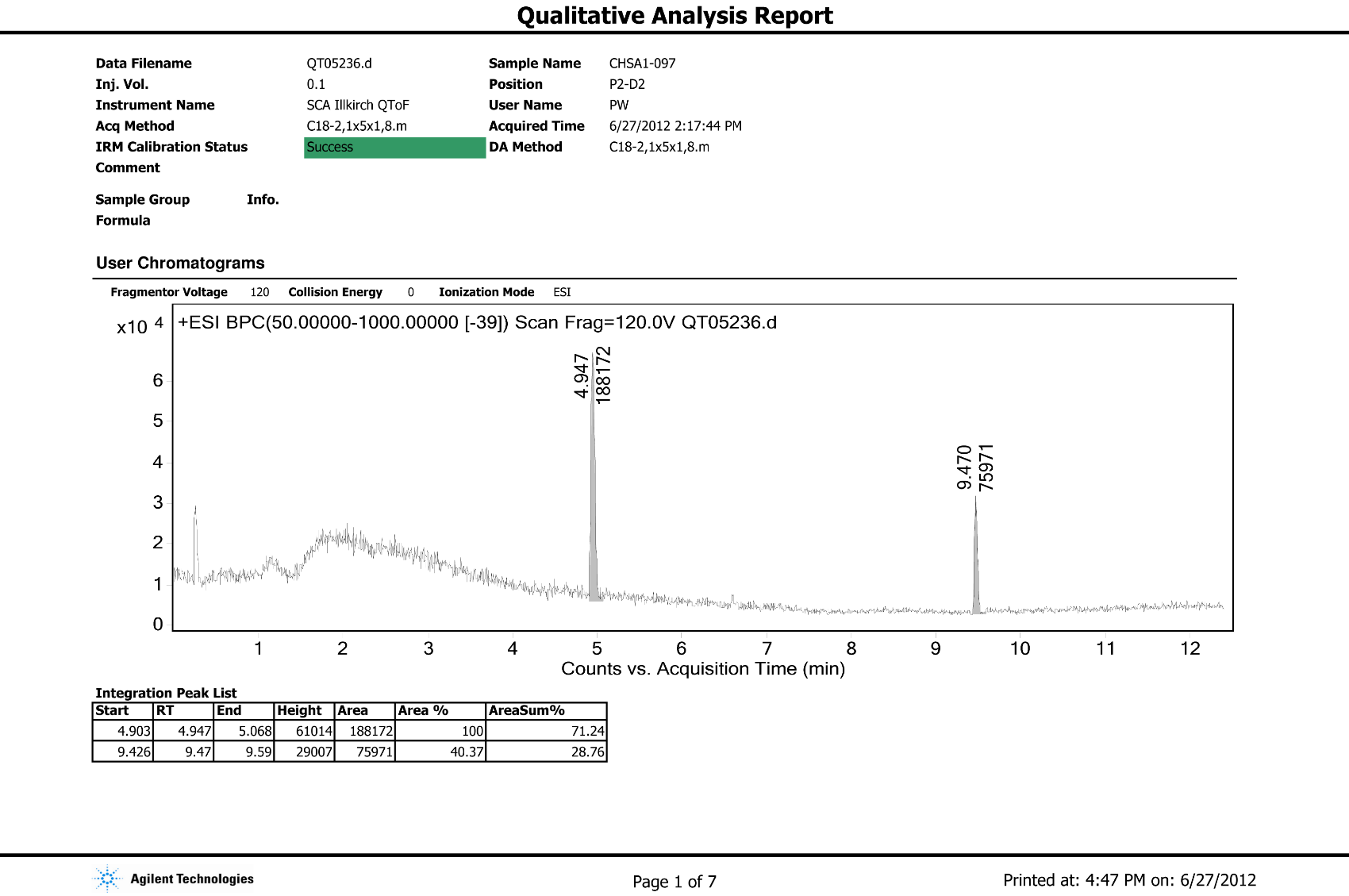


Compound **18** :

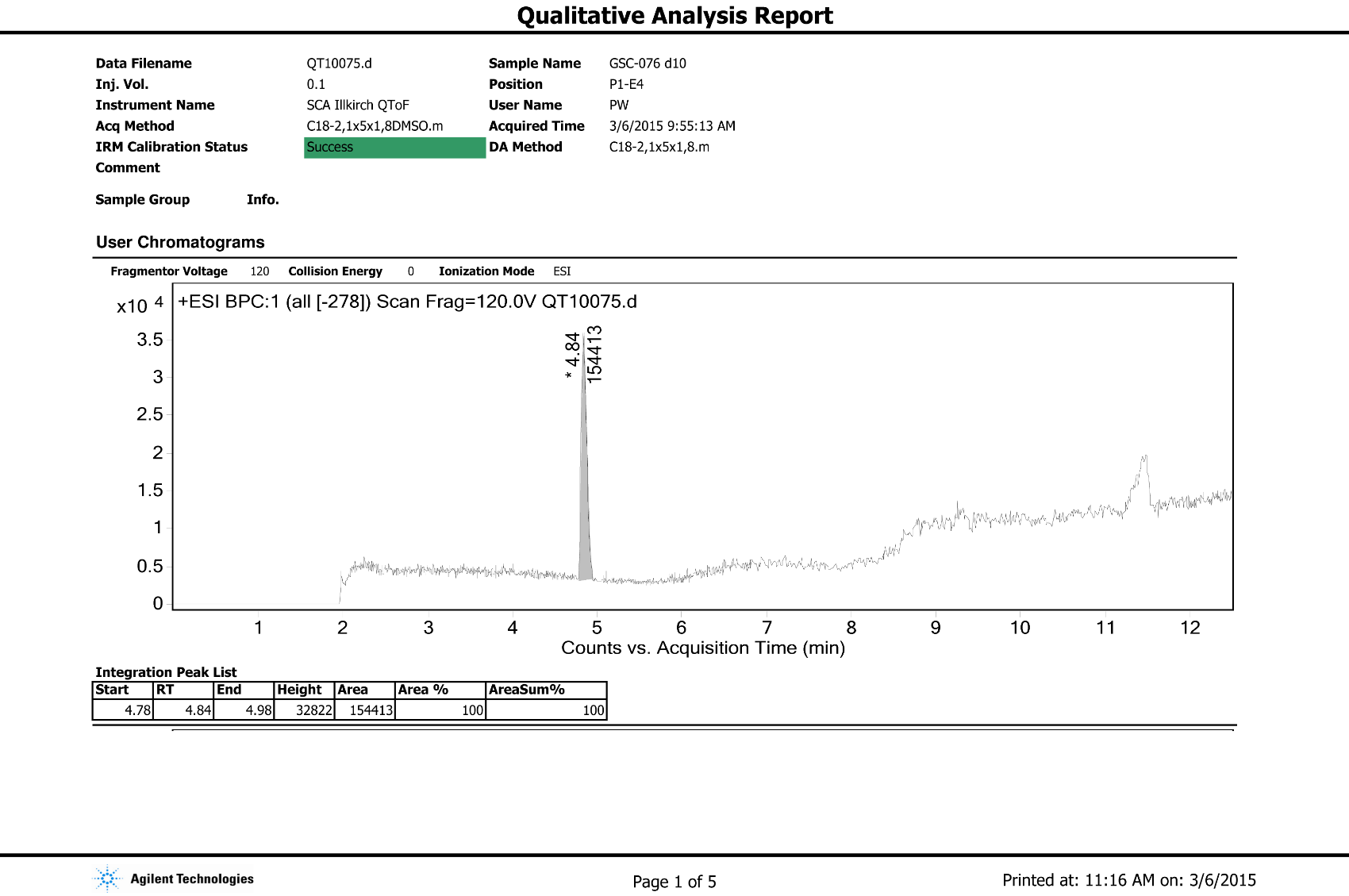


Compound **19** :

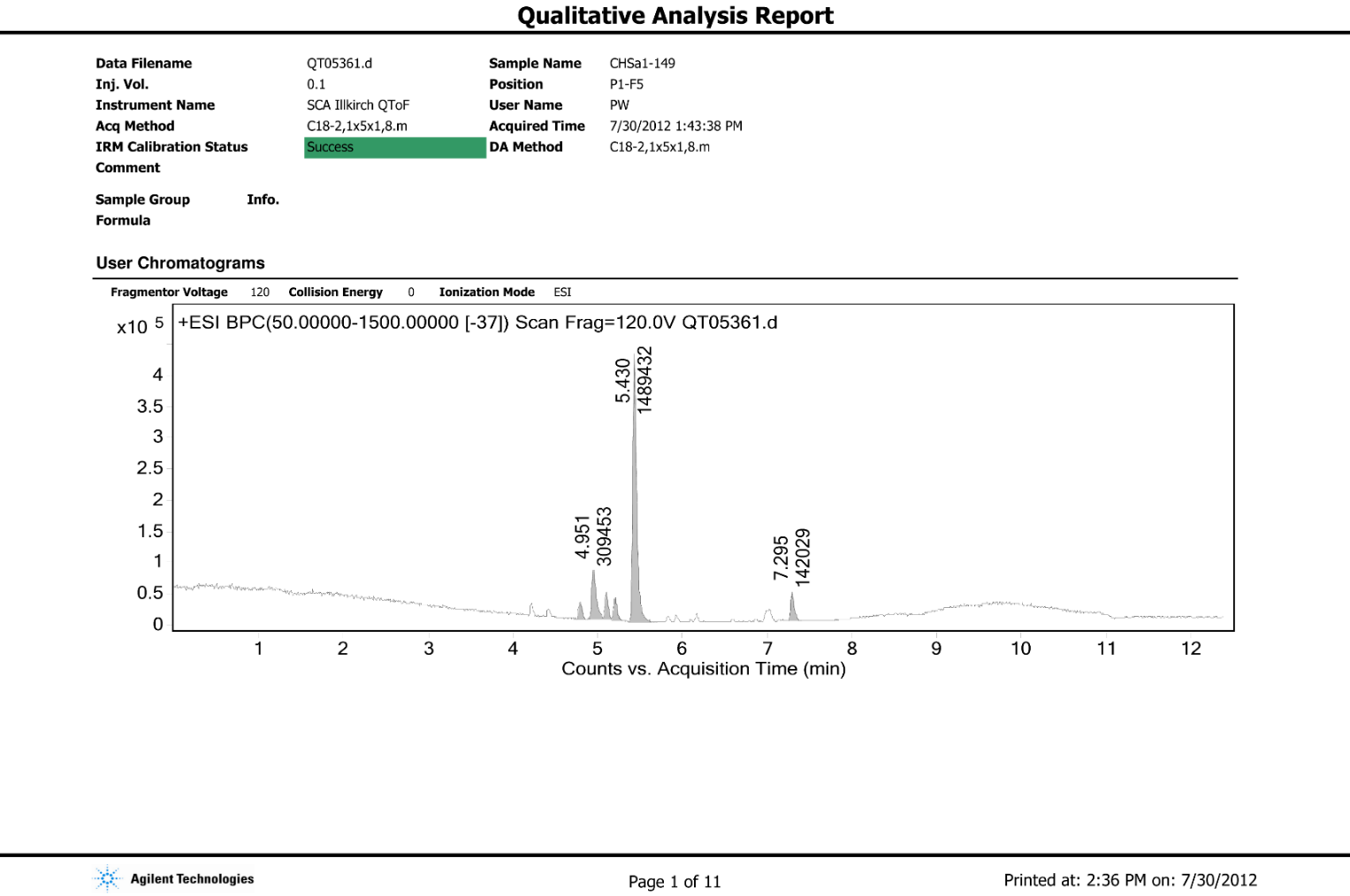

Supplement: S7 Methods — (DOCX) [file pone.0134793.s011.docx]
