# Supplementary material for: Chemical Library Screening and Structure-Function Relationship Studies Identify Bisacodyl as a Potent and Selective Cytotoxic Agent Towards Quiescent Human Glioblastoma Tumor Stem-Like Cells
Source: PLoS One. 2015 Aug 13;10(8):e0134793. doi: 10.1371/journal.pone.0134793 (PMC4536076; doi:10.1371/journal.pone.0134793)
Supplement: S1 Table — P: proliferating; Q: quiescent. (DOCX) [file pone.0134793.s012.docx]

**S1 Table. Cell source, cell handling and resource sharing information.**

| **Cell name** | **Cell type** | **Cell source** | **Cell handling** | **Resource sharing information** |
| --- | --- | --- | --- | --- |
| P TG1, OB1 and TG16 | Proliferating glioblastoma cancer stem-like cells (GSCs) | Isolated from glioblastoma samples of three adult glioblastoma patients (Patru et al., 2010, BMC Cancer 10:66) | Master and working cell banks; use between  20-30 passages (passage 1: first passage following transfer to our laboratory); 2 passages/week;  Cell validation: (Patru et al., 2010, BMC Cancer 10:66) and this report | Chneiweiss and coll. (Neuroscience Paris Seine - IBPS, CNRS UMR 8246/ Inserm U1130/ UPMC, Paris, France) |
| Q TG1, OB1 and TG16 | Quiescent glioblastoma cancer stem-like cells (GSCs) | Isolated from glioblastoma samples of three adult glioblastoma patients (Patru et al., 2010, BMC Cancer 10:66) and maintained without medium renewal for 9-16 days | Obtained from proliferating GSCs if not renewal of the culture medium for 9-16 days.  Cell validation: this report. |  |
| HA cells | Primary human astrocytes | Commercial; ScienCell Research Laboratories, Carlsbad California | Master and working cell banks; use at a maximum of 10 passages after cell thawing | Commercial; ScienCell Research Laboratories, Carlsbad California |
| HEK 293 cells | Human embryonic kidney 293 cells | Commercial, ATCC CRL-1573 | Master and working cell banks | Commercial, ATCC CRL-1573 |
| f-NSCs | Human fetal neural stem cells | Isolated from human fetal brain at embryonic day 50-55 (Carnegie stage 19-22) (Thirant et al., 2011, PlosOne 6: e16375) | Master and working cell banks; use between  20-30 passages (passage 1: first passage following transfer to our laboratory); 1-2 passages/week | Chneiweiss and coll. (Neuroscience Paris Seine - IBPS, CNRS UMR 8246/ Inserm U1130/ UPMC, Paris, France) |
| U-87 MG | Human glioblastoma cell line | Commercial, ATCC HTB-14™ | Master and working cell banks | Commercial, ATCC HTB-14™ |

P: proliferating; Q: quiescent.
