# Supplementary material for: Chemical Library Screening and Structure-Function Relationship Studies Identify Bisacodyl as a Potent and Selective Cytotoxic Agent Towards Quiescent Human Glioblastoma Tumor Stem-Like Cells
Source: PLoS One. 2015 Aug 13;10(8):e0134793. doi: 10.1371/journal.pone.0134793 (PMC4536076; doi:10.1371/journal.pone.0134793)
Supplement: S2 Table — List of the 90 stem cell or differentiation associated genes and 6 housekeeping genes (ACTB, RAF1, CTNNB1, GAPDH, EEF1A1, 18S) included in the Human Stem Cell Pluripotency Array from Life Technologies. (DOCX) [file pone.0134793.s013.docx]

**S2 Table. Assay ID numbers, IDs and names of genes included in the TaqMan Human Stem Cell Pluripotency Arrays from Applied Biosystems, Life Technologies**

| **Life Technologies**  **Assay ID** | **Gene ID** | **Gene name** |
| --- | --- | --- |
|  |  |  |
| Hs99999901_s1 | HSRRN18s | 18S |
| Hs99999905_m1 | 2597 | GAPDH |
| Hs99999903_m1 | 60 | ACTB |
| Hs00170025_m1 | 1499 | CTNNB1 |
| Hs00234119_m1 | 5894 | RAF1 |
| Hs00742749_s1 | 1915 | EEF1A1 |
| Hs00267056_m1 | 3915 | LAMC1 |
| Hs00158620_m1 | 3912 | LAMB1 |
| Hs00538956_m1 | 10644 | IGF2BP2 |
| Hs00707120_s1 | 10763 | NES |
| Hs00829485_sH | 10581 | IFITM2 |
| Hs00233521_m1 | 928 | CD9 |
| Hs00602736_s1 | 6657 | SOX2 |
| Hs00173810_m1 | 10371 | SEMA3A |
| Hs00193638_m1 | 5420 | PODXL |
| Hs00174360_m1 | 3572 | IL6ST |
| Hs00377820_m1 | 10215 | OLIG2 |
| Hs00277509_m1 | 2335 | FN1 |
| Hs00829813_s1 | 5728 | PTEN |
| Hs00217848_m1 | 55299 | BRIX |
| Hs00275636_m1 | 1382 | CRABP2 |
| Hs00201350_m1 | 23412 | COMMD3 |
| Hs00230965_m1 | 2637 | GBX2 |
| Hs00705137_s1 | 8519 | IFITM1 |
| Hs00194498_m1 | 5978 | REST |
| Hs00231692_m1 | 860 | RUNX2 |
| Hs00171876_m1 | 1789 | DNMT3B |
| Hs02387400_g1 | 79923 | NANOG |
| Hs00702808_s1 | 79923 | LIN28A |
| Hs02339499_g1 | 6997 | TDGF1 |
| Hs00169777_m1 | 5175 | PECAM1 |
| Hs00157258_m1 | 1674 | DES |
| Hs00265966_m1 | 2649 | NR6A1 |
| Hs00157674_m1 | 2670 | GFAP |
| Hs00271352_s1 | 9241 | NOG |
| Hs00300531_m1 | 6855 | SYP |
| Hs00159598_m1 | 4760 | NEUROD1 |
| Hs00172692_m1 | 8521 | GCM1 |
| Hs00742896_s1 | 5460 | POU5F1 |
| Hs00241459_m1 | 2562 | GABRB3 |
| Hs00361224_gH | 1082 | CGB |
| Hs00764128_s1 | 10637 | LEFTB |
| Hs00169095_m1 | 3375 | IAPP |
| Hs00165475_m1 | 5265 | SERPINA1 |
| Hs00751752_s1 | 64321 | SOX17 |
| Hs00745761_s1 | 7044 | EBAF |
| Hs00162669_m1 | 7015 | TERT |
| Hs00255287_s1 | 27022 | FOXD3 |
| Hs00170454_m1 | 2250 | FGF5 |
| Hs00164004_m1 | 1277 | COL1A1 |
| Hs00232128_m1 | 3110 | HLXB9 |
| Hs00232708_m1 | 29842 | TFCP2L1 |
| Hs00744391_s1 | 3050 | HBZ |
| Hs00917999_g1 | 2886 | GRB7 |
| Hs00187067_m1 | 2494 | NR5A2 |
| Hs00240871_m1 | 5080 | PAX6 |
| Hs00171403_m1 | 2626 | GATA4 |
| Hs00156373_m1 | 947 | CD34 |
| Hs00747497_g1 | 8433 | UTF1 |
| Hs00544355_m1 | 51083 | GAL |
| Hs00538143_m1 | 50511 | SYCP3 |
| Hs00174344_m1 | 1003 | CDH5 |
| Hs00158730_m1 | 3977 | LIFR |
| Hs00232018_m1 | 2627 | GATA6 |
| Hs00606316_m1 | 70 | ACTC1 |
| Hs00251859_m1 | 54514 | DDX4 |
| Hs00173014_m1 | 5078 | PAX4 |
| Hs00173490_m1 | 174 | AFP |
| Hs00230919_m1 | 1045 | CDX2 |
| Hs00156568_m1 | 1280 | COL2A1 |
| Hs00172872_m1 | 8320 | EOMES |
| Hs00173564_m1 | 2249 | FGF4 |
| Hs00176573_m1 | 2321 | FLT1 |
| Hs00232764_m1 | 3170 | FOXA2 |
| Hs00174967_m1 | 2641 | GCG |
| Hs00220998_m1 | 9573 | GDF3 |
| Hs00747223_g1 | 3043 | HBB |
| Hs00355773_m1 | 3630 | INS |
| Hs00236830_m1 | 3651 | IPF1 |
| Hs00158126_m1 | 3670 | ISL1 |
| Hs00174029_m1 | 3815 | KIT |
| Hs00196158_m1 | 3848 | KRT1 |
| Hs00300550_m1 | 284217 | LAMA1 |
| Hs00271574_m1 | 4617 | MYF5 |
| Hs00159528_m1 | 4654 | MYOD1 |
| Hs00415443_m1 | 4838 | NODAL |
| Hs00383230_g1 | 4878 | NPPA |
| Hs00603586_g1 | 256297 | PTF1A |
| Hs00293258_m1 | 6423 | SFRP2 |
| Hs00174949_m1 | 6750 | SST |
| Hs00610080_m1 | 6862 | T |
| Hs00356930_m1 | 6898 | TAT |
| Hs00165941_m1 | 7054 | TH |
| Hs00240913_m1 | 7490 | WT1 |
| Hs01079824_m1 | 7503 | Xist |
| Hs00399279_m1 | 132625 | ZFP42 |

List of the 90 stem cell or differentiation associated genes and 6 housekeeping genes (ACTB, RAF1, CTNNB1, GAPDH, EEF1A1, 18S) included in the Human Stem Cell Pluripotency Array from Life Technologies.
