# Supplementary material for: Chemical Library Screening and Structure-Function Relationship Studies Identify Bisacodyl as a Potent and Selective Cytotoxic Agent Towards Quiescent Human Glioblastoma Tumor Stem-Like Cells
Source: PLoS One. 2015 Aug 13;10(8):e0134793. doi: 10.1371/journal.pone.0134793 (PMC4536076; doi:10.1371/journal.pone.0134793)
Supplement: S3 Table — GSCs: glioblastoma stem-like cells. Hit compounds selected both in the primary and secondary screens for their activity in at least one of the conditions tested are highlighted in yellow. (DOCX) [file pone.0134793.s014.docx]

**S3 Table. Primary and secondary screen hit compounds.**

| **Hit compounds reducing ATP levels** | **Activity on proliferating TG1 GSCs** | **Activity on quiescent TG1 GSCs** |
| --- | --- | --- |
| **Compound name** |  |  |
| Diflunisal |  | yes |
| Niclosamide |  | yes |
| Miconazole | yes | yes |
| Thioridazine hydrochloride | yes |  |
| Lynestrenol | yes |  |
| Tomatine | yes | yes |
| Perphenazine | yes |  |
| Mefloquine hydrochloride | yes | yes |
| Isoconazole | yes | yes |
| Astemizole | yes |  |
| Terfenadine | yes | yes |
| Chlorhexidine | yes |  |
| Tamoxifen citrate | yes | yes |
| Ivermectin | yes | yes |
| Flutamide |  | yes |
| Flufenamic acid |  | yes |
| Tolfenamic acid |  | yes |
| Antimycin A |  | yes |
| Ethacrynic acid |  | yes |
| Clotrimazol | yes | yes |
| Fendiline hydrochloride |  | yes |
| Perhexiline maleate | yes | yes |
| Econazole nitrate |  | yes |
| Flunarizine dihydrochloride |  | yes |
| Clofilium tosylate |  | yes |
| Lidoflazine | yes |  |
| GBR 12909 dihydrochloride | yes | yes |
| Clomiphene citrate (Z, E) | yes | yes |
| Butoconazole nitrate | yes | yes |
| Amiodarone hydrochloride | yes | yes |
| Bisacodyl |  | yes |
| Suloctidil | yes | yes |
| Cephaeline dihydrochloride heptahydrate | yes | yes |
| Digitoxigenin |  | yes |
| Digoxin | yes |  |
| Meclocycline sulfosalicylate |  | yes |
| Meclozine dihydrochloride | yes | yes |
| Menadione | yes | yes |
| Strophantine octahydrate |  | yes |
| Primaquine diphosphate | yes | yes |
| Progesterone |  | yes |
| Felodipine | yes | yes |
| Metixene hydrochloride | yes |  |
| Norcyclobenzaprine | yes |  |
| Nystatine |  | yes |
| Parthenolide | yes | yes |
| Nitrofuroxazide | yes | yes |
| Prenylamine lactate | yes | yes |
| Rescinnamin |  | yes |
| Piperlongumine | yes |  |
| Ellipticine | yes | yes |
| Lanatoside C | yes | yes |
| Avermectin B1 | yes | yes |
| Beta-Escin | yes | yes |
| Gossypol | yes | yes |
| Lycorine hydrochloride | yes | yes |
| Hexestrol |  | yes |
| Methyl benzethonium chloride | yes | yes |
| Benzethonium chloride | yes | yes |
| Strophanthidin |  | yes |
| Ebselen |  | yes |
| Monensin sodium salt |  | yes |
| Dimeclocycline hydrochloride | yes |  |
| Piperacillin sodium salt | yes |  |
| Diethylstilbestrol | yes | yes |
| Alexidine dihydrochloride | yes | yes |
| Sulconazole nitrate | yes | yes |
| Lasalocid sodium salt | yes | yes |
| Ethaverine hydrochloride |  | yes |
| Indoprofen | yes |  |
| Doxazosin mesylate |  | yes |
| Simvastatin |  | yes |
| Luteolin |  | yes |
| Clioquinol | yes |  |
| Thonzonium bromide | yes | yes |
| Nitrendipine | yes |  |
| Protriptyline hydrochloride | yes |  |
| Prazosin hydrochloride | yes | yes |
| Proscillaridin A |  | yes |
| Sanguinarine | yes | yes |
| Zuclopenthixol hydrochloride | yes |  |
| Sertraline | yes | yes |
| Tribenoside | yes | yes |
| Halofantrine hydrochloride |  | yes |
| Pyvinium pamoate |  | yes |
| Sertaconazole nitrate | yes | yes |
|  |  |  |
| **Hit compounds increasing ATP levels** | **Activity on proliferating TG1 CSCs** | **Activity on quiescent TG1 CSCs** |
| Tranexamic acid |  | yes |
| Scopolamin-N-oxide hydrobromide |  | yes |
| Probucol | yes |  |
| Trimethadione |  | yes |
| Lovastatin |  | yes |
| Rifampicin |  | yes |
| Ethionamide |  | yes |
| (S)-(-)-Atenolol |  | yes |
| Lorglumide sodium salt |  | yes |
| Nitrendipine |  | yes |
| Idazoxan hydrochloride |  | yes |
| Primidone | yes |  |
| Phenoxybenzamine hydrochloride | yes |  |
| Altretamine | yes |  |
| Urosiol | yes |  |
| Trapidil | yes |  |
| Cyclic AMP | yes |  |

GSCs: glioblastoma stem-like cells. Hit compounds selected both in the primary and secondary screens for their activity in at least one of the conditions tested are highlighted in yellow.
